# Identification of an Imidazopyridine-based Compound as an Oral Selective Estrogen Receptor Degrader for Breast Cancer Therapy

**DOI:** 10.1158/2767-9764.CRC-23-0111

**Published:** 2023-07-27

**Authors:** Mengwu Pan, Valeria Solozobova, Nane C. Kuznik, Nicole Jung, Simone Gräßle, Victor Gourain, Yvonne M. Heneka, Christina A. Cramer von Clausbruch, Olaf Fuhr, Ravi S. N. Munuganti, Danilo Maddalo, Christine Blattner, Antje Neeb, Adam Sharp, Laura Cato, Carsten Weiss, Rinath M. Jeselsohn, Veronique Orian-Rousseau, Stefan Bräse, Andrew C. B. Cato

**Affiliations:** 1 Institute of Biological and Chemical Systems – Biological Information Processing, Karlsruhe Institute of Technology, Eggenstein-Leopoldshafen, Germany.; 2 Institute of Biological and Chemical Systems – Functional Molecular Systems, Karlsruhe Institute of Technology, Eggenstein-Leopoldshafen, Germany.; 3 Nantes Université, INSERM, Center for Research in Transplantation and Translational Immunology, UMR 1064, Nantes, France.; 4 Institute of Nanotechnology and Karlsruhe Nano Micro Facility (KNMFi), Karlsruhe Institute of Technology, Eggenstein-Leopoldshafen, Germany.; 5 Vancouver Prostate Centre, Vancouver, British Columbia, Canada.; 6 Institute of Cancer Research, London, United Kingdom.; 7 The Royal Marsden NHS Foundation Trust, Sutton, United Kingdom.; 8 Department of Medical Oncology, Dana-Farber Cancer Institute, Harvard Medical School, Boston, Massachusetts.; 9 Center for Functional Cancer Epigenetics, Dana-Farber Cancer Institute, Boston, Massachusetts.; 10 Institute of Organic Chemistry, Karlsruhe Institute of Technology, Karlsruhe, Germany.

## Abstract

The pro-oncogenic activities of estrogen receptor alpha (ERα) drive breast cancer pathogenesis. Endocrine therapies that impair the production of estrogen or the action of the ERα are therefore used to prevent primary disease metastasis. Although recent successes with ERα degraders have been reported, there is still the need to develop further ERα antagonists with additional properties for breast cancer therapy. We have previously described a benzothiazole compound A4B17 that inhibits the proliferation of androgen receptor–positive prostate cancer cells by disrupting the interaction of the cochaperone BAG1 with the AR. A4B17 was also found to inhibit the proliferation of estrogen receptor—positive (ER^+^) breast cancer cells. Using a scaffold hopping approach, we report here a group of small molecules with imidazopyridine scaffolds that are more potent and efficacious than A4B17. The prototype molecule X15695 efficiently degraded ERα and attenuated estrogen-mediated target gene expression as well as transactivation by the AR. X15695 also disrupted key cellular protein–protein interactions such as BAG1–mortalin (GRP75) interaction as well as wild-type p53–mortalin or mutant p53–BAG2 interactions. These activities together reactivated p53 and resulted in cell-cycle block and the induction of apoptosis. When administered orally to *in vivo* tumor xenograft models, X15695 potently inhibited the growth of breast tumor cells but less efficiently the growth of prostate tumor cells. We therefore identify X15695 as an oral selective ER degrader and propose further development of this compound for therapy of ER^+^ breast cancers.

**Significance:**

An imidazopyridine that selectively degrades ERα and is orally bioavailable has been identified for the development of ER^+^ breast cancer therapeutics. This compound also activates wild-type p53 and disrupts the gain-of-function tumorigenic activity of mutant p53, resulting in cell-cycle arrest and the induction of apoptosis.

## Introduction

Breast cancer is the most commonly diagnosed cancer worldwide with over 2.3 million new cases and 685,000 deaths in 2020. In 2040, the burden of breast cancer is predicted to increase to over 3 million new cases and 1 million deaths every year because of population growth and ageing (1). Approximately 70%–80% of breast cancers are dependent on estrogen receptor (ER) signaling for oncogenic growth and progression (2). Therapies that block the synthesis of estrogens or directly target the ER are highly effective for treating ER-positive (ER^+^) breast cancers. However, acquired resistance to the ER-directed therapies arises that reduce the efficacy of the drugs leading to poor clinical outcomes. The endocrine therapies normally used are compounds that inhibit estrogen biosynthesis (e.g., aromatase inhibitors) or compounds that competitively modulate the action of ERα (e.g., selective ER modulators—SERM). Long-term treatment with the SERM tamoxifen is reported to promote endometrial carcinoma and venous thromboembolism due to partial ERα agonistic activity (3). Second- and third-generation SERMs have therefore been developed that show improved, though distinctly different, safety profiles compared with tamoxifen. However, the risk of venous thromboembolism remains a concern for most SERMs (4). In contrast, selective estrogen receptor degraders (SERD) are considered pure antagonists without agonist activity. They do not only antagonize ERα action but also downregulate ERα protein levels. One of the earliest examples of such a targeted protein degradation therapeutic is fulvestrant, that shows efficacy in tamoxifen-refractory patients and postmenopausal women who had progressed on prior hormone therapies (5) However, fulvestrant's major clinical limitations are its intramuscular route of administration and its low bioavailability (6–8). There is therefore the need for the development of orally bioavailable ER degraders (9). A number of such oral SERDs have been described that exhibit both preclinical and clinical antitumor activities (10, 11) but despite these promising results, there is still an opportunity to develop further ERα antagonists with novel modes of action.

We aim to develop novel ERα inhibitors that do not competitively interact with the ligand-binding domain of the receptors where mutations mostly occur that block the action of the antagonists. Rather, we have embarked on the development of new antagonists that target molecular chaperones and cochaperones that assist the conformational folding or unfolding as well as the assembly of other macromolecular structures including steroid receptors. One such attractive group of proteins to target is the BCL2-associated athanogene protein 1 (BAG1), a nucleotide exchange factor that binds the ATPase binding domain of the molecular chaperone HSP70/HSC70. In addition to its co-chaperone activity, BAG1 interacts with a variety of other proteins to regulate diverse cellular processes including cell division, cell death and differentiation, and transcriptional activity of the ER and androgen receptor (AR).

BAG1 is made up of different proteins translated from a single mRNA by alternative translation-initiation resulting in humans in four BAG1 isoforms (1L, 1M, 1, and 1S). These proteins have a similar carboxy-terminal BAG domain that is made up of three antiparallel alpha helical bundles of 30–40 amino acids (12) and a centrally located ubiquitin-like domain that directs HSP70 clients to the proteasome for degradation (13, 14). The largest member of the family possesses a N-terminal nuclear localization sequence and is therefore localized to the nucleus where it interacts with and regulates the activity of transcription factors including ERα and the AR (15–17). The other BAG1 proteins are cytoplasmic and they regulate an array of molecular targets within the cytoplasm that control cell proliferation, apoptosis, and stress response (18).

We have recently described a benzothiazole-based compound A4B17 with antiproliferative action in ER^+^ breast and AR-positive (AR^+^) prostate cancer cells that is reported to dock into the BAG domain of BAG1 (19, 20). This compound attenuated AR target gene expression by disrupting the interaction of BAG1 and the AR N-terminal domain that is otherwise required for transactivation by the AR. However, it required micromolar concentrations for its action. To improve on the efficacy of A4B17, we used scaffold hopping strategies to change the chemical scaffold of A4B17 and analyzed the resulting compounds for their antiproliferative action in breast and prostate cancer cells. We identified an imidazopyridine X15695 that potently inhibited proliferation of ER^+^ breast and AR^+^ prostate cancer cells. X15696 degraded ERα protein and potently inhibited ERα transactivation and in addition reactivated p53 by disrupting its interactions with members of the HSP70 chaperone family. In mouse tumor xenograft models, when administered orally, X15695 inhibited ER^+^ breast cancer cell growth more efficiently than prostate cancer cell growth. These studies together identify X15695 as an oral SERD for further development for the treatment of breast cancer.

## Materials and Methods

### Drugs and Chemicals

17-β-estradiol (Sigma-Aldrich, E8875; PubChem SID 24278426), fulvestrant (Sigma-Aldrich, I4409; PubChem SID 329815373), tamoxifen (Sigma-Aldrich, T5648), 5α-Androstan-17β-ol-3-on [Dihydrotestosterone (DHT); Merck A8380], Enzalutamide (MDV3100; Selleckchem S1250), H2DCF-DA (Thermo Fisher Scientific, D399), 4′,6-Diamidino-2-phenyllindole dihydrochloride (DAPI; Sigma-Aldrich, D9542), DRAQ5 (BioStatus Limited, DR50050), cycloheximide (Sigma-Aldrich 239763), MG132 (Calbiochem 4745790), 7-Aminoactinomycin D (7-AAD; Thermo Fisher Scientific A1310), etoposide (Sigma-Aldrich, E1383; PubChem SID 24278178), NAC (N-acetyl-L-cycteine; Santa Cruz Biotechnology, sc-202232), Hoechst stain 33,258 (Abcam, ab228550), Hoechst stain 33,342 (Invitrogen, 62249), 3-(4,5-dimethylthiazol-2-yl)-2,5-diphenyltetrazolium bromide (MTT; Sigma-Aldrich, 1.11714), 2′7-dichlorofluorescein diacetate (H2DCF-DA; Thermo Fisher Scientific D399), Lipofectamine 2000 (Thermo Fisher Scientific 11668027), Protein A-agarose beads (Thermo Fisher Scientiific, 20333), Protein G-agarose beads (Thermo Fisher Scientific 11668027), Mowiol 4-88 (Sigma-Aldrich, 81381).

### Cell Lines

All cell lines were obtained from the ATCC: MCF-7 (RRID:CVCL_0031), ZR-75-1 (RRID:CVCL_0588), MDA-MB-231 (RRID:CVCL_0062), LNCaP (CRL-1740, RRID:CVCL_0395), LAPC-4 (CRL-13009, RRID:CVCL_4744), PC3 (CL-1435, RRID:CVCL_0035), 22Rv.1 (CRL-2505, RRID:CVCL_1045), DU145 (CRL-HTB-81, RRID:CVCL_0105), HeLa (CCL-2, RRID:CVCL_0030), A549 (RRID:CVCL_0023), U2OS (RRID:CVCL_B0A7). Cells were all *Mycoplasma*-free upon receipt from ATCC prior to 2015. The identities of the cells were confirmed by short tandem repeat profiling (BioSynthesis and DSMZ). Cells were typically grown up to 20–30 passages. BAG1 short hairpin RNA (shRNA) knockdown and shRNA control LNCaP (shRNA control, 506-2, 606-2) were generated by transducing LNCaP cells transduced with predesigned BAG1 MISSION shRNA lentiviral transduction particles in pLKO.1; clones NM_004323.2-506s1c1 (clone 506) and NM_004323.2-666s1c1 (clone 666) or pLKO.1 non-silencing control (clone control C2), respectively (Mission; Sigma-Aldrich). TRMCF-7 cells are described in Morrison and colleagues, 2014 (21). All cell lines are routinely confirmed to be *Mycoplasma*-free, using the VenorGeM Classic Mycoplasma Detection Kit for conventional PCR (Minerva Biolabs, 11-1250). MCF-7, TRMCF-7, T47D, ZR-75-1, MDA-MB231, LNCaP, shRNA control and BAG1 shRNA knockout LNCaP, 22Rv.1, PC3, DU145, and LAPC-4 cells were cultured in RPMI1640 supplemented with 10% FBS, 1% penicillin/streptomycin at 37°C in an incubator with 5% CO_2_ and 90% humidity. LAPC-4 cells were additionally supplemented with 1 nmol/L DHT. HeLa, A549, and U2OS cells were cultured in DMEM supplemented with 10% FBS ,penicillin (100 U/mL), and streptomycin (100 U/mL). For experiments requiring hormone starvation, cells were cultured for 72 hours in phenol red-free RPMI1640 medium, supplemented with 3% charcoal-stripped FCS.

### p53 siRNA Knockdown and Western Blotting

Transfection experiments with siRNA were performed using Lipofectamine 2000 according to the manufacturer's instructions using SMARTpool ON-TARGETplus siRNA against p53 (Horizon Discovery, L-003329-00-0005). Briefly, 5 × 10^4^ cells were transfected with 6 μL of Lipofectamine 2000 and 10 nmol/L p53 siRNA or control siRNA (5′-GGUGCGCUCCUGGACGUAGCC-3′; Eurofins Genomic). Sixteen hours after transfection, the medium was changed and X15695 or vehicle was added to the cells for 72 hours for proliferation assay or 24 hours for protein analysis. For Western blotting, cells were lysed in NP-40 lysis buffer (1% NP-40, 50 mmol/L Tris-HCl, pH 8.0, 150 mmol/L NaCl, 5 mmol/L Ethylenediaminetetraacetic acid [EDTA]) or 1x passive lysis buffer (Promega, E1941) for 15 minutes on ice. Cell debris was removed by centrifugation at 13,000 rpm for 10 minutes and cleared lysates used for SDS-PAGE and Western blotting.

Western blotting was carried out using standard protocols with antibodies: p53 (Thermo Fisher Scientific, catalog no. MA5-14516, RRID:AB_11008717), Mdm2 (Abcam catalog no. ab-260074 RRID:AB_2936956), p53 (clone DO-1; Santa Cruz Biotechnology catalog no. sc-126, RRID:AB_628082), p53 Sp5 (Thermo Fisher Scientific, catalog no. MA1-39547, RRID:AB_2537220), BAG1 (Santa Cruz Biotechnology, catalog no. sc-33704, RRID:AB_626720), β-actin (Santa Cruz Biotechnology, catalog no. sc-47778, RRID:AB_626632), vinculin (Santa Cruz Biotechnology, catalog no. sc-25336, RRID:AB_628438), GAPDH (Santa Cruz Biotechnology, catalog no. sc-365062, RRID:AB_10847862), α-Tubulin (Santa Cruz Biotechnology, catalog no. sc-5286, RRID:AB_628411), ERα (Santa Cruz Biotechnology, catalog no. sc-8002, RRID:AB_627558), GRP75/Mortalin (Santa Cruz Biotechnology, catalog no. sc-133137, RRID:AB_2120468), AR (Santa Cruz Biotechnology, catalog no. sc-7305, RRID:AB_626671), BAG2 (Santa Cruz Biotechnology, catalog no. sc- 390107 RRID:AB_2936954), and BAG5 (Santa Cruz Biotechnology, catalog no. sc-390832 RRID:AB_2936955), Goat anti-mouse Immunoglobulins/HRP (DAKO, catalog no. P0447, RRID:AB_2617137), Goat anti-rabbit Immunoglobulins/HRP (DAKO, catalog no. P0448, RRID:AB_2617138), Goat anti-Mouse IgG (H+L) Cross-Adsorbed Secondary Antibody, Alexa Fluor 488 (Thermo Fisher Scientific, catalog no. A-11001, RRID AB_2534069), Goat anti-Rabbit IgG (H+L) Highly Cross-Adsorbed Secondary Antibody, Alexa Fluor 546 (Thermo Fisher Scientific, catalog no. A-11035, RRID AB_2534093).

### Cycloheximide Chase and MG132 Stability Assay

Unless otherwise stated, cycloheximide chase and MG132 stability experiments were performed as follows: 2 × 10^5^ MCF-7 cells were seeded in 6 cm dishes and treated with 1 μmol/L X15695 or the equivalent volume of DMSO for 24 hours. Thereafter, cells were incubated with the protein synthesis inhibitor cycloheximide (100 μg/mL) and harvested at timepoints 0, 30, 60, 90, and 120 minutes. Cells were lysed and used for Western blotting to determine the ERα level at each timepoint. For MG132 experiments, MCF-7 cells were similarly treated but in the presence or absence of the proteasome inhibitor MG132 (0.5 μmol/L) for 48 hours. Cells were harvested and lysed for Western blot assay to detect the p53 level at each condition.

### Co-immunoprecipitation

For co-immunoprecipitation experiments, 1 × 10^6^ cells were seeded in 15 cm dishes and treated with 1 μmol/L X15695 or the equivalent volume of DMSO for 48 hours. Cells were harvested, washed with PBS and lysed using NP-40 lysis buffer. Protein A agarose beads were mixed with protein G agarose beads (4:1) and preincubated overnight at 4°C with anti-p53 or anti-BAG1 or control IgG antibody. Cellular extracts from X15695 treated and untreated cells (1,000 μg) were incubated with antibody-preincubated protein A/G agarose beads for 3 to 4 hours at room temperature. The beads with immunoprecipitated proteins were collected at 4°C by centrifugation at 1,000 × *g* for 5 minutes and washed four to five times with NP-40 lysis buffer. The immunoprecipitated proteins were eluted by heating in SDS sample buffer for 5 minutes at 95°C and resolved on SDS-PAGE. Thereafter, they were subjected to Western blot analysis to detect mortalin, BAG1, BAG2, BAG5, and p53. Cellular extracts containing 50 to 70 μg protein from X15695 treated and untreated cells were used as the input control for the targeted proteins.

### RNA-sequencing and Quantitative RT-PCR Experiments

MCF-7, T47D, and LNCaP cells (5 × 10^4^ each) were seeded in 6-well plates and cultured with hormone-depleted medium for 3 days. Subsequently, the MCF-7 and T47D cells were treated with 10 nmol/L 17-β-estradiol (E_2_) or DHT for the LNCaP cells, 1 μmol/L X15695 (for the MCF-7 and T47D cells) or 5 μmol/L for LNCaP cells and X15695 together with E_2_ or DHT. A total of 1 hour pretreatment with X15695 was performed prior to treatment with E_2_ or DHT for 16 hours. RNA was extracted using innuPREP RNA mini Kit 2.0 (Analytic Jena, 845-KS-2040250). A total of 1 μg RNA per sample was used as input material for the RNA sample preparations. Sequencing libraries were generated using NEBNext UltraTM RNA Library Prep Kit for Illumina (New England Biolabs, E7530L) following the manufacturer's recommendations and index codes were added to attribute sequences to each sample. The clustering of the index-coded samples was performed on a cBot Cluster Generation System using PE Cluster Kit cBot-HS (Illumina) according to the manufacturer's instructions. After cluster generation, the library preparations were sequenced on an Illumina platform and paired-end reads were generated. Library preparation and sequencing were carried out by Novogene Europe. The RNA-sequencing (RNA-seq) data were analyzed by R (v4.1.1, RRID:SCR_001905). Differential expression analysis was performed using R package Limma (v3.52.2, RRID:SCR_010943) and visualized with R package ggplot2 (v 3.4.2, RRID:SCR_014601) with absolute values of log_2_ fold changes ≥1 and p(FDR) ≤0.05. Gene Ontology analysis and gene set enrichment analysis (GSEA; RRID:SCR_006484) was performed using R package GSEABase (v1.58.0) and GSVA (v1.44.2) and the results were visualized by R package enrichplot (v1.16.1; refs. 22, 23). The data were deposited at the Gene Expression Omnibus repository under accession number GSE218556. Select target genes were analyzed by RT-PCR experiments with RNA from the samples used for the RNA-seq analysis. PCR analysis was carried out using the SYBR Green GoTaq PCR mix (Promega, A6002) and Rib36B4 as a housekeeping gene. The sequence of the primers used is in the Supplementary Data.

#### MTT Cell Viability Assay

Cells were seeded in duplicates at a density of 1 × 10^4^ cells/well in a 24-well plate format and incubated for 6 days in culture medium. Cells transfected with siRNA were incubated for 72 hours after X15695 addition. Thereafter, the medium was exchanged for a fresh medium containing 0.5 mg/mL MTT and incubated at 37°C for 2 hours until intracellular purple formazan crystals were formed. The MTT solution was removed and the purple crystals were dissolved in isopropanol and the optical absorbance at 590 nm was recorded using SpectraMax iD3, Molecular Devices.

### Clonogenic Assay

Cells were seeded at a density of 1–2 × 10^3^ cells/well in a 6-well plate and treated with increasing concentrations of X15695 and cultured for 14–21 days. Medium and compounds were exchanged after 7 days. Cells were fixed with methanol/acetic acid mixture [3:1 volume for volume (v/v)] and the formation of colonies was visualized using 0.5% crystal violet (w/v) in 20% methanol (v/v). Plates were scanned in a conventional office scanner (Epson). The area covered by colonies was calculated using the ColonyArea Plugin for ImageJ (RRID:SCR_003070; ref. 24).

### Reduced H2DCF-DA Oxidation Assay

MCF-7 and T47D cells were seeded at 1 × 10^3^ cells and treated with 10^−9^ to 10^−6^ mol/L X15695 for 48 hours. Afterward, cells were loaded with 10 μmol/L H2DCF-DA in phenol red-free medium for 40 minutes. Cells were subsequently washed with medium and further incubated in medium for 120 minutes before analysis of fluorescence at 485 nm excitation and 530 nm emission using a multi-well fluorescence reader (SpectraMax iD3, Molecular Devices with SoftMax Pro 7 software). After the DCF assay, cells were frozen at −80°C overnight. After thawing, DNA was stained with a Hoechst dye 33258 (10 μg/mL final concentration) for 30 minutes at room temperature and fluorescence was measured at 352 nm excitation and 454 nm emission in a multi-well fluorescence reader (SpectraMax iD3, Molecular Devices with SoftMax Pro 7 software). DCF fluorescent intensities were normalized to Hoechst fluorescent intensities.

#### Immunofluorescence Assay

For immunofluorescence assay, MCF-7, T47D, 22Rv.1, LAPC-4, and LNCaP cells (1 × 10^4^ each) were seeded on coverslips in a 24-well plate and the desired treatment applied. After three times washing with PBS, cells were fixed with 4% paraformaldehyde solution for 10 minutes at room temperature. Next, the cells were washed three times with PBS and permeabilized with 0.5% Triton X-100 for 10 minutes at room temperature. Following three additional wash steps with PBS, cells were treated with 5% BSA (w/v) for 1 hour at room temperature and subjected to overnight incubation with the primary antibody p53 (DO1 clone or Sp5), ERα or mortalin/GRP75 and then washed three times with PBS followed by the incubation with goat anti-mouse IgG, Alexa Fluor 488 antibody and/or goat anti-rabbit IgG, Alexa Fluor 546 antibody for 1 hour at room temperature in the dark. All subsequent steps were performed in the dark. Cells were washed three times with PBS and stained with 0.1 μg/mL DAPI solution at 1:10,000 (v/v) dilution for 15 minutes at room temperature. After three times washing with PBS, the cells were mounted in Mowiol 4-88 on microscope slides and observed by laser-scanning confocal microscopy (Confocal Microscope platform STELLARIS6-LSM900, Leica).

### Flow Cytometry Assay

LNCaP, LAPC-4, MCF-7, and T47D cells were treated with 1 μmol/L X15695 or DMSO for 48 hours. Single-cell suspensions (1 × 10^6^ cells) were prepared in 150 μL PBS/10% FBS. Cells were fixed by addition of 5 mL ice-cold ethanol (70%) and incubated overnight at 4°C. Fixed LNCaP and LAPC-4 cells were stained with 20 μmol/L DRAQ5 (5 mmol/L stock, BioStatus DR050050) and incubated for 15 minutes at room temperature. Fixed MCF-7 and T47D cells were resuspended in PBS at a concentration of 1 × 10^6^ cells/100 μL and stained by addition of 5 μL of 7-AAD (Thermo Fisher Scientific A1310) for 15 minutes at room temperature in the dark. Flow cytometry analysis was performed using BD FACSAria Fusion (BD Biosciences). Doublets and dead cells were excluded from the analysis. Flow cytometry data were analyzed using FlowJo 10.8.1 cytometry analysis software (FlowJo, LLC; RRID:SCR_008520).

Induction of apoptosis in response to treatment with X15695 was analyzed in MCF-7 and T47D cells using PE Annexin V Apoptosis Detection Kit with 7-AAD (BD Biosciences, 556421) according to the manufacturers protocol. In short, cells were harvested by trypsinization and single-cell suspensions (1 × 10^6^ cells) were prepared as described above in 100 μL PBS. Cells were stained with 5 μL PE Annexin V and 5 μL 7-AAD for 15 minutes at room temperature in the dark. Subsequently, 400 μL PBS were added to each sample and flow cytometry measurements were performed within 1 hour. Cells stained with PE Annexin V or 7-AAD alone were used for compensation. Flow cytometry analysis was performed using BD FACSAria Fusion. Flow cytometry data were analyzed using FlowJo 10.8.1 cytometry analysis software (FlowJo, LLC).

Apoptosis in LNCaP and LAPC-4 cells was measured using an automated high-throughput microscopy method as previously described under HT/C assays at KIT (25). In brief, LNCaP and LAPC4 cells (5 × 10^3^) were seeded in a 96-well plate format. Cells were treated with 10^−9^ to 10^−6^ mol/L X15695, DMSO or etoposide (250 μmol/L) as control for 48 hours. Cells were stained with Hoechst 33342 (0.15 μg/mL) and propidium iodide (PI; 0.25 μg/mL) for 30 minutes at 37°C. Four brightfield and fluorescence images were acquired from different positions in each well using an automated Olympus IX81 fluorescence microscope (Olympus). The Hoechst dye was detected at excitation 350 nm and emission 450 nm and PI dye was detected at 488 and 590 nm. The total number of cells (all Hoechst-stained nuclei) as well as the number of early apoptotic, late apoptotic, and necrotic cells (combination of Hoechst and PI nuclei intensity) was obtained by analyzing images with the scanˆR software (version 2.7.3, Olympus).

### Mouse Xenograft Experiments

All animal experiments were performed according to European and German statutory regulations and approved by the Regierungspräsidium Karlsruhe, Germany.

MCF-7 and LAPC-4 cells (2 × 10^6^) were suspended in 100 μL PBS: Matrigel (Corning, 354248; 1:1) and injected subcutaneously into both flanks of 6–7 weeks old female (for MCF-7 cells) and male (for LAPC-4 cells) athymic nude-*Foxn1^nu^* mice (Envigo 6901M). When tumor volumes were around 50–100 mm^3^, mice were randomized into four groups (7 mice per group) and treated daily by oral gavage as follows: (i) Control group (Vehicle; corn oil, 1.0% DMSO) and (ii) X15695 group, 30 mg per kg body weight) for the MCF-7 tumors or (i) Control group (Vehicle; corn oil, 1.0% DMSO), (ii) enzalutamide (Enza group, 15 mg per kg body weight), (iii) X15695 group (30 mg per kg body weight), and (iv) X15695 group (15 mg per kg body weight). Body weight and tumor volumes were measured twice a week. Tumor volume was measured with a digital vernier caliper and calculated using the formula vol = length × width × height × 0.5236 and recorded or calculated relative to the starting volume which was nominally set at 100%. After 16 days for the MCF-7 tumors or 42 days for the LAPC-4 tumors, mice were sacrificed and the tumors removed, photographed, and weighed. Percent body weight change was calculated using the following formula: group percent weight change = [(new weight − initial weight)/initial weight] × 100. Tumors were snap-frozen and stored for Western blot analysis.

### Synthesis of Imidazopyridines (See Supplementary Data)

#### Graphing and Statistical Analysis

Experiments were performed with three or more replicates. Differences between two groups were analyzed by Student *t* test and multiple comparisons were determined by one-way ANOVA. If there were two factors (such as dose and time) investigated, data were analyzed by two-way ANOVA followed by a *post hoc* test. Data were expressed as means ± SEM, and *P* < 0.05 was considered significant. All analyses were performed using Microsoft Excel 2010 (RRID:SCR_016137) and GraphPad Prism 8.3.1 (RRID:SCR_002798) software.

### Data Availability Statement

All data relevant to the study are included in the article or Supplementary Data. The data that support the Chemistry part of this study are available in the repository Chemotion (https://www.chemotion-repository.net/). All DOIs minted for the data are linked to the specific experiments in the supporting information and a summary of all new data obtained in this study can be accessed with the collection: DOI https://dx.doi.org/10.14272/collection/SGV_2022–09–29. Crystallographic data for compounds X15695, X15696 and X19168 reported in this article have been deposited with the Cambridge Crystallographic Data Centre as supplementary information no. CCDC- 2226035, 2218538, and 2218539. Copies of the data can be obtained free of charge from https://www.ccdc.cam.ac.uk/structures/. The compounds produced in this study can be requested from the Molecule Archive of KIT either upon request according to contact details given in the Supplementary Data or following the information available in Chemotion repository (https://dx.doi.org/10.14272/collection/SGV_2022–09–29).

## Results

### Growth Inhibitory Properties of Imidazopyridine Derivatives

**FIGURE 1:**
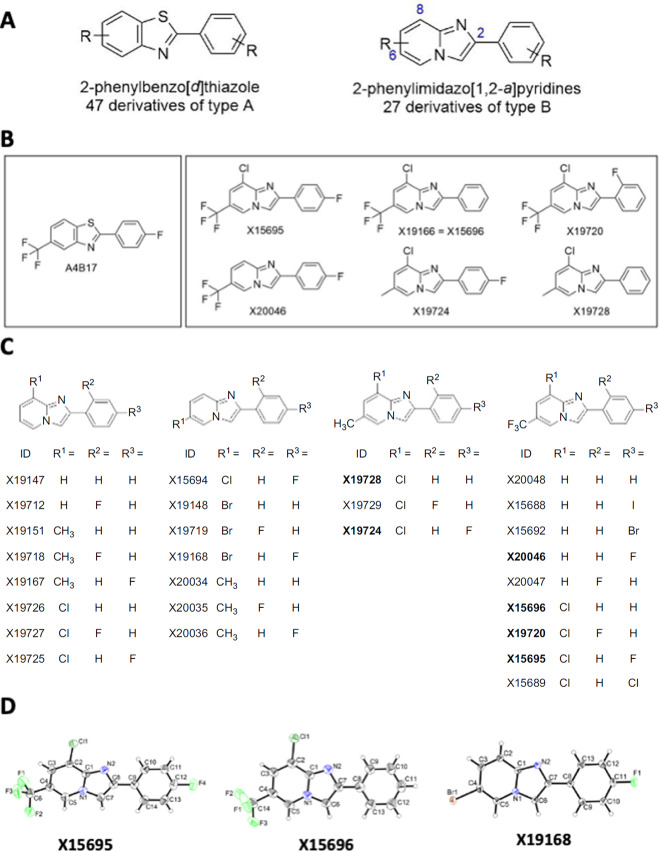
Imidazopyridine structures for inhibition of prostate and breast cancer proliferation. **A,** Comparison of benzothiazole and imidazopyridine scaffolds. **B,** Chemical structures of the benzothiazole A4B17 and six imidazopyridines that potently inhibit clonal expansion of ER^+^ and AR^+^ breast and prostate cancer cells. **C,** Chemical structures showing the structure of different imidazopyridine derivatives. **D,** Crystal structures of X15695, X15696, and X19168.

We have previously reported that the compound A4B17 derived from a screen of 47 benzothiazoles inhibited proliferation of ER^+^ breast and AR^+^ prostate cancer cells with half maximal inhibitory concentrations (IC_50_) in the micromolar range (19, 20). To improve on the potency of this compound, we used a scaffold hopping approach focusing on heterocycle replacement (26) to change the benzothiazole into an imidazopyridine (from core of type A to core of type B; Fig. 1A) to generate X20046 from A4B17 (Fig. 1B). This greatly improved the inhibitory properties of X20046 both in breast and prostate cancer cells (Supplementary Table S1).

We therefore synthesized a series of 27 imidazopyridines (Fig. 1C) based on the core structure of type B as given in Fig. 1A (top right), determined the X-ray structure of X15695, X15696, and X19168 (Fig. 1D; Supplementary Table S2) for rational drug design and analyzed the ability of all 27 compounds to inhibit the clonal expansion of a series of breast and prostate cancer cells. All the compounds outperformed A4B17 in the inhibition of clonal expansion of ER^+^ breast and AR^+^ prostate cancer cells and had very weak or no effect on receptor-negative cell lines (Supplementary Table S1). With only few exceptions, compounds that potently inhibited colony formation of ER^+^ cells also potently inhibited the clonal expansion of the AR^+^ prostate cancer cell lines. Six imidazopyridines, including X20046 (the imidazopyridine derivative of A4B17) were identified as particularly potent based on their IC_50_ values in the two different cell types (Supplementary Table S1). These compounds share a striking resemblance in their core structures (Fig. 1B) and show a requirement for either a methyl group or a CF3 group at position C-6 of the imidazopyridine core similar to other derivatives that were only active against one of the two cancer cell types analyzed. From the six identified compounds, X15695 was chosen for further study.

### Transcriptomics Analyses to Determine X15695 Action

To determine the mechanism of action of X15695, we first performed transcriptomics analyses in the ER^+^ breast cancer cell lines MCF-7 and T47D after treating them with vehicle (DMSO), 17-β-estradiol (E_2_), X15695, and a combination of E_2_ and X15695. The RNA-seq datasets were analyzed using a fold change of log_2_(counts per million [CPM]) ≥ 1.0 or ≤ −1.0 and a *P*_adj_ value ≤ 0.05 to identify differentially expressed genes (DEG) in response to X15695 in the presence or absence of E_2_. A total of 531 DEGs (327 DEGs downregulated and 204 DEGs upregulated) was identified in MCF-7 cells in response to X15695 in the absence of E_2_ but 439 DEGs (238 DEGs downregulated and 201 DEGs upregulated) in response to X15695 in the presence of E_2_ (Supplementary Fig. S1A and S1B). In the T47D cells, 487 DEGs (302 downregulated and 185 upregulated) were identified in response to X15695 in the absence of E_2_ treatment while in the presence of E_2_, slightly less DEGs were seen (458; 280 downregulated and 176 genes upregulated; Supplementary Fig. S1C and S1D).

**FIGURE 2:**
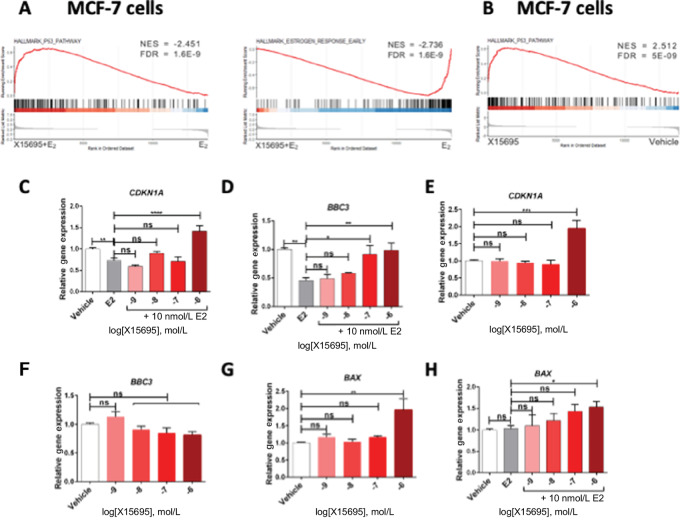
X15695 attenuates ERα-mediated target gene expression. GSEA plots of the top gene sets identified in a comparison of E_2_ + X15695 versus E_2_ (**A**) and X15695 versus vehicle (**B**) in MCF-7. **C–H,** Quantitative RT-PCR to detect the effect of X15695 on the expression of the indicated p53 target genes in MCF-7 cells in the presence and absence of E_2_. Cells were treated with the indicated concentrations of X15695 in the presence and absence of 10 nmol/L E_2_ for 16 hours. The data represent the mean ± SEM (*n* = 4; *, *P* ≤ 0.05; **, *P* ≤ 0.01; ***, *P* ≤ 0.001; ****, *P* ≤ 0.0001; ns, not significant).

To investigate the pathways associated with the X15695-mediated changes in gene expression, GSEA was performed using the Hallmarks gene sets from the Molecular Signatures Database (MSigDB, RRID:SCR_016863; ref. 27). Two of the most regulated pathways by X15695 in the presence of E_2_ in MCF-7 cells were the ERα and p53 signaling pathways. In the absence of E_2_, the p53 signaling pathway was also identified as the most regulated pathway (Fig. 2A and B; Supplementary Fig. S1A and S1B, right). Among other signatures, the ERα and the p53 pathways were also the most significantly regulated pathways by X15695 in the T47D cells (Supplementary Fig. S1C and S1D, right). Therefore, a heat map was generated to compare E_2_ response genes in MCF-7 and T47D cells and this showed a very strong overlap in the downregulation of expression of ERα target genes in the two cell lines (Supplementary Fig. S2A). Quantitative real-time polymerase chain reaction (RT-PCR) carried out with a select number of ERα response genes in the two cells showed indeed downregulation of E_2_-mediated gene expression by X15695 (Supplementary Fig. S2B and S2C). On the contrary, the p53 pathway was upregulated by X15695 in the two cell lines as shown in the heat map but more strongly in the MCF-7 cells compared with the T47D cells (Supplementary Fig. S2D). X15695-mediated upregulation of expression of p53 target genes in the absence and presence of E_2_ was also confirmed in the qRT-PCR analyses, but the results in the MCF-7 cells were somewhat more prominent than in the T47D cells (Supplementary Fig. S2E and S2F).

We detected that X15695 did not only potently inhibit E_2_-induced gene transcription but simultaneously increased the expression of genes negatively regulated by E_2_, including genes involved in cell-cycle arrest and in the induction of apoptosis. For example, E_2_-mediated downregulation of expression of *CDKN1A* and *BBC3* in the MCF-7 cells was dose-dependently upregulated by X15695 (Fig. 2C and D). In addition, the expression of *CDKN1A* but not *BBC3* was upregulated by X15695 in the absence of E_2_ (Fig. 2E and F), making *CDKN1A* a gene under dual control mechanisms by E_2_ and X15695. In contrast, the expression of the p53 downstream target gene *BAX*, was not regulated by E_2_ but was transcriptionally enhanced by X15695 (Fig. 2G and H). These findings identified X15695 as a compound that targets both the ERα and p53 signaling pathways and can therefore control the actions of the p53-ER regulatory loop described in breast cancers (28).

### Mechanism of Downregulation of ERα and Upregulation of p53

**FIGURE 3:**
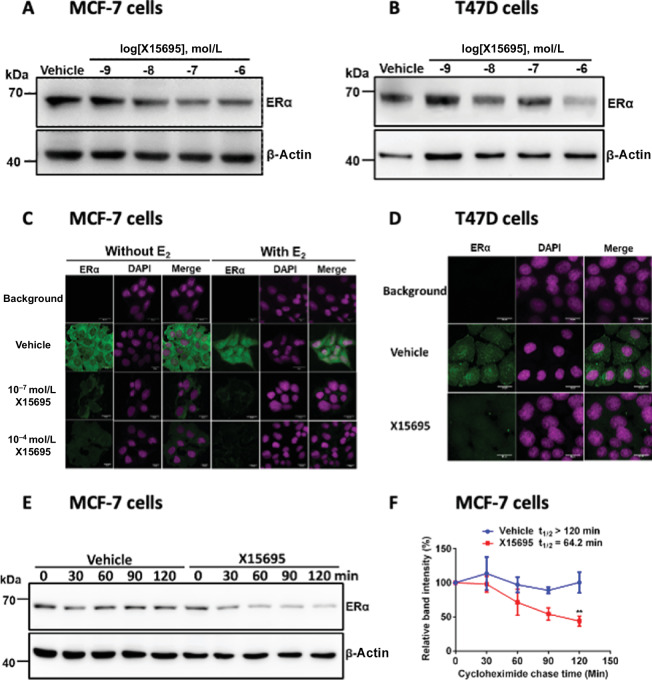
X15695 destabilizes ERα. Western blot analysis of ERα in extracts of MCF-7 (**A**) and T47D (**B**) cells after treatment with vehicle or the indicated concentrations of X15695 for 48 hours. Anti-β-actin antibody was used for protein loading control. The protein samples used to determine the expression level of ERα were also used for the determination of p53 levels in Fig. 4A and B. Therefore, the β-actin control levels in these experiments are identical. Representative immunofluorescent staining carried out on hormone starved MCF-7 cells (**C**) treated with 100 nmol/L or 1 μmol/L X15695 in the presence and absence of 10 nmol/L E_2_ for 16 hours and T47D cells (**D**) treated with 1 μmol/L X15695 for 16 hours in full medium without hormone depletion. Staining was performed with anti-ERα antibody followed by goat anti-mouse IgG, Alexa Fluor 488 (green) and by DAPI (magenta). Fluorescence images were taken at 20×3 magnification. **E,** Western blot analysis of ERα in extracts of MCF-7 cells treated with vehicle or X15695 (1 μmol/L) for 24 hours followed by treatment with 100 μg/mL cycloheximide. The reaction was termination at the indicated timepoints. Anti-β-actin antibody was used to determine equal protein loading. **F,** Protein signals were quantified and presented in percentages as the signal of ERα relative to the β-actin signal. The value at timepoint zero of cycloheximide treatment was set to 100%. The values are the means ± SEM, *n* = 3; **, *P* ≤ 0.01.

We sought to investigate the possible mechanisms of X15695-mediated regulation of ERα and p53 signaling pathways in the breast cancer cells. In immunoblotting studies, X15695 dose-dependently decreased ERα level in both cell lines (Fig. 3A and B). In the MCF-7 cells, ERα staining in the absence and presence of E_2_ was strongly reduced by X15695 treatment (100 nmol/L and 1 μmol/L) in the cytoplasm and in the nucleus (Fig. 3C). A similar downregulation in ERα level was observed in immunofluorescence experiments in T47D cells (Fig. 3D), albeit in full medium without additional hormone.

To determine how X15695 downregulates ERα level, MCF-7 cells were treated with cycloheximide to inhibit *de novo* protein synthesis and the degradation kinetics of the steady state population of the receptor was determined. This showed that while ERα was relatively stable (>120 minutes) in the absence of X15695, its half-life was reduced to about 60 minutes in the presence of X15695 (Fig. 3E and F), suggesting destabilization of ERα by X15695.

**FIGURE 4:**
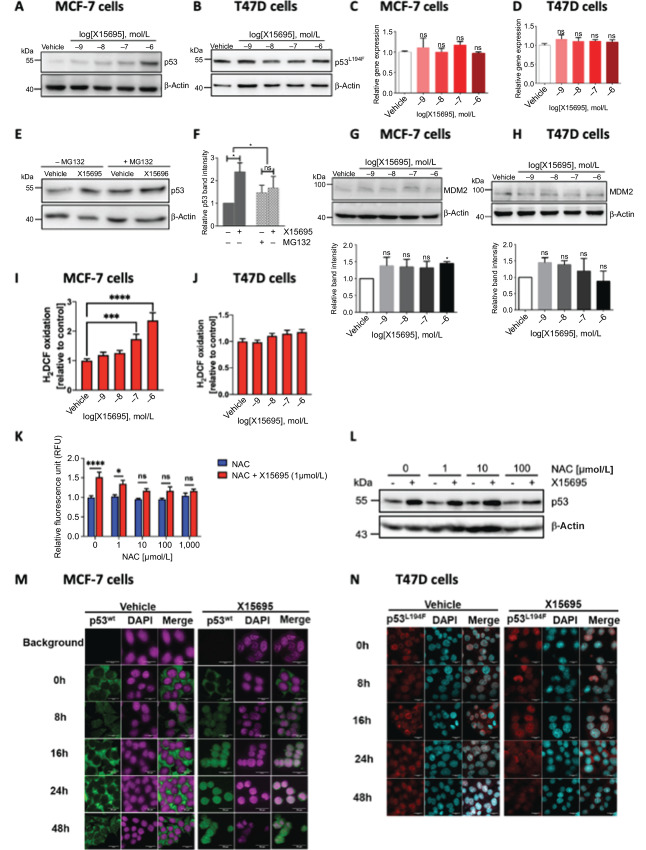
X15695 stabilizes p53. Western blot analysis of p53 in extracts of MCF-7 (**A**) and T47D (**B**) cells treated with vehicle or the indicated concentrations of X15695 for 48 hours. Anti-β-actin antibody was used for the loading control (same as it appears in Fig. 3A and B). Quantitative RT-PCR of p53 expression in MCF-7 (**C**) and T47D (**D**) cells after treatment with vehicle (DMSO) or the indicated concentrations of X15695 for 48 hours. The results are the mean ± SEM (*n* = 3; ns, nonsignificant). **E,** Western blot analysis of p53 in extracts of MCF-7 cells treated with 0.5 μmol/L MG132 in the presence and absence of 1 μmol/L X15695 for 48 hours. Anti-β-actin antibody was used for the loading control. **F,** Quantification of the p53 protein band intensities relative to the intensity of the β-actin signal. The results are the means ± SEM (*n* = 3; *, *P* ≤ 0.05; ns, nonsignificant result). Western blot analysis of MDM2 levels in extracts of MCF-7 (**G**) and T47D (**H**) cells after treatment with the indicated concentration of X15695 for 48 hours. Anti-β-actin antibody was used for determination of equal protein level. The protein band intensities were quantified and presented below. Measurement of DCF fluorescence in MCF-7 (**I**) and T47D (**J**) cells treated with the indicated concentrations of X15695 or DMSO for 48 hours. The signals were normalized to DNA content of the samples measured after Hoechst 33258 staining. The results are the mean ± SEM (*n* = 3; ***, *P* ≤ 0.001; ****, *P* ≤ 0.0001). **K,** Reduced H2DCF-DA oxidation assay showing the effect of NAC on ROS production in MCF-7 cells treated with X15695 for 48 hours. Results represent the mean H2DCF-DA oxidation normalized to DNA content (*n* = 1 with 6 technical replicates). **L,** Western blots of lysates of MCF-7 cells treated with the indicated concentrations of NAC 1 hour prior to treatment with 1 μmol/L X15695 or DMSO for 48 hours. Expression of p53 was monitored using anti-p53 antibody and anti-β-actin antibody was used as a protein loading control. **M** and **N,** Immunofluorescence images showing p53 localization in MCF-7 and T47D cells treated with vehicle (DMSO) and 1 μmol/L X15695 for the indicated time periods. For staining, anti-p53 antibody was used followed by goat anti-mouse IgG, Alexa Fluor 488 antibody – green) and DAPI (magenta; M) or Alexa Fluor 488 antibody – red) and DAPI (light blue; N).

In contrast to the decrease in ERα levels in both MCF-7 and T47D cells, X15695 differentially regulated the level of p53 in the two cell lines. In Western blot experiments, p53 level was significantly increased by X15695 in MCF-7 but not in T47D cells (Fig. 4A and B). Note that MCF-7 cells express a wild-type p53 while T47D cells express a mutated p53 (L194F) which may account for the difference in regulation by X15695. The upregulation of p53 expression in the MCF-7 cells did not occur at the RNA level as demonstrated in qRT-PCR experiments (Fig. 4C and D), suggesting that the regulation occurred at the protein level. As protein turnover is a dynamic process controlled by the rate of protein synthesis and degradation, inhibition of protein degradation should provide information on the contribution of the latter process to the accumulation of p53. X15695 treatment in the absence of the proteasomal inhibitor MG132 led to an accumulation of p53 in the MCF-7 cells but in the presence of MG132 (Fig. 4E), the basal level of p53 was increased and no further upregulation by X15695 was observed. Quantification of the effect of MG132 and X15695 on p53 level presented in Fig. 4F showed that X15695 functions by inhibiting proteasomal degradation of p53. In T47D cells where the proteasomal degradation pathway is reportedly nonfunctional (29, 30), mutant p53^L194F^ accumulated in the non-treated cells and its level was unaltered by X15695 administration (Fig. 4B).

To further understand the mechanism leading to p53 accumulation, we analyzed the expression level of MDM2, an E3 ubiquitin ligase that directs p53 degradation by the proteasome machinery (31, 32). Immunoblot analysis showed that in MCF-7 cells, X15695 slightly upregulated MDM2 protein level (Fig. 4G) rather than downregulating it to account of the negative effect of X15695 on p53 degradation. Therefore, X15695’s action on p53 in MCF-7 cells is independent of MDM2. In T47D cells, neither MDM2 nor p53 level was affected by X15695 treatment (Fig. 4H). An alternative pathway for the upregulation of p53 level is through reactive oxygen species (ROS; ref. 33). This pathway is among the top processes identified in the GSEA plots of transcripts from both MCF-7 and T47D cells (Supplementary Fig. S1). Besides, increased ROS generation has been reported following BAG1 knockout in MCF-7 cells (34). We therefore measured ROS production in MCF-7 and T47D cells after X15695 treatment using the fluorescent probe H2DCF-DA and showed a significant dose-dependent ROS production in MCF-7 but not in T47D cells (Fig. 4I and J). ROS production in MCF-7 cells possibly contributed in part to the upregulation of p53, as the ROS scavenger NAC at 100 μmol/L inhibited both the X15695-mediated increase in ROS production and upregulation of p53 level (Fig. 4K and L).

In addition to increasing p53 expression level, X15695 also regulated the cellular localization of this protein. In immunofluorescence studies in MCF-7 cells, p53 was found mainly in the cytoplasm but accumulated in the nuclear compartment as early as 8 hours after X15695 treatment (Fig. 4M). In T47D cells, p53 was already nuclear in the absence of X15695 in agreement with published information (35) and its cellular localization was not further altered by X15695 treatment (Fig. 4N).

### X15695 Disrupts p53–Mortalin Interaction

The cytoplasmic localization of wild-type p53 in tumor cells is reported to be due to sequestration by GRP75 (a.k.a mortalin), a member of the HSP70 molecular chaperone family (36, 37). In immunofluorescence experiments in MCF-7 cells, we confirmed the cytoplasmic colocalization of p53 and mortalin but after X15695 treatment, p53 was translocated to the nucleus while mortalin assumed a perinuclear localization (Supplementary Fig. S3A). In contrast, p53^L194F^ was nuclear in T47D cells and its cellular localization was not altered by X15695 treatment (Supplementary Fig. S3B).

**FIGURE 5:**
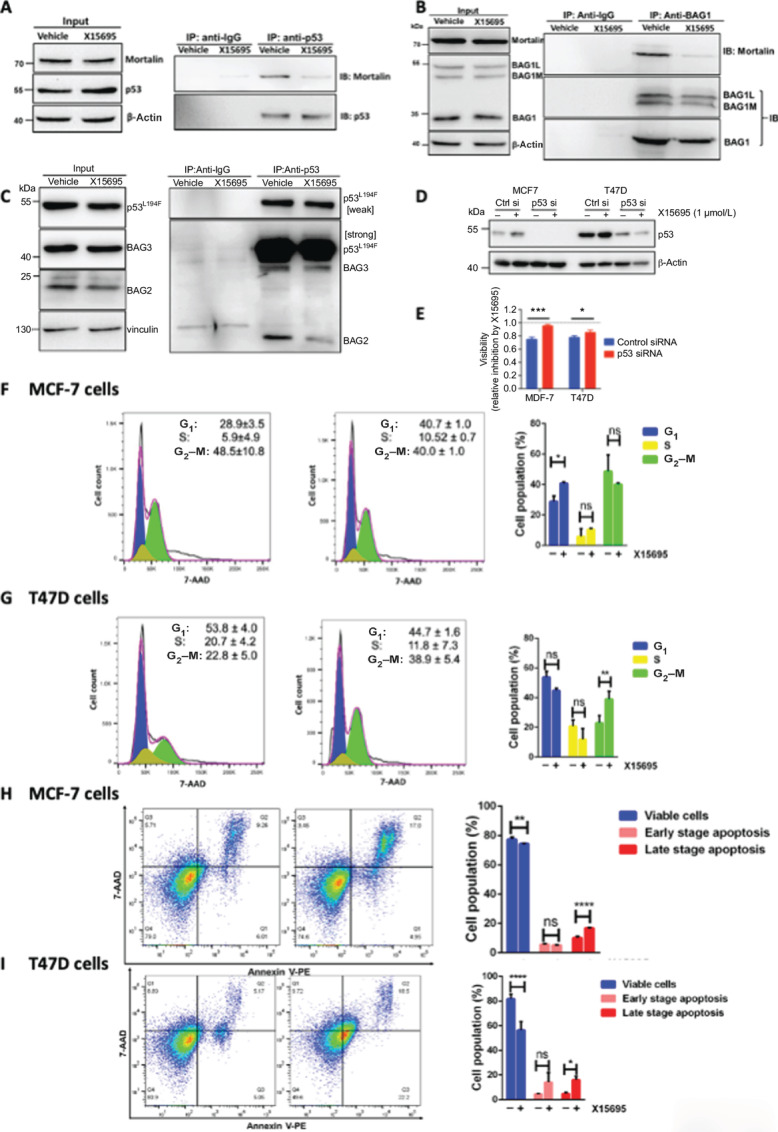
X15695 reactivates p53. **A,** Western blot analysis of mortalin and p53 in immunoprecipitated extracts of MCF-7 cells treated with 1 μmol/L X15695 for 48 hours and lysed. 50 μg of cell extract was used as input control while 1,000 μg was immunoprecipitated with anti-p53 or IgG antibodies immobilized on protein A/G agarose beads. **B,** Western blot analysis of mortalin and BAG1 in immunoprecipitated extracts of MCF-7 cells treated with 1 μmol/L X15695 for 48 hours. MCF-7 cells were treated and processed as in A with the exception that the immunoprecipitation was carried out with a mouse anti-BAG1 antibody. **C,** Western blot analysis of BAG2 and BAG5 in immunoprecipitated extracts of T47D cells treated with 1 μmol/L X15695 for 48 hours. A total of 70 μg of cell extracts was used as input control while 1,000 μg was immunoprecipitated with anti-p53 or IgG antibodies immobilized on protein A/G agarose beads. **D,** Western blot analysis of p53 protein after transfection of MCF-7 and T47D cells with control and p53 siRNA and treatment with vehicle or X15695 (1 μmol/L) for 24 hours. β-actin was used for the loading control. **E,** Cell viability determination using MTT assay in control and p53 siRNA transfected cells treated with vehicle or X15695 (1 μmol/L) for 72 hours (*n* ≥ 3; ***, *P* ≤ 0.001; *, *P* ≤ 0.05). Cell-cycle analysis of MCF-7 (**F**) and T47D (**G**) cells treated with vehicle or 1 μmol/L X15695 for 48 hours. A total of 1 × 10^6^ cells were stained with 7-AAD and used for flow cytometry measurement. Data were analyzed by FlowJo software. Apoptosis assessment of MCF-7 (**H**) and T47D (**I**) cells treated with 1 μmol/L X15695 for 48 hours. The cells were double stained with 7-AAD and Annexin V. Cells stained with 7-AAD or Annexin V alone were used for compensation of double staining. A total of 1 × 10^6^ cells were used for the flow cytometry measurement. The quantified results are the mean ± SEM (*n* = 3; *, *P* ≤ 0.05; **, *P* ≤ 0.01; ***, *P* ≤ 0.0001; ns is non-significant).

To account for the alteration in cellular localization of p53 in MCF-7 cells, we showed in co-immunoprecipitation experiments that in the absence of X15695, p53 was in a complex with mortalin but this interaction was decreased by X15695 treatment (Fig. 5A). Mortalin itself is reported to interact with BAG1 (38) and we showed that the interaction of all three isoforms of BAG1 with mortalin was also disrupted by X15695 treatment (Fig. 5B). In T47D cells that express a mutant p53, tumorigenesis is reported to proceed through a gain-of-function (GOF) mechanism involving nuclear interaction of mutant p53 with BAG2 and BAG5 (39, 40). In co-immunoprecipitation experiments, we also showed that X15695 disrupted the interaction of BAG2 but not BAG5 with mutant p53 in the T47D cells (Fig. 5C), pointing to a possible action of X15695 in the disruption of BAG/p53 complexes in breast cancer cells.

To further confirm a role of p53 in X15695 action, we transfected MCF-7 and T47D cells with control and a mixture of siRNAs against p53 and analyzed the inhibitory effect of X15695 on cell survival in the p53 knockout cells. If p53 were to play a role in the action of X15695, the antiproliferation action of this compound is expected to be attenuated by the knockdown of p53. We showed a near complete knockdown of p53 in MCF-7 cells but not in the T47D cells (Fig. 5D). As a consequence, decreased cell survival mediated by X15695 was significantly attenuated in the p53 siRNA-transfected MCF-7 cells but this effect was somewhat compromised in the p53 knockdown T47D cells (Fig. 5E). These studies demonstrated the involvement of p53 in the inhibitory action of X15695.

### X15695 Regulates Cell-cycle Progression and Apoptosis

One of the functions of p53 in the nucleus is to regulate the expression of downstream targets such as p21, GADD45A, PUMA, and BAX that control cell-cycle progression and apoptosis (28). As genes encoding these proteins are significantly activated by X15695 in the breast cancer cells (Supplementary Fig. S2E and S2F), X15695 would be expected to induce cell-cycle arrest and apoptosis. Flow cytometry experiments carried out with 7-AAD after treating MCF-7 and T47D cells with X15695 showed a G_1_–S-phase arrest in MCF-7 cells (Fig. 5F) while a G_2_–M cell-cycle arrest was observed in the T47D cells (Fig. 5G). Double staining with 7-AAD and Annexin V used for apoptosis evaluation revealed increased number of the cells at the late stages of apoptosis upon X15695 treatment in both MCF-7 and T47D cells (Fig. 5H and I).

### X15695 Regulation of Tamoxifen-resistant MCF-7 Cells

**FIGURE 6:**
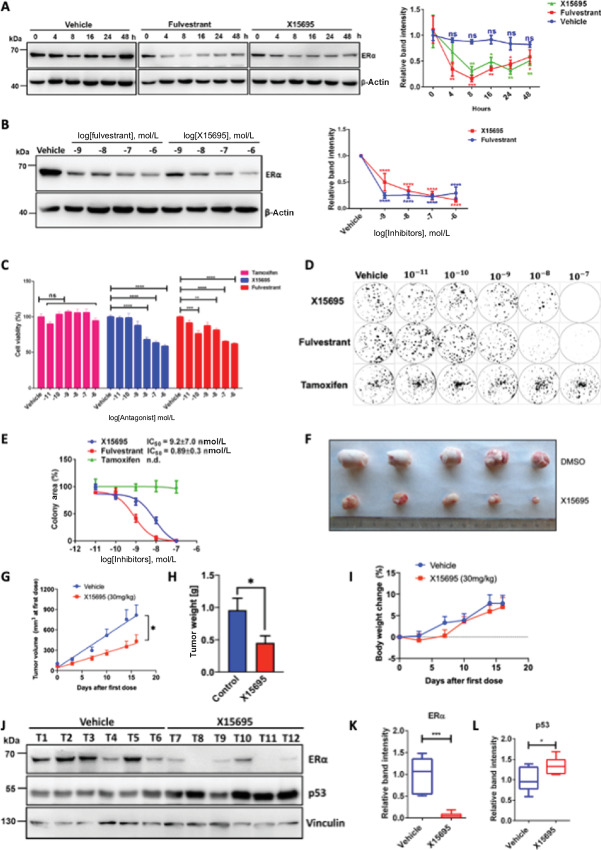
X15695 and fulvestrant downregulate ERα level and proliferation of breast cancer cell proliferation **A,** Western blot analysis of ERα in extracts of MCF-7 cells treated for the indicated timepoints with 1 μmol/L of fulvestrant or X15695 or DMSO (vehicle). β-actin was used for the loading control. The protein signals were quantified and presented in percentages as the signal of ERα relative to the β-actin signal (right). The value at zero time of treatment was set to 100%. The values are the mean ± SEM (*n* = 3; *, *P* ≤ 0.05; **, *P* ≤ 0.01; ***, *P* ≤ 0.001). **B,** Western blot analysis of ERα in extracts of MCF-7 cells treated with the indicated concentration of fulvestrant or X15695 for 8 hours. β-actin was used for the loading control. The protein signals were quantified and presented in percentages relative to the vehicle-treated sample (right). The values are the mean ± SEM (*n* = 3; ****, *P* ≤ 0.0001). **C,** Measurement of cell viability by MTT assay of tamoxifen resistant cells (TRMCF-7) treated with the indicated concentrations of X15695, fulvestrant, and tamoxifen for 48 hours. Five replicates were used for each experiment, and the experiment was repeated three times (**, *P* ≤ 0.01; ***, *P* ≤ 0.001; ****, *P* ≤ 0.0001; ns, nonsignificant). **D,** Representative image of a clonogenic assay showing the effect of the indicated concentrations of X15695, fulvestrant, and tamoxifen on clonal expansion of tamoxifen-resistant MCF-7 cells. **E,** Quantification of colonies in the clonogenic assay described in D. **F,** Representative images of some of the tumors obtained in a mouse MCF-7 tumor xenograft after treatment with vehicle and X15695. **G,** Tumor volumes of established subcutaneous MCF-7 xenografts treated daily via oral gavage with vehicle or X15695 (30 mg/kg body weight). The values are the mean ± SEM (*, *P* = 0.05; *n* = 14). **H,** Tumor weights at the end of treatment were recorded and presented in the bar chart. The values are the means ± SEM (*, *P* = 0.05; *n* = 14). **I,** Mouse body weights measured twice weekly for all animals over 16 days. Presented are the mouse body weight changes, in percentages, for each group. **J,** Western blots analysis of ERα and p53 from the extracts of select tumors at the end of treatment. Anti-vinculin antibody was used to determine the level of protein loaded. Quantification of ERα (**K**) and p53 (**L**) protein levels in select tumors relative to the vinculin loading control. The results are the mean ± SEM. *, *P* ≤ 0.05; ***, *P* ≤ 0.001; *n* = 12.

The efficacy of X15695 as an ERα degrader was assessed by comparing it with fulvestrant, a FDA-approved pure ERα antagonist (9). ERα level was rapidly downregulated by fulvestrant within 4 to 8 hours of treatment recovering gradually thereafter while X15695 showed a rather slow but more sustained downregulation lasting over 24 to 48 hours (Fig. 6A). Furthermore, both compounds at 1 nmol/L were sufficient to destabilize ERα in 8 hours (Fig. 6B). The two compounds were also compared on the basis of their ability to decrease the viability of tamoxifen resistant MCF-7 cells (TRMCF-7). Here, X15695 and fulvestrant showed comparable activities as opposed to tamoxifen that was ineffective (Fig. 6C). In clonal expansion experiments with TRMCF-7 cells, tamoxifen was again inactive while X15695 and fulvestrant were both active, with fulvestrant being slightly more efficacious than X15695 (Fig. 6D and E).

Although oral administration is a more preferred route of drug administration, the low aqueous solubility of fulvestrant requires it to be administered intramuscularly (6–8). X15695 differs from fulvestrant in that it was found to significantly decrease tumor volume and weight within 2 weeks after oral application to a mouse xenograft tumor model (30 mg/kg body weight daily; Fig. 6F and H). In that study, X15695 was well tolerated and did not cause any weight loss or other signs of host toxicity (Fig. 6I). Western blot analysis of lysates from the tumors after the treatment period showed a significant decrease in ERα level and an increase in p53 expression, in line with the results in the cell culture experiments (Fig. 6J and L).

### Regulation of AR^+^ Prostate Cancer Cells by Imidazopyridine Derivatives

**FIGURE 7:**
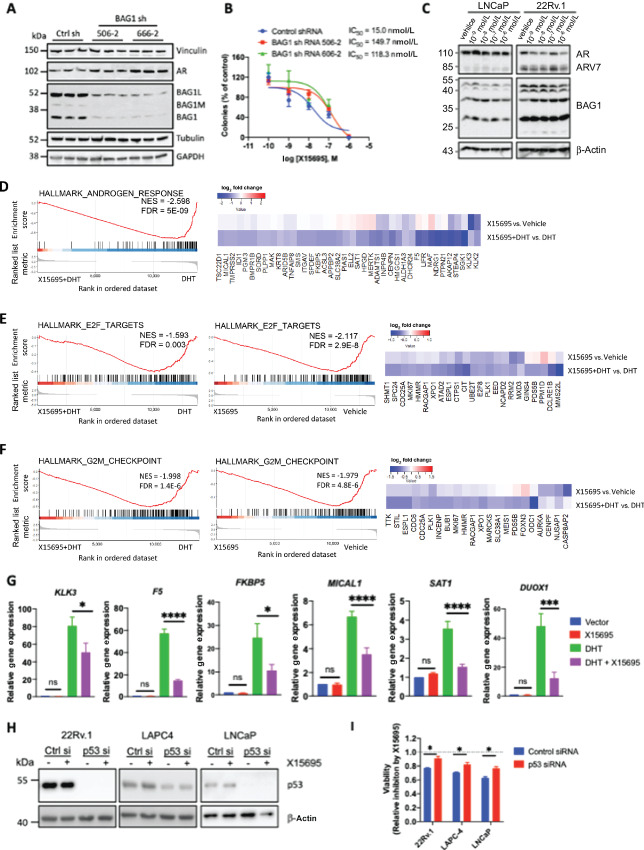
X15695-mediated regulation of AR action in prostate cancer cells. **A,** Western blot analysis of BAG1 in LNCaP cells transduced with BAG-1 shRNA and control shRNA. Antibodies to vinculin, AR, tubulin, and GAPDH were used as controls. **B,** Quantification of the effect of X15695 on clonal expansion of control and BAG1 shRNA knockdown LNCaP cells. Each point represents the mean ± SEM. *n* = 3. **C,** Western blot analysis of AR and BAG1 in extracts of LNCaP and 22Rv.1 cells treated with the indicated concentrations of X15695 for 24 hours. β-actin was used as a loading control. **D–F,** GSEA plots of the topmost signaling pathways and heat maps of log_2_ fold-change in gene expression in the comparison of DHT + X15695 versus DHT and X15695 versus vehicle in LNCaP cells. **G,** Quantitative RT-PCR was carried to detect the effect of X15695 on DHT- mediated expression of the indicated target genes in LNCaP cells. Cells were treated with vehicle or 10 nmol/L DHT and X15695 (5 μmol/L) for 16 hours. The results are the means ± SEM (*n* = 4; *, *P* ≤ 0.05; ***, *P* ≤ 0.001; ****, *P* ≤ 0.0001; ns, not significant). **H,** 22Rv.1, LAPC-4, and LNCaP cells were transfected with control and p53 siRNA for 16 hours and treated with vehicle or X15695 (5 μmol/L) for 24 hours. Western blot analysis was performed with anti-p53 and anti-β-actin antibodies. **I,** MTT cell viability assay carried out with 22Rv.1, LAPC-4, and LNCaP cells previously transfected with control and p53 siRNA and treated with vehicle and X15695 for 72 hours. The results are the mean ± SEM (*n* = 3; *, *P* ≤ 0.05).

As the imidazopyridines analyzed for the inhibition of clonal expansion of ER^+^ breast cancer cells also inhibited proliferation of AR^+^ prostate cancer cells (Supplementary Table S1), it is possibility that these compounds function through a common antitumor pathway. We therefore first investigated whether BAG1 played a role in the action of X15695 in the prostate tumor cells using BAG1 knockdown LNCaP prostate cancer cells. Two BAG1 shRNA clones that showed a clear reduction in BAG1 level compared with the control clone (Fig. 7A) showed a decrease in the IC_50_ concentration for inhibition of clonal expansion (Fig. 7B), suggesting a contribution of BAG1 to proliferation of these cells. Unlike the breast cancer studies, treatment with X15695 did not strongly downregulate AR level in LNCaP prostate cancer cells nor in the castration-resistant prostate cancer (CRPC) cell line 22Rv.1 (Fig. 7C). Nonetheless, transcriptomics experiments carried out with LNCaP cells showed an X15695-mediated attenuation of androgen response. Specifically, GSEA showed that in the presence of DHT, androgen signaling pathway, G_2_–M cell-cycle arrest, and E2F pathways were attenuated by X15695, while only the latter two were attenuated in the absence of DHT (Fig. 7D and F). RT-PCR studies confirmed that X15695 downregulated a select number of classical AR target genes (*KLK3*, *FKBP5*, *F5*) and androgen-induced ROS genes (*MICAL1*, *SAT1*, *DUOX1*) previously identified as BAG1 sensitive targets (20) and also identified in the present RNA-seq study (Fig. 7D and G). X15695 was found to be a better inhibitor of AR-target gene expression compared with the five other imidazopyridines identified previously (compare Fig. 7G with Supplementary Fig. S4). However, in studies to determine the ability of the imidazopyridines to stabilize p53 expression in three different prostate cancer cell lines (22Rv.1 LNCaP, LAPC-4), none of the compounds strongly stabilized p53 (Supplementary Fig. S5A). However, we focused on X15695 for further analysis in the prostate cancer cells for better comparison with the studies in the breast cancer cells.

**FIGURE 8:**
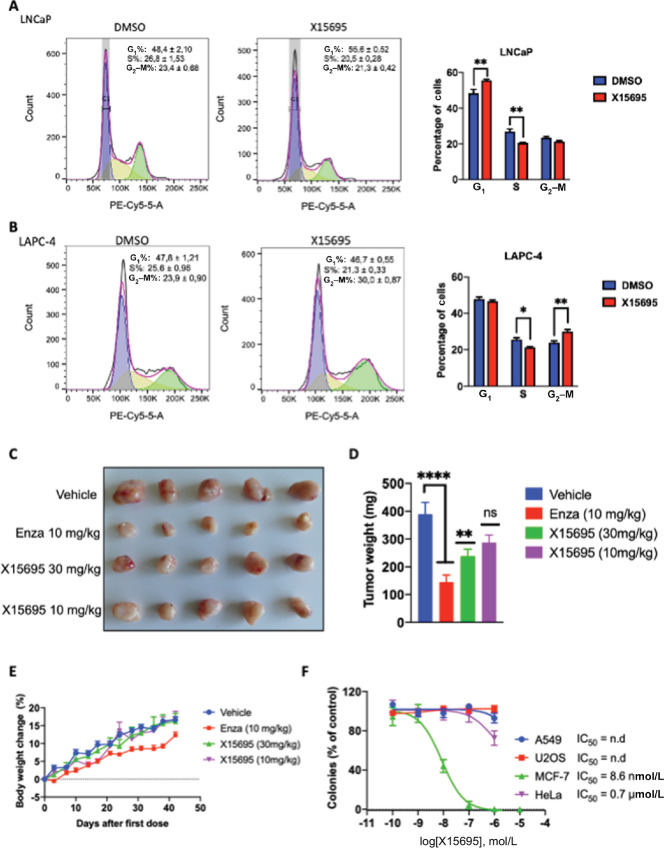
X15695 induces cell-cycle arrest and inhibits proliferation of AR^+^ and ER^+^ prostate and breast cancer cells. Cell-cycle profile measured by flow cytometry of LNCaP (**A**) and LAPC-4 cells (**B**) treated with vehicle or 1 μmol/L X15695 for 48 hours. Cells for flow cytometry were stained with DRAQ5. The quantified results are the mean ± SEM (*n* = 3; *, *P* ≤ 0.05; **, *P* ≤ 0.01). **C,** Representative examples of LAPC-4 xenograft mouse tumors after daily treatment with vehicle, X15695 (10 and 30 mg/kg), and enzalutamide (10 mg/kg) for 43 days. **D,** Tumor weights at the end of treatment were recorded and presented as a bar chart. The values are the means ± SEM expressed (**, *P* ≤ 0.01; ****, *P* ≤ 0.0001; *n* = 14; ns, not significant). **E,** Mouse body weights were measured twice weekly over the 43 days. Presented are the mouse body weight changes, in percentages, for each group. **F,** Quantification of the effect of the indicated concentrations of X15695 on clonal expansion of the indicated tumor cells (*n* = 3).

We next investigated whether X15695 caused nuclear translocation of p53 as was observed in the MCF-7 cells. Note that the prostate cancer cells have different p53 status: LNCaP cells express wild-type p53; 22Rv.1 cells express wtp53/mtp53^Q331R^ and LAPC-4 cells express mtp53^R175H^. In immunofluorescence experiments, X15695 promoted a cytoplasmic/nuclear translocation of p53 in cells that express wild-type p53 (LNCaP and 22Rv.1) but not in the LAPC-4 cells that express only mutant p53. In the latter cell line, p53 was already nuclear and X15695 did not alter its cellular localization (Supplementary Fig. S5B). To determine whether p53 plays a role in the inhibitory action of X15695 in the prostate cancer cells, an attenuation of the X15695-mediated decrease in viability of the three prostate cancer cells was determined after p53 knockdown by siRNA transfection (Fig. 7H). Decreased viability observed after X15695 treatment was significantly compromised in all three cell lines transfected with p53 siRNA (Fig. 7I), indicating a contribution of p53 to the growth inhibitory action of X15695 in these cells. Flow cytometric analysis was also determined to assess the downstream effects of p53 on cell-cycle arrest or the induction of apoptosis in the prostate cancer cells following treatment with X15695. We found out that X15695 induced G_1_–S-phase arrest in the LNCaP cells and a G_2_–M arrest in the LAPC-4 cells (Fig. 8A and B). No apoptotic effect of X15695 was identified in either prostate cancer cell type (Supplementary Fig. S6), indicating an effect mediated primarily by the cell-cycle arrest.

### X15695 Inhibits Prostate Tumor Growth in a Mouse Xenograft Model

To determine whether the cell-cycle block and the antiandrogen action of X15695 are sufficient to inhibit prostate tumor growth, we carried out mouse tumor xenograft experiments using LAPC-4 cells. We compared the action of X15695 (10 and 30 mg/kg) with the classical antiandrogen enzalutamide (10 mg/kg) over 42 days. X15695 effectively inhibited tumor growth over vehicle upon oral administration (30 mg/kg/day), albeit less effectively compared with enzalutamide (10 mg/kg/day; Fig. 8C and D), identifying X15695 as a weaker AR antagonist in this tumor model. As with the MCF-7 cell xenograft experiment, X15695 was well tolerated in the study with no signs of toxicity or weight loss (Fig. 8E).

### X15695 is Selective for ER^+^ Breast Cancer

The negative action of X15695 on proliferation of AR^+^ prostate and ER^+^ breast cancer, posed the question whether the proliferation of other tumor cells could be inhibited by this compound. X15695’s effect on the clonal expansion of cervical, lung, and osteosarcoma (HeLa, A549, and U2OS) cells was compared with its effect on MCF-7 cells. While X15695 dose-dependently inhibited the clonal expansion of MCF-7 cells, it did not have any significant effect on the other tumors cell lines (Fig. 8F).

Collectively, these results demonstrate that X15695 inhibits proliferation of breast cancer cells and prostate cancer cells but its strong effect on the degradation of ERα combined with the reactivation of p53 makes it a superior inhibitor of ER^+^ breast cancer cells than AR^+^ prostate cancer cells. These effects combined with its lack of action in inhibiting proliferation of other cancer cells warrants X15695 to be classified as an oral SERD.

## Discussion

In this study, a compound with an imidazopyridine scaffold X15695 was shown to strongly downregulate ERα activity and to a lower extent AR action and to reactivate wild-type and mutant p53. As a consequence, X15695 inhibited proliferation of ER^+^ breast cancer and AR^+^ prostate cancer cells *in vitro* and *in vivo* in xenograft models. However, in the *in vivo* studies, X15695 was more efficacious in inhibiting breast cancer than prostate cancer cell proliferation. X15695 action was selective as no inhibition of proliferation was detected in steroid receptor negative breast or prostate cancer cells or other tumor cells analyzed that lack expression of gonadal steroid receptors.

Imidazopyridine-based compounds to which X15695 belongs have gained significant attention in medicinal chemistry due to their frequent occurrence in a large number of marketed drug formulations and drug candidates. These compounds have a wide variety of biological and pharmacologic activities such as antimycobacterial, antidiabetic, antiviral, and anticancer activities (41). For anticancer action, a library of amide derivatives of imidazopyridine has been shown to be highly potent in inhibiting the proliferation of breast (MCF-7, MDA MB-231), lung (A549), and prostate (DU145) cancer cell lines (42). Recently, imidazopyridine compounds that inhibit PI3K/Akt and the proliferation of AR^+^ and AR^–^ CRPC cells have been described (43, 44). However, the high concentrations of these compounds (up to 10 μmol/L) required for the inhibition of CRPC proliferation question their on-target mode of action.

Several *in vitro* experimental observations have shown that the anticancer effect of the imidazopyridine compounds results mainly from their inhibitory effects on six main molecular targets: PI3K/Akt, centromere-associated protein E (CENP-E), insulin-like growth factor-1 receptor (IGF-1R), cyclin-dependent kinases (CDK), tubulin polymerization, and hepatocyte growth factor receptor (45). So far, steroid receptors and p53 have not been identified as molecular targets for imidazopyridines.

We have shown that X15695 inhibits proliferation of tumor cells that express ERα and AR as well as the transactivation function of these two receptors but its effect is more profound on ERα compared with the AR-expressing tumor cells. Although the primary target(s) of action of X15695 have not been identified in this work, there is a link to the BAG domain of the BAG family of proteins through docking experiments performed with the related A4B17 molecule (19). The BAG family is a multifunctional protein family that performs diverse functions ranging from the control of cell proliferation, apoptosis to tumorigenesis. This family is made up of six proteins [BAG1, BAG2, BAG3 (CAIR-1/Bis), BAG4 (SODD), BAG5, and BAG6], each with at least one conserved BAG domain made up of three anti-parallel alpha helical bundles of 30–40 amino acids (12). They use this BAG domain to interact with the ATPase binding domain of the molecular chaperone HSP70/HSC70 to function as nucleotide exchange factors (46). BAG domain/HSP70/HSC70 complexes have also been reported to interact with several client proteins to mediate the biological action of these proteins and small molecules that disrupt these interactions have been suggested to be potential therapeutic drugs (47).

We have previously reported that A4B17 that is structurally related to X15695 disrupts interaction of BAG1L with the N-terminus of the AR, that is otherwise required for the activity of this receptor (19). A possibility exists that X15695 also disrupts interaction of BAG1 with ERα (16) to account for its inhibitory action on E_2_ target gene expression in the ER^+^ breast cancer cells. We have also shown that X15695 regulates p53 action by disrupting the interactions of mortalin with wild-type p53 and also of BAG1 with mortalin. Although the disruption of mortalin–p53 interaction by X15695 could be a result of its action on mortalin–BAG1 interaction, we cannot rule out an independent direct action of X15695 on mortalin–p53 interaction. We showed that treatment with X15695 disrupts the cytoplasmic retention of p53 by mortalin leading to nuclear translocation of p53 and the activation of the cell-cycle regulator p21 and the apoptotic action of p53. Reactivation of p53 has become a challenge in drug discovery programs for anticancer therapies. Several small-molecule inhibitors are reported to disrupt mortalin–p53 interaction to reactivate p53. Among them is Mortaparib^plus^ that inhibits the proliferation of MCF-7 cells. However, Mortaparib^plus^ reactivates only wild-type p53 and not mutant p53 in T47D cells (35, 48). Other compounds such as PRIMA-1 and its methyl analog Eprenetapopt (APR-246) are reported to reactivate mutant p53 (49, 50). APR-246, for example, is a first-in-class small molecule that restored wild-type p53 functions in *TP53*-mutant cells and is currently in phase II clinical trials (51). Unlike Mortaparib^plus^ and APR-246, we have shown in this study that X15695 reactivates both wild-type and mutant p53. With mutant p53, we have shown that it disrupts its interaction with BAG2 and the GOF action of mutant p53. These findings together speak in favor of X15695 being a regulator of protein–protein interactions involving the HSP70/HSC70 chaperone/co-chaperone complex.

Two main types of mutant “hotspot” sites exist in p53: contact mutants (R273H, R248Q, and R248W) and conformational mutants (R175H, G245S, R249S, and R282H), both affecting the DNA-binding activity and the transcriptional downstream targets of p53 (52). Contact mutants generally produce structural changes in the p53 protein that directly affect DNA binding, while conformational mutants generate structural changes related to protein folding, but both types of mutants have GOF activities (53). So far BAG2 and BAG5 are reported to bind both types of mutant p53 (39, 40) for their tumorigenic activities. From our results in this work on X15695-mediated disruption of BAG2–mutp53 interaction, the proliferation of a large number of tumor cells with mutant p53 status would be expected to be inhibited by X15695. This was evidently not the case as X15695 did not significantly alter the proliferation of the ER^−^ breast cancer cell line MDA-MB231 that expresses mutp53 (R280K), or the AR^−^ prostate tumor cell line DU145 that expresses mutp53 (P223L/V274F). These findings suggest that other regulatory factors in addition to p53 are required for X15695 to exert its maximum inhibitory action. These other regulatory factors could be the steroid receptors.

One of the important findings of this work is that X15695 downregulates ERα levels which puts it in the class of ER degraders to which the clinically approved antiestrogen fulvestrant belongs. However, unlike fulvestrant that requires intramuscular injection (9), X15695, when given orally, reduces ERα levels in two weeks in a xenograft tumor model. A further advantage of X15695 is that it induces cell-cycle arrest and apoptosis to enhance its antitumor action in breast cancer cells without the need for the combination drug therapy suggested for fulvestrant. To improve the anticancer action of fulvestrant, it is recommended to have it administered with other targeted therapies such as CDK4/CDK6 inhibitors (e.g., Palbociclib) that promote cell-cycle block at the G_1_–S phase (54). X15695 decreases ERα levels and induces cell cycle arrest and apoptosis on its own. The multi-targeting of factors by one compound characterizes the uniqueness of action of X15695 in this study. The actions of X15695 described here identify X15695 as a selective oral ERα degrader that warrants further development as an ER^+^ breast cancer therapeutic.

## Supplementary Material

Supplementary Data Supplementary data and Supplementary figure legends.

Supplementary Table S1 Potency of inhibition of colony formation by different imidazopyridines in breast and prostate cancer cells.

Supplementary Table S2 Crystallographic data and structure refinement information for X15695, X15696 and X19168.

Figure S1 Volcano plots and Hallmark gene set analyses showing pathways targeted by X15695.

Figure S2 X15695-mediated attenuation of ERalpha target gene expression and reactivation of p53.

Figure S3 X15695 alters the cellular localization of wild-type p53 but not mutant p53.

Figure S4 X15695 derivatives attenuate AR target gene expression.

Figure S5 X15695-mediated regulation of expression and cellular localization of p53 in prostate cancer cells.

Figure S6 X15695 does not induce apoptosis in LNCaP and LAPC-4 cells.
