# Supplementary material for: Identification of an Imidazopyridine-based Compound as an Oral Selective Estrogen Receptor Degrader for Breast Cancer Therapy
Source: Cancer Res Commun. 2023 Jul 27;3(7):1378–96. doi: 10.1158/2767-9764.CRC-23-0111 (PMC10373600; doi:10.1158/2767-9764.CRC-23-0111)
Supplement: Supplementary Data — and Supplementary figure legends. [file crc-23-0111-s01.docx]

**Supplementary Data**

**Identification of an imidazopyridine-based compound as an oral selective estrogen receptor degrader for breast cancer therapy**

List of compounds that were ordered and used as provided

| Compound label | vendor | Purity* | CAS-number |
| --- | --- | --- | --- |
| X15694 | MolPort | n/a | [2069-47-8](https://scifinder-n.cas.org/navigate/?appId=9310c7d5-6287-4f24-a3cc-c9fa24393ac4&backKey=636b8c47d83e1d77fc0180af&backToPage=1&contentUri=substance%2Fpt%2F2069478&key=636b8c47d83e1d77fc0180af&metricsOrdinal=1&metricsResultType=substance&ordinal=1&resultType=substance&resultView=DETAIL&sortBy=relevance&sortOrder=descending&state=searchDetail.substance&uiContext=365&uiSubContext=551&uriForDetails=substance%2Fpt%2F2069478) |
| X15688 | MolPort | n/a | [724742-90-9](https://scifinder-n.cas.org/navigate/?appId=9310c7d5-6287-4f24-a3cc-c9fa24393ac4&backKey=636b8ca2d83e1d77fc0186cd&backToPage=1&contentUri=substance%2Fpt%2F724742909&key=636b8ca2d83e1d77fc0186cd&metricsOrdinal=1&metricsResultType=substance&ordinal=1&resultType=substance&resultView=DETAIL&sortBy=relevance&sortOrder=descending&state=searchDetail.substance&uiContext=365&uiSubContext=551&uriForDetails=substance%2Fpt%2F724742909) |
| X15692 | MolPort | n/a | [724742-88-5](https://scifinder-n.cas.org/navigate/?appId=9310c7d5-6287-4f24-a3cc-c9fa24393ac4&backKey=636b8ce6d83e1d77fc018b26&backToPage=1&contentUri=substance%2Fpt%2F724742885&key=636b8ce6d83e1d77fc018b26&metricsOrdinal=1&metricsResultType=substance&ordinal=1&resultType=substance&resultView=DETAIL&sortBy=relevance&sortOrder=descending&state=searchDetail.substance&uiContext=365&uiSubContext=551&uriForDetails=substance%2Fpt%2F724742885) |
| X15696 | MolPort | n/a | [284674-61-9](https://scifinder-n.cas.org/navigate/?appId=9310c7d5-6287-4f24-a3cc-c9fa24393ac4&backKey=636b8d3dd83e1d77fc019155&backToPage=1&contentUri=substance%2Fpt%2F284674619&key=636b8d3dd83e1d77fc019155&metricsOrdinal=1&metricsResultType=substance&ordinal=1&resultType=substance&resultView=DETAIL&sortBy=relevance&sortOrder=descending&state=searchDetail.substance&uiContext=365&uiSubContext=551&uriForDetails=substance%2Fpt%2F284674619) |
| X15695 | MolPort | n/a | [353258-25-0](https://scifinder-n.cas.org/navigate/?appId=9310c7d5-6287-4f24-a3cc-c9fa24393ac4&backKey=636b8d8dd83e1d77fc019690&backToPage=1&contentUri=substance%2Fpt%2F353258250&key=636b8d8dd83e1d77fc019690&metricsOrdinal=1&metricsResultType=substance&ordinal=1&resultType=substance&resultView=DETAIL&sortBy=relevance&sortOrder=descending&state=searchDetail.substance&uiContext=365&uiSubContext=551&uriForDetails=substance%2Fpt%2F353258250) |
| X15689 | MolPort | n/a | [353258-27-2](https://scifinder-n.cas.org/navigate/?appId=9310c7d5-6287-4f24-a3cc-c9fa24393ac4&backKey=636b8dced83e1d77fc019a81&backToPage=1&contentUri=substance%2Fpt%2F353258272&key=636b8dced83e1d77fc019a81&metricsOrdinal=1&metricsResultType=substance&ordinal=1&resultType=substance&resultView=DETAIL&sortBy=relevance&sortOrder=descending&state=searchDetail.substance&uiContext=365&uiSubContext=551&uriForDetails=substance%2Fpt%2F353258272) |

* as given by vendor

List of compounds that are available for further re-use cases and can be requested at the Molecule Archive of KIT ([compound-platform@ioc.kit.edu](mailto:compound-platform@ioc.kit.edu))

| Date | Product smiles | X-number | available |
| --- | --- | --- | --- |
| 09.01.2023 | Cc1cn2cc(nc2c(c1)Cl)c1ccccc1 | X19728 | > 10 mg |
| 09.01.2023 | Fc1ccc(cc1)c1cn2c(n1)c(Cl)cc(c2)C | X19724 | > 10 mg |
| 09.01.2023 | Clc1cc(cn2c1nc(c2)c1ccccc1)C(F)(F)F | X19166 | > 10 mg |
| 09.01.2023 | Fc1ccccc1c1cn2c(n1)c(Cl)cc(c2)C(F)(F)F | X19720 | > 10 mg |
| 09.01.2023 | Fc1ccc(cc1)c1cn2c(n1)c(Cl)cc(c2)C(F)(F)F | X15695 | > 10 mg |
| 09.01.2023 | Fc1ccc(cc1)c1cn2c(n1)ccc(c2)C(F)(F)F | X20046 | > 10 mg |
| 09.01.2023 | c1ccc(cc1)c1nc2n(c1)cccc2 | X19147 | > 10 mg |
| 09.01.2023 | Fc1ccccc1c1nc2n(c1)cccc2 | X19712 | > 10 mg |
| 09.01.2023 | Fc1ccccc1c1cn2c(n1)c(C)ccc2 | X19718 | > 10 mg |
| 09.01.2023 | Fc1ccc(cc1)c1cn2c(n1)c(C)ccc2 | X19167 | > 10 mg |
| 09.01.2023 | Clc1cccn2c1nc(c2)c1ccccc1 | X19726 | > 10 mg |
| 09.01.2023 | Fc1ccccc1c1cn2c(n1)c(Cl)ccc2 | X19727 | > 10 mg |
| 09.01.2023 | Fc1ccc(cc1)c1cn2c(n1)c(Cl)ccc2 | X19725 | > 10 mg |
| 09.01.2023 | Brc1ccc2n(c1)cc(n2)c1ccccc1 | X19148 | > 10 mg |
| 09.01.2023 | Brc1ccc2n(c1)cc(n2)c1ccccc1F | X19719 | > 10 mg |
| 09.01.2023 | Fc1ccc(cc1)c1cn2c(n1)ccc(c2)Br | X19168 | > 10 mg |
| 09.01.2023 | Cc1ccc2n(c1)cc(n2)c1ccccc1 | X20034 | > 10 mg |
| 09.01.2023 | Cc1ccc2n(c1)cc(n2)c1ccccc1F | X20035 | > 10 mg |
| 09.01.2023 | Fc1ccc(cc1)c1cn2c(n1)ccc(c2)C | X20036 | > 10 mg |
| 09.01.2023 | Cc1cn2cc(nc2c(c1)Cl)c1ccccc1F | X19729 | > 10 mg |
| 09.01.2023 | FC(c1ccc2n(c1)cc(n2)c1ccccc1)(F)F | X20048 | > 10 mg |
| 09.01.2023 | Fc1ccccc1c1cn2c(n1)ccc(c2)C(F)(F)F | X20047 | > 10 mg |
| 09.01.2023 | Cc1cccn2c1nc(c2)c1ccccc1 | X19151 | > 10 mg |
| 09.01.2023 | Fc1ccc(cc1)c1cn2c(n1)ccc(c2)Cl | X15694 | < 10 mg |
| 09.01.2023 | Ic1ccc(cc1)c1cn2c(n1)ccc(c2)C(F)(F)F | X15688 | < 10 mg |
| 09.01.2023 | Brc1ccc(cc1)c1cn2c(n1)ccc(c2)C(F)(F)F | X15692 | < 10 mg |
| 09.01.2023 | Clc1cc(cn2c1nc(c2)c1ccccc1)C(F)(F)F | X15696 | < 10 mg |
| 09.01.2023 | Fc1ccc(cc1)c1cn2c(n1)c(Cl)cc(c2)C(F)(F)F | X15695 | < 10 mg |
| 09.01.2023 | Clc1ccc(cc1)c1cn2c(n1)c(Cl)cc(c2)C(F)(F)F | X15689 | < 10 mg |

**List of primer sequences used.**

Rib36B4 For 5´-CTCCTGAGCGCAAGTACTCC-3´

Rev 5´-GTCACCTTCACCGTTGTTCCA-3´

KLK3 For 5’ – CCCGGTTGTCTTCCTCACCC-3’

Rev-5’ – GCCTCCCACAATCCGAGACA-3’

F5 For 5´-TCCAGGCCGAGAATACACCTA-3´

Rev 5´-CGATTTGCTTGTCAAACGTCTTC-3´

DUOX1 For 5´-GTGCTCCCTCTGTTGTTCGT-3´

Rev 5´-GCTTCTCAGACACGATGCTCT-3´

MICAL1 For 5´-ATGGGCAGCCTGATGTCTCT-3´

Rev 5´-GGCGCCATGCTTCTCTTG-3´

SAT1 For 5‘ -GCCGACTGGTGTTTATCCGT-3’

Rev 5‘ -TTTCTTCCCTTTGCGGACCA-3’

FKBP5 For 5´-TTCAAGGGAGGCAAATACATG-3´

Rev 5´-TCCAAGGGCCTTGTCACAG-3´

SLC7A11 For 5´-ATGCAGTGGCAGTGACCTTT-3´

Rev 5´-CATGGAGCCAAAGCAGGAGA-3´

OSGIN For 5´-AGAAGAAGCGAAGAGGTC-3´

Rev 5´-CGGACACAAAGTTATGCC-3´

p53 For 5´-TGCGTGTTTGTGCCTGTCCT-3´

Rev 5´-GTGCTCGCTTAGTGCTCCCT-3´

CDKN1A(p21) For 5´-GACTCTCAGGGTCGAAAACG-3´

Rev 5´-GCGGATTAGGGCTTCCTCTT-3´

GADD45A For 5´-CTCAACGTCGACCCCGATAA -3´

Rev 5´-GCCTGGATCAGGGTGAAGTG-3´

BB3 (PUMA) For 5´-GACCTCAACGCACAGTACGAG-3´

Rev 5´-AGGAGTCCCATGATGAGATTGT-3´

FAS For 5´-ACACCAAGTGCAAAGAGGAAGGAT-3´

Rev 5´-GACCAAGCTTTGGATTTCATTTCTG-3´

PGR For 5´-CTTAATCAACTAGGCGAGAG-3´

Rev 5´-AAGCTCATCCAAGAATACTG-3´

TFF1 For 5´-CAATGGCCACCATGGAGAAC-3´

Rev 5´-AACGGTGTCGTCGAAACAGC-3´

GREB1 For 5´- GAGTGACAATGAGGAAGAG-3´

Rev 5´-CTCGTTGGAAATGGAGACAA-3´

AREG For 5´-TGAGATGTCTTCAGGGAGTG-3´

Rev 5´-AGCCAGGTATTTGTGGTTCG-3´

AGR2 For 5´-GACC AAGCTTTGGATTTCATTTCTG-3´

Rev 5´-CAGGTTCGTAAGCATAGAGACG-3´

PDZK1 For 5´-GCAGGCTCAGAACAGAAAGG-3´

Rev 5´- TCCAGGGTTTCCACAGACTC-3´

**Synthesis of imidazopyridines**

Compounds ordered and used as described by vendor are listed in Supplementary Table S1.

**Synthesis**

# General Remarks

The starting materials and reagents were purchased from abcr, Acros, Alfa Aesar, ChemPUR, Fluka, Fluorochem, Merck, Sigma Aldrich, Strem, TCI, or Thermo Fisher Scientific and used without further purification unless otherwise stated. Solvents of technical grade were purified via distillation prior to use (ethyl acetate, dichloromethane, cyclohexane), solvents of *p.a.* quality were purchased from Acros, Fisher Scientific, Sigma Aldrich, Roth, or Riedel-de Haën and were used without further purification. Air- and moisture-sensitive reactions were carried out under nitrogen or argon atmosphere in oven-dried glassware using standard Schlenk techniques.

Reactions in vials were sealed with Crimp caps; both vials and caps were purchased at Chroma Globe. Solvents were evaporated under reduced pressure at 40 °C using a rotary evaporator. For solvent mixtures, each solvent was measured volumetrically.

**Flash Chromatography:**

Purifications via flash chromatography were performed using silica gel (SiO_2_, 0.040 mm × 0.063 mm, Merck) and quartz sand (glowed and purified with hydrochlorid acid). After removing the solvent under reduced pressure, the crude products were immobilized on Celite (Sigma Aldrich) and applied to the column as a solid.

For automatic flash chromatography, an Interchim PuriFLASH XS 400, Interchim PuriFLASH 4125 or Interchim PuriFLASH 5.125 was used in combination with hand-packed silica columns (SiO_2_, 0.040 mm × 0.063 mm, Merck) as well as prepacked SIHP (silica high performance, 15 µm, 4 g/12 g/40 g/80 g) columns from Interchim. Fractions were separated and collected using a diode array detector (DAD).

**Thin Layer Chromatography (TLC):**

Reactions were monitored by thin-layer chromatography (TLC) using silica-coated aluminum plates (Merck, silica gel 60, F_254_). UV active compounds were detected with a UV-lamp (Hanau Quarzlampen, Typ 204 AC) at 254 nm and 366 nm excitation. Moreover Seebach solution (2.5 % phosphomolybdic acid, 1.0 % Cerium(IV)sulfate tetrahydrate, 6.0 % conz. H_2_SO_4_ in H_2_O) with subsequent heating of the TLC plate was used to stain the spots.

Liquid-Chromatography Mass Spectrometry (LC-MS) was conducted using a device from Agilent with HP 1100 MSD G1946 Mass Detector and a Kinetex XB-C18 column (2.6 µm, 100 x 4.60 mm) from Phenomenex. API-ES was used as a method of ionization and the following program was applied:

*10_99_P* (positive polarity): injector volume 10.0 µl, flow rate 1.0 ml/min, run time 20.0 min,
solvent: water (bidistilled) 50%, acetonitrile 20%.

**Melting Points:**

Melting points were detected on an OptiMelt MPA100 device from Stanford Research System.

**Nuclear Magnetic Resonance Spectroscopy (NMR):**

A Bruker Ascend 400 was used to record NMR spectra; ^1^H-NMR spectra were measured at 400 MHz, ^13^C-NMR spectra at 100 MHz and ^19^F-NMR spectra at 376 MHz. All measurements were conducted at room temperature using CDCl_3_ or DMSO-*d*_6_ acquired from Eurisotop and Sigma Aldrich as solvents and accordingly referenced to CDCl_3_ (^1^H 7.27 ppm, s / ^13^C 77.0 ppm, t) or DMSO-*d*_6_ (^1^H 2.50 ppm, s / ^13^C 39.52 ppm, sep).

Chemical shifts are given in ppm (parts per million) and the spectra were analyzed following first order spectra. The signal area was given for multiplets whereas the signal center was used for centrosymmetric signals. The signal splitting was characterized using the following abbreviations: s (singlet), d (doublet), t (triplet), q (quartet), m (multiplet), bs (broad singlet), dd (doublet of the doublet), td (triplet of the doublet) etc. For ^13^C spectra the peaks were observed as singlet if not specifically stated otherwise.

Coupling constants *J* are given in Hz (Hertz) and the number of bonds between the coupling cores is indicated as superscripted index in front of the coupling constant. Signals of the ^13^C spectra were assigned using DEPT90 and DEPT135 spectra (distortionless enhanced polarization transfer) as well as HSQC (heteronuclear single quantum coherence) and HMBC (heteronuclear multiple bond correlation).

**Infrared Spectroscopy (IR):**

IR spectra were measured via ATR (Attenuated Total Reflection) on a Bruker IFS 88. The positions of the absorption bands are given in wavenumbers *ṽ* in cm^–1^ and were measured in the range from 3600 cm^–1^ to 500 cm^–1^.

Characterization of the absorption bands was done in dependence of the absorption strength with the following abbreviations: vs (very strong, 0−9%), s (strong, 10−39%), m (medium, 40−69%), w (weak, 70−89%), vw (very weak, 90−100%).

**Mass Spectrometry (MS):**

EI-MS (electron ionization mass spectroscopy) and FAB-MS (fast atom bombardment mass spectrometry) were conducted on a Finnigan Mat 95 with 3-nitrobenzyl alcohol (3-NBA) as matrix and reference for high resolution. The intensity of the signals is given relative to the intensity of the highest peak (100%). For the interpretation of the spectra, molecular peaks [M]+, peaks of protonated molecules [M+H]^+^ and characteristic fragment peaks are indicated with their mass-to-charge ratio (m/z) and their intensity in percent, relative to the base peak (100%) is given. In case of high-resolution measurements, the maximum tolerated error is ±5 ppm.

ESI-MS (electron spray ionization mass spectrometry) was conducted with a ThermoFisher Q Exactive Plus in positive mode with a voltage of 4kV. The tolerated error is ±5 ppm of the molecular mass. The spectra were interpreted by molecular peaks [M]^+^, peaks of protonated molecules [M+H]^+^ and characteristic fragment peaks and indicated with their mass-to-charge ratio (*m/z*).

**Elemental Analysis (EA):**

Elemental analysis was conducted using an Elementar vario Micro and a Sartorius M2P analytical balance. Calculated and found percentage for carbon (C), hydrogen (H), sulfur (S) and nitrogen (N) are indicated in fractions of 100%.

**High Performance Liquid Chromatography (HPLC):**

Preparative Reversed Phase High Performance Liquid Chromatography (RP-HPLC) was performed on the PuriFLASH 4125 system from Interchim. A VDSpher® C18-M-SE precolumn (10 µm, 40 x 16 mm) followed by a PuriFLASH C18-AQ separation column (10 µm, 250 x 21.2 mm) was used as the stationary phase. A gradient of acetonitrile and double distilled water at a flow rate of 15 mL/min served as the mobile phase.

# **Crystallography**

Crystallographic data for compounds **X15695, X15696** and **X19168** reported in this paper have been deposited with the Cambridge Crystallographic Data Centre as supplementary information no. CCDC- 2226035, 2218538 and 2218539. Copies of the data can be obtained free of charge from https://www.ccdc.cam.ac.uk/structures/.

Experimental Details. Single crystal X-ray diffraction data were collected on a STOE STADI VARI diffractometer with monochromated Ga Kα (λ = 1.34143 Å) radiation at 150 K. Using Olex2 (1), the structures were solved with the ShelXT (2) structure solution program using Intrinsic Phasing and refined with the ShelXL (3) refinement package using Least Squares minimization. Refinement was performed with anisotropic temperature factors for all non-hydrogen atoms; hydrogen atoms were calculated on idealized positions. Crystallographic data and structure refinement information for **X15695, X15696** and **X19168** are summarized in Supplementary Table S2.

**8-Chloro-6-methyl-2-phenylimidazo[1,2-a]pyridine** (**X19728**)

Name {X19728}: 8-chloro-6-methyl-2-phenylimidazo[1,2-a]pyridine; Formula: C_14_H_11_ClN_2_; Exact Mass: 242.0611; Smiles: Cc1cn2cc(nc2c(c1)Cl)c1ccccc1; InChIKey: KLJOQBVCGLPIKO-UHFFFAOYSA-N

A mixture of 3-chloro-5-methyl-pyridin-2-amine (150 mg, 1.05 mmol, 1.00 equiv), 2-bromo-1-phenylethanone (209 mg, 1.05 mmol, 1.00 equiv) and sodium hydrogen carbonate (133 mg, 1.58 mmol, 1.50 equiv) in anhydrous toluene (6.00 mL) was stirred for 72 hours at 115 °C. As preparation for the column chromatography (dryload), Celite was added (0.9 g) and the reaction mixture with Celite was evaporated. The obtained crude product was purified via flash-chromatography (Interchim devices puriFLASH 5.125) on silica gel (PF-15SIHP-F0040) using cyclohexane/ethyl acetate 0% to 25% ethyl acetate in 15 column volumes (1 column volume = 91.9 mL; flow: 26 mL/min). The isolated product 8-chloro-6-methyl-2-phenylimidazo[1,2-a]pyridine (253 mg, 1.04 mmol, 99% yield) was obtained as a light orange solid in 99% yield. *R_f_* = 0.50 (cyclohexane/ethyl acetate 2:1).

^1^H NMR (400 MHz, Chloroform-d [7.27 ppm], ppm) δ = 7.98–7.95 (m, 2H), 7.79–7.78 (m, 1H), 7.71 (bs, 1H), 7.44–7.40 (m, 2H), 7.35–7.31 (m, 1H), 7.08 (d, *J* = 1.3 Hz, 1H), 2.28 (d, *J* = 1.0 Hz, 3H); ^13^C NMR (100 MHz, Chloroform-d [77.0 ppm], ppm) δ = 146.0 (C_q_), 142.0 (C_q_), 133.3 (C_q_), 128.6 (2C, CH), 128.0 (CH), 126.5 (CH), 126.2 (2C, CH), 122.4 (C_q_), 122.1 (CH), 121.9 (C_q_), 109.4 (CH), 17.9 (CH_3_); MS (EI, 70 eV, 40 °C), m/z (%): 242/244 (100/31) [M]^+^. HRMS–EI (C_14_H_11_N_2_Cl) *(m/z)*: [M]^+^ Calcd 242.0605; Found 242.0605; IR (ATR, ṽ) = 3128 (w), 3041 (w), 2915 (w), 1720 (w), 1686 (vw), 1636 (w), 1604 (w), 1524 (w), 1473 (m), 1443 (w), 1415 (m), 1340 (m), 1303 (w), 1283 (w), 1264 (w), 1217 (m), 1210 (m), 1149 (w), 1082 (w), 1068 (w), 1020 (w), 943 (w), 918 (w), 901 (m), 846 (w), 823 (vs), 778 (s), 722 (vs), 691 (vs), 637 (w), 616 (w), 565 (m), 558 (w), 528 (s) cm^–1^.

Additional information on the chemical synthesis is available via Chemotion repository:

<https://doi.org/10.14272/reaction/SA-FUHFF-UHFFFADPSC-KLJOQBVCGL-UHFFFADPSC-NUHFF-NUHFF-NUHFF-ZZZ>

Additional information on the analysis of the target compound is available via Chemotion repository:

<https://doi.org/10.14272/KLJOQBVCGLPIKO-UHFFFAOYSA-N.1>

**8-Chloro-2-(4-fluorophenyl)-6-methylimidazo[1,2-a]pyridine** (**X19724**)

Name {X19724}: 8-chloro-2-(4-fluorophenyl)-6-methylimidazo[1,2-a]pyridine; Formula: C_14_H_10_ClFN_2_; Exact Mass: 260.0517; Smiles: Fc1ccc(cc1)c1cn2c(n1)c(Cl)cc(c2)C; InChIKey: CFZOSNJUTXJGRX-UHFFFAOYSA-N

A mixture of 3-chloro-5-methyl-pyridin-2-amine (150 mg, 1.05 mmol, 1.00 equiv), 2-bromo-1-(4-fluorophenyl)ethanone (228 mg, 1.05 mmol, 1.00 equiv) and sodium hydrogen carbonate (133 mg, 1.58 mmol, 1.50 equiv) in anhydrous toluene (6.00 mL) was stirred for 72 hours at 115 °C. As preparation for the column chromatography (dry load), Celite was added (0.9 g) and the reaction mixture with Celite was evaporated. The obtained crude product was purified via flash-chromatography (Interchim devices puriFLASH 5.125) on silica gel (PF-15SIHP-F0040) using cyclohexane/ethyl acetate 0% to 25% ethyl acetate in 15 column volumes (1 column volume = 91.9 mL; flow: 26 mL/min). The isolated product 8-chloro-2-(4-fluorophenyl)-6-methylimidazo[1,2-a]pyridine (207 mg, 794 μmol) was obtained as a light orange solid in 75% yield. *R_f_* = 0.46 (cyclohexane/ethyl acetate 2:1).

^1^H NMR (400 MHz, Chloroform-d [7.27 ppm], ppm) δ = 7.96–7.91 (m, 2H), 7.82 (t, *J* = 1.2 Hz, 1H), 7.75 (s, 1H), 7.14–7.08 (m, 3H), 2.31 (d, *J* = 1.0 Hz, 3H); ^13^C NMR (100 MHz, Chloroform-d [77.0 ppm], ppm) δ = 162.8 (d, *J* = 246.6 Hz, C_q_), 145.2 (C_q_), 142.0 (C_q_), 129.5 (d, *J* = 3.1 Hz, C_q_), 127.9 (d, *J* = 8.5 Hz, CH, 2C), 126.7 (CH), 122.4 (C_q_), 122.1 (C_q_), 122.1 (CH), 115.5 (d, *J* = 21.6 Hz, CH, 2C), 109.1 (CH), 17.9 (CH_3_); ^19^F NMR (376 MHz, ppm) δ = -113.91; MS (EI, 70 eV, 90 °C), m/z (%): 260/262 (100/31) [M]^+^. HRMS–EI (C_14_H_10_N_2_ClF) *(m/z)*: [M]^+^ Calcd 260.0511; Found 260.0510; IR (ATR, ṽ) = 3131 (w), 3080 (w), 3054 (w), 3040 (w), 2953 (w), 2919 (w), 1596 (w), 1548 (w), 1526 (m), 1499 (w), 1477 (s), 1449 (m), 1411 (s), 1377 (w), 1356 (w), 1343 (m), 1293 (w), 1282 (w), 1271 (w), 1211 (vs), 1157 (s), 1123 (w), 1099 (s), 1078 (m), 1041 (w), 1021 (m), 989 (w), 969 (w), 943 (w), 902 (s), 881 (m), 867 (m), 854 (vs), 823 (vs), 805 (vs), 738 (vs), 703 (vs), 674 (m), 662 (s), 633 (m), 605 (m), 569 (m), 543 (s), 531 (vs), 513 (s) cm^–1^.

Additional information on the chemical synthesis is available via Chemotion repository:

<https://doi.org/10.14272/reaction/SA-FUHFF-UHFFFADPSC-CFZOSNJUTX-UHFFFADPSC-NUHFF-NUHFF-NUHFF-ZZZ>

Additional information on the analysis of the target compound is available via Chemotion repository:

<https://doi.org/10.14272/CFZOSNJUTXJGRX-UHFFFAOYSA-N.1>

**8-Chloro-2-phenyl-6-(trifluoromethyl)imidazo[1,2-a]pyridine** **(X19166/X15696)**

Name {X19166/X15696}: 8-chloro-2-phenyl-6-(trifluoromethyl)imidazo[1,2-a]pyridine; Formula: C_14_H_8_ClF_3_N_2_; Exact Mass: 296.0328; Smiles: Clc1cc(cn2c1nc(c2)c1ccccc1)C(F)(F)F; InChIKey: ATHNSQRFCXRPMB-UHFFFAOYSA-N

A mixture of 3-chloro-5-(trifluoromethyl)pyridin-2-amine (150 mg, 763 μmol, 1.00 equiv), 2-bromo-1-phenylethanone (152 mg, 763 μmol, 1.00 equiv) and sodium hydrogen carbonate (96.2 mg, 1.14 mmol, 1.50 equiv) in anhydrous toluene (4.00 mL) was stirred for 16 hours at 115 °C. As preparation for the column chromatography (dryload), Celite was added (0.9 g) and the reaction mixture with Celite was evaporated. The obtained crude product was purified via flash-chromatography (Interchim devices puriFLASH 5.125) on silica gel (PF-15SIHP-F0040) using cyclohexane/ethyl acetate 0% to 10% ethyl acetate in 15 column volumes (1 column volume = 91.9 mL; flow: 26 mL/min). The isolated product 8-chloro-2-phenyl-6-(trifluoromethyl)imidazo[1,2-a]pyridine (112 mg, 379 μmol) was obtained as a colorless solid in 50% yield. *R_f_* = 0.73 (cyclohexane/ethyl acetate 2:1).

^1^H NMR (400 MHz, Chloroform-d [7.27 ppm], ppm) δ = 8.43 (t, *J* = 1.2 Hz, 1H), 8.01–7.98 (m, 3H), 7.48–7.41 (m, 2H), 7.42–7.34 (m, 2H); ^13^C NMR (100 MHz, Chloroform-d [77.0 ppm], ppm) δ = 148.3 (C_q_), 142.8 (C_q_), 132.3 (C_q_), 128.9 (2C, CH), 128.8 (CH), 126.5 (2C, CH), 124.3 (C_q_), 123.1 (q, *J* = 5.8 Hz, CH), 122.9 (q, *J* = 271.3 Hz, CF_3_), 119.5 (q, *J* = 3.1 Hz, CH), 116.7 (q, *J* = 34.7 Hz, C_q_), 110.8 (CH); ^19^F NMR (376 MHz, ppm) δ = -62.02; MS (EI, 70 eV, 30 °C), m/z (%): 332 (16), 330 (25), 296/298 (100/32) [M]+, 196/198 (58/20), 141 (11), 116 (16), 105 (97), 89 (10), 77 (46), 71 (11), 58 (37), 51 (15); HRMS–EI (C_14_H_8_N_2_ClF_3_) *(m/z)*: [M]^+^ Calcd 296.0323; Found 296.0325; IR (ATR, ṽ) = 3122 (w), 3092 (w), 3057 (w), 2924 (w), 2853 (vw), 1714 (w), 1701 (w), 1642 (w), 1598 (w), 1541 (vw), 1476 (w), 1449 (w), 1434 (w), 1377 (m), 1344 (s), 1332 (m), 1316 (vs), 1272 (m), 1237 (w), 1224 (w), 1191 (m), 1171 (vs), 1118 (vs), 1072 (vs), 1027 (m), 992 (s), 958 (m), 933 (m), 922 (w), 890 (s), 857 (vs), 815 (m), 781 (s), 758 (w), 747 (w), 727 (vs), 710 (vs), 691 (vs), 680 (s), 646 (s), 640 (m), 616 (m), 579 (w), 567 (w), 544 (w), 535 (w), 524 (m) cm^–1^.

Additional information on the chemical synthesis is available via Chemotion repository:

<https://doi.org/10.14272/reaction/SA-FUHFF-UHFFFADPSC-ATHNSQRFCX-UHFFFADPSC-NUHFF-NUHFF-NUHFF-ZZZ>

Additional information on the analysis of the target compound is available via Chemotion repository:

<https://doi.org/10.14272/ATHNSQRFCXRPMB-UHFFFAOYSA-N.1>

**8-Chloro-2-(2-fluorophenyl)-6-(trifluoromethyl)imidazo[1,2-a]pyridine** (**X19720**)

Name {X19720}: 8-chloro-2-(2-fluorophenyl)-6-(trifluoromethyl)imidazo[1,2-a]pyridine; Formula: C_14_H_7_ClF_4_N_2_; Exact Mass: 314.0234; Smiles: Fc1ccccc1c1cn2c(n1)c(Cl)cc(c2)C(F)(F)F; InChIKey: RAEZJUWRAYPJAR-UHFFFAOYSA-N

A mixture of 3-chloro-5-(trifluoromethyl)pyridin-2-amine (150 mg, 763 μmol, 1.00 equiv), 2-bromo-1-(2-fluorophenyl)ethanone (166 mg, 763 μmol, 1.00 equiv) and sodium hydrogen carbonate (96.2 mg, 1.14 mmol, 1.50 equiv) in anhydrous toluene (4.00 mL) was stirred for 16 hours at 115 °C. As preparation for the column chromatography (dryload), Celite was added (0.9 g) and the reaction mixture with Celite was evaporated. The obtained crude product was purified via flash-chromatography (Interchim devices puriFLASH 5.125) on silica gel (PF-15SIHP-F0040) using cyclohexane/ethyl acetate 0% to 10% ethyl acetate in 15 column volumes (1 column volume = 91.9 mL; flow: 26 mL/min). The isolated product 8-chloro-2-(2-fluorophenyl)-6-(trifluoromethyl)imidazo[1,2-a]pyridine (164 mg, 520 μmol) was obtained as a colorless solid in 68% yield. *R_f_* = 0.75 (cyclohexane/ethyl acetate 2:1).

^1^H NMR (400 MHz, Chloroform-d [7.27 ppm], ppm) δ = 8.47–8.43 (m, 2H), 8.19 (d, *J* = 3.7 Hz, 1H), 7.42 (d, *J* = 1.5 Hz, 1H), 7.39–7.33 (m, 1H), 7.31–7.27 (m, 1H), 7.16 (ddd, *J* = 1.2 Hz, *J* = 11.5 Hz, *J* = 8.1 Hz, 1H); ^13^C NMR (100 MHz, Chloroform-d [77.0 ppm], ppm) δ = 160.4 (d, *J* = 249.7 Hz, C_q_), 142.0 (C_q_), 141.6 (d, *J* = 1.5 Hz, C_q_), 130.0 (d, *J* = 8.5 Hz, CH), 129.4 (d, *J* = 3.1 Hz, CH), 124.6 (d, *J* = 3.1 Hz, CH), 124.3 (C_q_), 123.3 (q, *J* = 6.2 Hz, CH), 122.9 (q, *J* = 271.5 Hz, CF_3_), 120.3 (d, *J* = 11.6 Hz, C_q_), 119.7 (q, *J* = 2.7 Hz, CH), 116.7 (q, *J* = 34.9 Hz, C_q_), 115.6 (d, *J* = 22.3 Hz, CH), 114.6 (d, *J* = 15.4 Hz, CH); ^19^F NMR (376 MHz, ppm) δ = -62.07 (s), -113.69–-113.76 (m); MS (EI, 70 eV, 50 °C), m/z (%): 314/316 (100/31) [M]^+^, 134 (11); HRMS–EI (C_14_H_7_N_2_ClF_4_) *(m/z)*: [M]^+^ Calcd 314.0228; Found 314.0227; IR (ATR, ṽ) = 3166 (w), 3111 (w), 1728 (vw), 1642 (w), 1581 (w), 1547 (w), 1482 (m), 1448 (w), 1431 (vw), 1384 (m), 1377 (m), 1350 (s), 1330 (vs), 1309 (vs), 1255 (w), 1239 (w), 1222 (m), 1205 (m), 1190 (s), 1174 (s), 1164 (s), 1152 (s), 1125 (vs), 1071 (vs), 1028 (m), 994 (s), 945 (w), 936 (w), 892 (s), 870 (s), 858 (vs), 827 (w), 800 (s), 771 (vs), 749 (vs), 741 (vs), 717 (vs), 703 (vs), 673 (s), 647 (w), 639 (w), 592 (w), 579 (w), 565 (m), 531 (m), 516 (m) cm^–1^.

Additional information on the chemical synthesis is available via Chemotion repository:

<https://doi.org/10.14272/reaction/SA-FUHFF-UHFFFADPSC-RAEZJUWRAY-UHFFFADPSC-NUHFF-NUHFF-NUHFF-ZZZ>

Additional information on the analysis of the target compound is available via Chemotion repository:

<https://doi.org/10.14272/RAEZJUWRAYPJAR-UHFFFAOYSA-N.1>

**8-Chloro-2-(4-fluorophenyl)-6-(trifluoromethyl)imidazo[1,2-a]pyridine** (**X15695**)

Name {X15695}: 8-chloro-2-(4-fluorophenyl)-6-(trifluoromethyl)imidazo[1,2-a]pyridine; Formula: C_14_H_7_ClF_4_N_2_; Exact Mass: 314.0234; Smiles: Fc1ccc(cc1)c1cn2c(n1)c(Cl)cc(c2)C(F)(F)F; InChIKey: LHUTUFIEQFAVFB-UHFFFAOYSA-N

A mixture of 3-chloro-5-(trifluoromethyl)pyridin-2-amine (500 mg, 2.54 mmol, 1.00 equiv), 2-bromo-1-(4-fluorophenyl)ethanone (524 mg, 2.42 mmol, 0.950 equiv) and sodium hydrogen carbonate (321 mg, 3.82 mmol, 1.50 equiv) in anhydrous toluene (15.00 mL) was stirred for 4 days at 115 °C. As preparation for the column chromatography (dryload), Celite was added (1.50 g) and the reaction mixture with Celite were evaporated. The obtained crude product was purified via flash-chromatography (Interchim devices puriFLASH 5.125) on silica gel (PF-15SIHP-F0080) using cyclohexane/ethyl acetate 0% to 10% ethyl acetate in 15 column volumes (1 column volume = 173.2 mL; flow: 34 mL/min). The isolated product 8-chloro-2-(4-fluorophenyl)-6-(trifluoromethyl)imidazo[1,2-a]pyridine (416 mg, 1.32 mmol) was obtained as a colorless solid in 52% yield. *R_f_* = 0.71 (cyclohexane/ethyl acetate 2:1).

^1^H NMR (400 MHz, Chloroform-d [7.27 ppm], ppm) δ = 8.43 (pseudo-t, *J* = 1.2 Hz, 1H), 7.99–7.94 (m, 2H), 7.94 (s, 1H), 7.42 (d, *J* = 1.6 Hz, 1H), 7.17–7.13 (m, 2H); ^13^C NMR (100 MHz, Chloroform-d [77.0 ppm], ppm) δ = 163.2 (d, *J* = 248.1 Hz, C_q_), 147.4 (C_q_), 142.8 (C_q_), 128.6 (d, *J* = 3.1 Hz, C_q_), 128.3 (d, *J* = 8.5 Hz, 2C, CH), 124.3 (C_q_), 123.1 (q, *J* = 5.4 Hz, CH), 122.9 (q, *J* = 271.3 Hz, CF_3_), 119.6 (q, *J* = 3.1 Hz, CH), 116.8 (q, *J* = 35.1 Hz, C_q_), 115.8 (d, *J* = 22.3 Hz, 2C, CH), 110.4 (CH); ^19^F NMR (376 MHz, ppm) δ = -62.02, -112.47; MS (EI, 70 eV, 60 °C), m/z (%): 314/316 (100/33) [M]^+^, HRMS–EI (C_14_H_7_N_2_ClF_4_) *(m/z)*: [M]^+^ Calcd 314.0228; Found 314.0227; IR (ATR, ṽ) = 3126 (w), 3092 (w), 3060 (w), 1723 (w), 1642 (w), 1601 (w), 1548 (w), 1486 (s), 1456 (w), 1432 (w), 1417 (w), 1375 (m), 1349 (s), 1329 (vs), 1307 (vs), 1272 (w), 1237 (w), 1218 (vs), 1193 (s), 1170 (vs), 1156 (vs), 1125 (vs), 1101 (vs), 1074 (vs), 1013 (m), 994 (s), 946 (w), 935 (m), 891 (s), 863 (m), 850 (vs), 823 (s), 803 (vs), 742 (vs), 698 (vs), 676 (s), 666 (m), 647 (m), 632 (m), 602 (m), 568 (m), 541 (s), 527 (s), 511 (m) cm^–1^.

Additional information on the chemical synthesis is available via Chemotion repository:

<https://doi.org/10.14272/reaction/SA-FUHFF-UHFFFADPSC-LHUTUFIEQF-UHFFFADPSC-NUHFF-NUHFF-NUHFF-ZZZ>

Additional information on the analysis of the target compound is available via Chemotion repository:

<https://doi.org/10.14272/LHUTUFIEQFAVFB-UHFFFAOYSA-N.1>

**2-(4-Fluorophenyl)-6-(trifluoromethyl)imidazo[1,2-a]pyridine** (**X20046**)

Name {X20046}: 2-(4-fluorophenyl)-6-(trifluoromethyl)imidazo[1,2-a]pyridine; Formula: C_14_H_8_F_4_N_2_; Exact Mass: 280.0624; Smiles: Fc1ccc(cc1)c1cn2c(n1)ccc(c2)C(F)(F)F; InChIKey: RFCPFCDGJGHFCX-UHFFFAOYSA-N

A mixture of 5-(trifluoromethyl)pyridin-2-amine (200 mg, 1.23 mmol, 1.00 equiv), 2-bromo-1-(4-fluorophenyl)ethanone (268 mg, 1.23 mmol, 1.00 equiv) and sodium hydrogen carbonate (155 mg, 1.85 mmol, 1.50 equiv) in anhydrous toluene (6.00 mL) was stirred for 16 hours at 115 °C. As preparation for the column chromatography (dryload), Celite was added (0.6 g) and the reaction mixture with Celite were evaporated. The obtained crude product was purified via flash-chromatography (Interchim devices puriFLASH 5.125) on silica gel (PF-15SIHP-F0040) using cyclohexane/ethyl acetate 0% to 20% ethyl acetate in 20 column volumes (1 column volume = 91.9 mL; flow: 26 mL/min). The isolated product 2-(4-fluorophenyl)-6-(trifluoromethyl)imidazo[1,2-a]pyridine (293 mg, 1.04 mmol) was obtained as a light yellow solid in 85% yield. *R_f_* = 0.57 (cyclohexane/ethyl acetate 2:1).

^1^H NMR (400 MHz, Chloroform-d [7.27 ppm], ppm) δ = 8.49–8.48 (m, 1H), 7.96–7.91 (m, 2H), 7.89 (s, 1H), 7.72 (dd, *J* = 0.6 Hz, *J* = 9.4 Hz, 1H), 7.33 (dd, *J* = 1.8 Hz, *J* = 9.5 Hz, 1H), 7.18–7.12 (m, 2H); ^13^C NMR (100 MHz, Chloroform-d [77.0 ppm], ppm) δ = 163.0 (d, *J* = 248.1 Hz, C_q_), 146.9 (C_q_), 145.3 (C_q_), 129.1 (d, *J* = 3.9 Hz, C_q_), 127.9 (d, *J* = 8.5 Hz, 2C, CH), 124.5 (q, *J* = 5.8 Hz, CH), 123.5 (q, *J* = 271.0 Hz, CF_3_), 120.7 (q, *J* = 2.6 Hz, CH), 118.0 (CH), 117.0 (q, *J* = 34.2 Hz, C_q_), 115.8 (d, *J* = 21.6 Hz, 2C, CH), 108.8 (CH); ^19^F NMR (376 MHz, ppm) δ = -62.15 (CF3), -112.89 (CF); MS (EI, 70 eV, 40 °C), m/z (%): 280 (100) [M]^+^, HRMS–EI (C_14_H_8_N_2_F_4_) *(m/z)*: [M]^+^ Calcd 280.0618; Found 280.0619; IR (ATR, ṽ) = 1647 (w), 1611 (w), 1599 (w), 1550 (vw), 1489 (s), 1441 (w), 1417 (vw), 1383 (w), 1334 (vs), 1312 (vs), 1271 (w), 1234 (s), 1225 (s), 1200 (w), 1167 (vs), 1119 (vs), 1052 (vs), 1014 (w), 970 (w), 939 (m), 870 (m), 846 (vs), 822 (m), 806 (vs), 759 (m), 744 (vs), 708 (m), 670 (vs), 640 (s), 582 (w), 565 (w), 535 (m), 516 (vs) cm^–1^.

Additional information on the chemical synthesis is available via Chemotion repository:

<https://doi.org/10.14272/reaction/SA-FUHFF-UHFFFADPSC-RFCPFCDGJG-UHFFFADPSC-NUHFF-NUHFF-NUHFF-ZZZ>

Additional information on the analysis of the target compound is available via Chemotion repository:

<https://doi.org/10.14272/RFCPFCDGJGHFCX-UHFFFAOYSA-N.1>

**2-Phenylimidazo[1,2-a]pyridine** (**X19147**)

Name {X19147}: 2-phenylimidazo[1,2-a]pyridine; Formula: C_13_H_10_N_2_; CAS: - ; Molecular Mass: 194.2319; Exact Mass: 194.0844; EA: C, 80.39; H, 5.19; N, 14.42. Smiles: c1ccc(cc1)c1nc2n(c1)cccc2; InChIKey: KDHWCFCNNGUJCP-UHFFFAOYSA-N

A mixture of pyridin-2-amine (150 mg, 1.59 mmol, 1.00 equiv), 2-bromo-1-phenylethanone (317 mg, 1.59 mmol, 1.00 equiv) and sodium hydrogen carbonate (201 mg, 2.39 mmol, 1.50 equiv) in anhydrous toluene (4.00 mL) was stirred for 16 hours at 115 °C. As preparation for the column chromatography (dryload), Celite was added (0.9 g) and the reaction mixture with Celite was evaporated. The obtained crude product was purified via flash-chromatography (Interchim devices puriFLASH 5.125) on silica gel (PF-15SIHP-F0040) using cyclohexane/ethyl acetate 0% to 40% ethyl acetate in 15 column volumes (1 column volume = 91.9 mL; flow: 26 mL/min). The isolated product 2-phenylimidazo[1,2-a]pyridine (280 mg, 1.44 mmol) was obtained as a beige solid in 90% yield. *R_f_* = 0.30 (cyclohexane/ethyl acetate 2:1).

^1^H NMR (400 MHz, Chloroform-d [7.27 ppm], ppm) δ = 8.11 (pseudo-td, *J* = 1.2 Hz, *J* = 6.8 Hz, 1H), 7.98–7.95 (m, 2H), 7.86 (s, 1H), 7.66 (dd, *J* = 0.7 Hz, *J* = 9.1 Hz, 1H), 7.46–7.42 (m, 2H), 7.36–7.32 (m, 1H), 7.18 (ddd, *J* = 1.3 Hz, *J* = 6.8 Hz, *J* = 9.1 Hz, 1H), 6.78 (pseudo-dt, *J* = 1.1 Hz, *J* = 6.8 Hz, 1H); ^13^C NMR (100 MHz, Chloroform-d [77.0 ppm], ppm) δ = 145.6 (C_q_), 145.5 (C_q_), 133.5 (C_q_), 128.7 (2C, CH), 128.0 (CH), 126.0 (2C, CH), 125.6 (CH), 124.8 (CH), 117.4 (CH), 112.5 (CH), 108.1 (CH); MS (EI, 70 eV, 40 °C), m/z (%): 194 (100) [M]+, 78 (14). HRMS–EI (C_13_H_10_N_2_) *(m/z)*: [M]^+^ Calcd 194.0838; Found 194.0837; IR (ATR, ṽ) = 1632 (w), 1526 (w), 1503 (w), 1473 (m), 1443 (w), 1368 (m), 1351 (w), 1330 (w), 1305 (w), 1278 (w), 1269 (m), 1244 (w), 1201 (w), 1142 (w), 1128 (w), 1079 (w), 1068 (w), 1027 (w), 936 (w), 916 (w), 798 (vw), 781 (w), 751 (s), 737 (vs), 720 (vs), 693 (vs), 680 (s), 625 (w), 613 (w), 526 (w), 501 (s) cm^–1^.

Additional information on the chemical synthesis is available via Chemotion repository:

<https://doi.org/10.14272/reaction/SA-FUHFF-UHFFFADPSC-KDHWCFCNNG-UHFFFADPSC-NUHFF-NUHFF-NUHFF-ZZZ>

Additional information on the analysis of the target compound is available via Chemotion repository:

<https://doi.org/10.14272/KDHWCFCNNGUJCP-UHFFFAOYSA-N.1>

**2-(2-Fluorophenyl)imidazo[1,2-a]pyridine** (**X19712**)

Name {X19712}: 2-(2-fluorophenyl)imidazo[1,2-a]pyridine; Formula: C_13_H_9_FN_2_; CAS: - ; Molecular Mass: 212.2224; Exact Mass: 212.0750; EA: C, 73.57; F, 8.95; H, 4.27; N, 13.2. Smiles: Fc1ccccc1c1nc2n(c1)cccc2; InChIKey: SANQKCXADXGKQK-UHFFFAOYSA-N

A mixture of pyridin-2-amine (150 mg, 1.59 mmol, 1.00 equiv), 2-bromo-1-(2-fluorophenyl)ethanone (346 mg, 1.59 mmol, 1.00 equiv) and sodium hydrogen carbonate (201 mg, 2.39 mmol, 1.50 equiv) in anhydrous toluene (4.00 mL) was stirred for 16 hours at 115 °C. As preparation for the column chromatography (dry load), Celite was added (0.9 g) and the reaction mixture with Celite was evaporated. The obtained crude product was purified via flash-chromatography (Interchim devices puriFLASH 5.125) on silica gel (PF-15SIHP-F0040) using cyclohexane/ethyl acetate with 0% to 25% ethyl acetate in 15 column volumes (1 column volume = 91.9 mL; flow: 26 mL/min). The isolated product 2-(2-fluorophenyl)imidazo[1,2-a]pyridine (311 mg, 1.47 mmol) was obtained as an off-white solid in 92% yield. *R_f_* = 0.33 (cyclohexane/ethyl acetate 2:1).

^1^H NMR (400 MHz, Chloroform-d [7.27 ppm], ppm) δ = 8.40–8.35 (m, 1H), 8.14 (dt, *J* = 6.8, 1.2 Hz, 1H), 8.06 (d, *J* = 3.9 Hz, 1H), 7.66 (dd, *J* = 0.7 Hz, *J* = 9.1 Hz, 1H), 7.33–7.25 (m, 2H), 7.22–7.13 (m, 2H), 6.79 6.79 (td, *J* = 6.8, 1.2 Hz, 1H); ^13^C NMR (100 MHz, Chloroform-d [77.0 ppm], ppm) δ = 160.3 (d, *J* = 248.9 Hz, C_q_), 144.8 (C_q_), 139.1 (d, *J* = 1.5 Hz, C_q_), 129.0 (d, *J* = 8.5 Hz, CH), 128.8 (d, *J* = 3.9 Hz, CH), 125.7 (CH), 125.0 (CH), 124.5 (d, *J* = 3.9 Hz, CH), 121.4 (d, *J* = 12.3 Hz, C_q_), 117.4 (CH), 115.6 (d, *J* = 21.6 Hz, CH), 112.5 (CH), 112.1 (d, *J* = 15.4 Hz, CH); ^19^F NMR (376 MHz, ppm) δ = -114.01; MS (EI, 70 eV, 30 °C), m/z (%): 212 (100) [M]^+^, 78 (11). HRMS–EI (C_13_H_9_N_2_F) *(m/z)*: [M]^+^ Calcd 212.0744; Found 212.0743; IR (ATR, ṽ) = 3162 (w), 3051 (w), 1633 (w), 1581 (w), 1545 (w), 1500 (w), 1482 (s), 1456 (w), 1438 (w), 1368 (w), 1356 (w), 1322 (w), 1276 (w), 1256 (w), 1245 (w), 1210 (s), 1194 (w), 1157 (w), 1143 (w), 1129 (w), 1105 (w), 1071 (m), 1031 (w), 1017 (w), 1000 (w), 941 (w), 915 (w), 826 (m), 789 (w), 768 (w), 737 (vs), 715 (vs), 674 (m), 662 (m), 623 (w), 584 (w), 560 (w), 545 (w), 521 (w) cm^–1^.

Additional information on the chemical synthesis is available via Chemotion repository:

<https://doi.org/10.14272/reaction/SA-FUHFF-UHFFFADPSC-SANQKCXADX-UHFFFADPSC-NUHFF-NUHFF-NUHFF-ZZZ>

Additional information on the analysis of the target compound is available via Chemotion repository:

<https://doi.org/10.14272/SANQKCXADXGKQK-UHFFFAOYSA-N.1>

**2-(2-Fluorophenyl)-8-methyl-imidazo[1,2-a]pyridine** (**X19718**)

Name {X19718}: 2-(2-fluorophenyl)-8-methyl-imidazo[1,2-a]pyridine; Formula: C_14_H_11_FN_2_; CAS: - ; Molecular Mass: 226.2489; Exact Mass: 226.0906; EA: C, 74.32; F, 8.4; H, 4.9; N, 12.38. Smiles: Fc1ccccc1c1cn2c(n1)c(C)ccc2; InChIKey: HFJDLAHRMQHDEB-UHFFFAOYSA-N

A mixture of 3-methylpyridin-2-amine (150 mg, 1.39 mmol, 1.00 equiv), 2-bromo-1-(2-fluorophenyl)ethanone (301 mg, 1.39 mmol, 1.00 equiv) and sodium hydrogen carbonate (175 mg, 2.08 mmol, 1.50 equiv) in anhydrous toluene (4.00 mL) was stirred for 16 hours at 115 °C. As preparation for the column chromatography (dry load), Celite was added (0.9 g) and the reaction mixture with Celite was evaporated. The obtained crude product was purified via flash-chromatography (Interchim devices puriFLASH 5.125) on silica gel (PF-15SIHP-F0040) using cyclohexane/ethyl acetate with 0% to 25% ethyl acetate in 15 column volumes (1 column volume = 91.9 mL; flow: 26 mL/min). The isolated product 2-(2-fluorophenyl)-8-methyl-imidazo[1,2-a]pyridine (270 mg, 1.19 mmol) was obtained as a light orange solid in 86% yield. *R_f_* = 0.63 (cyclohexane/ethyl acetate 2:1).

^1^H NMR (400 MHz, Chloroform-d [7.27 ppm], ppm) δ = 8.46–8.41 (m, 1H), 8.04 (d, *J* = 4.0 Hz, 1H), 8.00 (dd, *J* = 0.4 Hz, *J* = 6.7 Hz, 1H), 7.32–7.25 (m, 2H), 7.19–7.11 (m, 1H), 6.97 (td, *J* = 1.1 Hz, *J* = 6.8 Hz, 1H), 6.69 (t, *J* = 6.8 Hz, 1H), 2.68 (s, 3H); ^13^C NMR (100 MHz, Chloroform-d [77.0 ppm], ppm) δ = 160.2 (d, *J* = 248.9 Hz, C_q_), 145.3 (C_q_), 138.4 (d, *J* = 2.3 Hz, C_q_), 129.1 (d, *J* = 3.9 Hz, CH), 128.7 (d, *J* = 8.5 Hz, CH), 127.5 (C_q_), 124.5 (CH), 124.4 (CH), 123.5 (CH), 121.7 (d, *J* = 13.1 Hz, C_q_), 115.5 (d, *J* = 21.6 Hz, CH), 112.6 (CH), 112.4 (CH), 17.0 (CH_3_); ^19^F NMR (376 MHz, ppm) δ = -114.35; MS (EI, 70 eV, 30 °C), m/z (%): 226 (100) [M]^+^,. HRMS–EI (C_14_H_11_N_2_F) *(m/z)*: [M]^+^ Calcd 226.0901; Found 226.0901; IR (ATR, ṽ) = 3176 (vw), 2952 (vw), 2918 (vw), 1629 (w), 1609 (vw), 1572 (w), 1547 (vw), 1493 (w), 1480 (s), 1453 (w), 1441 (w), 1422 (w), 1377 (w), 1366 (m), 1347 (w), 1322 (w), 1269 (w), 1256 (w), 1208 (s), 1196 (m), 1164 (w), 1157 (w), 1111 (w), 1071 (m), 1037 (w), 1028 (w), 994 (vw), 942 (w), 878 (w), 864 (w), 816 (w), 764 (s), 741 (vs), 711 (vs), 671 (m), 653 (w), 640 (m), 608 (w), 592 (w), 567 (w), 540 (m), 511 (w), 504 (w) cm^–1^.

Additional information on the chemical synthesis is available via Chemotion repository:

<https://doi.org/10.14272/reaction/SA-FUHFF-UHFFFADPSC-HFJDLAHRMQ-UHFFFADPSC-NUHFF-NUHFF-NUHFF-ZZZ>

Additional information on the analysis of the target compound is available via Chemotion repository:

<https://doi.org/10.14272/HFJDLAHRMQHDEB-UHFFFAOYSA-N.1>

**2-(4-Fluorophenyl)-8-methyl-imidazo[1,2-a]pyridine** (**X19167**)

Name {X19167}: 2-(4-fluorophenyl)-8-methyl-imidazo[1,2-a]pyridine; Formula: C_14_H_11_FN_2_; CAS: - ; Molecular Mass: 226.2489; Exact Mass: 226.0906; EA: C, 74.32; F, 8.4; H, 4.9; N, 12.38. Smiles: Fc1ccc(cc1)c1cn2c(n1)c(C)ccc2; InChIKey: UDBRHPCMIXCPEW-UHFFFAOYSA-N

A mixture of 3-methylpyridin-2-amine (150 mg, 1.39 mmol, 1.00 equiv), 2-bromo-1-(4-fluorophenyl)ethanone (301 mg, 1.39 mmol, 1.00 equiv) and sodium hydrogen carbonate (175 mg, 2.08 mmol, 1.50 equiv) in anhydrous toluene (4.00 mL) was stirred for 16 hours at 115 °C.

As preparation for the column chromatography (dryload), Celite was added (0.9 g) and the reaction mixture with Celite was evaporated. The obtained crude product was purified via flash-chromatography (Interchim devices puriFLASH 5.125) on silica gel (PF-15SIHP-F0040) using cyclohexane/ethyl acetate 0% to 33% ethyl acetate in 20 column volumes (1 column volume = 91.9 mL; flow: 26 mL/min). The isolated product 2-(4-fluorophenyl)-8-methyl-imidazo[1,2-a]pyridine (282 mg, 1.24 mmol) was obtained as a colorless solid in 90% yield. *R_f_* = 0.35 (cyclohexane/ethyl acetate 2:1).

^1^H NMR (400 MHz, Chloroform-d [7.27 ppm], ppm) δ = 7.97–7.92 (m, 3H), 7.76 (s, 1H), 7.15–7.09 (m, 2H), 6.95 (td, *J* = 1.2 Hz, *J* = 6.8 Hz, 1H), 6.68 (pseudo-t, *J* = 6.8 Hz, 1H), 2.66 (s, 3H); ^13^C NMR (100 MHz, Chloroform-d [77.0 ppm], ppm) δ = 162.6 (d, *J* = 246.6 Hz, C_q_), 146.1 (C_q_), 144.3 (C_q_), 130.3 (d, *J* = 3.1 Hz, C_q_), 127.8 (d, *J* = 8.5 Hz, 2C, CH), 127.5 (C_q_), 123.4 (CH), 123.3 (CH), 115.5 (d, *J* = 21.6 Hz, 2C, CH), 112.4 (CH), 108.2 (CH), 17.0 (CH_3_); ^19^F NMR (376 MHz, ppm) δ = -114.53; MS (EI, 70 eV, 40 °C), m/z (%): 226 (100) [M]+, HRMS–EI (C_14_H_11_N_2_F) *(m/z)*: [M]^+^ Calcd 226.0901; Found 226.0899; IR (ATR, ṽ) = 2925 (w), 1667 (w), 1629 (w), 1596 (w), 1550 (w), 1499 (w), 1482 (vs), 1443 (w), 1431 (w), 1412 (w), 1378 (m), 1367 (m), 1343 (w), 1319 (w), 1292 (w), 1282 (w), 1261 (w), 1254 (w), 1235 (w), 1213 (vs), 1152 (s), 1101 (m), 1077 (m), 1057 (w), 1033 (w), 1013 (w), 993 (w), 966 (w), 938 (m), 880 (w), 849 (vs), 824 (s), 809 (s), 778 (w), 765 (s), 744 (vs), 720 (s), 701 (vs), 676 (m), 635 (m), 613 (m), 594 (w), 577 (vs), 548 (m), 527 (vs), 503 (s) cm^–1^.

Additional information on the chemical synthesis is available via Chemotion repository:

<https://doi.org/10.14272/reaction/SA-FUHFF-UHFFFADPSC-UDBRHPCMIX-UHFFFADPSC-NUHFF-NUHFF-NUHFF-ZZZ>

Additional information on the analysis of the target compound is available via Chemotion repository:

<https://doi.org/10.14272/UDBRHPCMIXCPEW-UHFFFAOYSA-N.1>

**8-Chloro-2-phenyl-imidazo[1,2-a]pyridine** (**X19726**)

Name {X19726}: 8-chloro-2-phenyl-imidazo[1,2-a]pyridine; Formula: C_13_H_9_ClN_2_; CAS: - ; Molecular Mass: 228.6770; Exact Mass: 228.0454; EA: C, 68.28; Cl, 15.5; H, 3.97; N, 12.25.

Smiles: Clc1cccn2c1nc(c2)c1ccccc1

InChIKey: GSUJVBYDVUYTAD-UHFFFAOYSA-N

A mixture of 3-chloropyridin-2-amine (150 mg, 1.17 mmol, 1.00 equiv), 2-bromo-1-phenylethanone (232 mg, 1.17 mmol, 1.00 equiv) and sodium hydrogen carbonate (147 mg, 1.75 mmol, 1.50 equiv) in anhydrous toluene (6.00 mL) was stirred for 72 hours at 115 °C. As preparation for the column chromatography (dry load), Celite was added (0.9 g) and the reaction mixture with Celite was evaporated. The obtained crude product was purified via flash-chromatography (Interchim devices puriFLASH 5.125) on silica gel (PF-15SIHP-F0040) using cyclohexane/ethyl acetate with 0% to 25% ethyl acetate in 15 column volumes (1 column volume = 91.9 mL; flow: 26 mL/min). The isolated product 8-chloro-2-phenyl-imidazo[1,2-a]pyridine (260 mg, 1.14 mmol) was obtained as an off-white solid in 97% yield. *R_f_* = 0.43 (cyclohexane/ethyl acetate 2:1).

^1^H NMR (400 MHz, Chloroform-d [7.27 ppm], ppm) δ = 8.04 (dd, *J* = 1.0 Hz, *J* = 6.7 Hz, 1H), 8.00–7.98 (m, 2H), 7.89 (s, 1H), 7.46–7.42 (m, 2H), 7.37–7.32 (m, 1H), 7.23 (dd, *J* = 1.0 Hz, *J* = 7.3 Hz, 1H), 6.70 (t, *J* = 7.0 Hz, 1H); ^13^C NMR (100 MHz, Chloroform-d [77.0 ppm], ppm) δ = 146.3 (C_q_), 143.0 (C_q_), 133.1 (C_q_), 128.6 (CH, 2C), 128.2 (CH), 126.3 (CH, 2C), 124.3 (CH), 123.6 (CH), 123.2 (C_q_), 112.0 (CH), 109.6 (CH); MS (EI, 70 eV, 70 °C), m/z (%): 228/230 (100/36) [M]^+^,. HRMS–EI (C_13_H_9_N_2_Cl) *(m/z)*: [M]^+^ Calcd 228.0449; Found 228.0447; IR (ATR, ṽ) = 3135 (w), 3029 (w), 1628 (w), 1520 (w), 1497 (w), 1473 (m), 1442 (w), 1366 (m), 1341 (w), 1332 (w), 1302 (w), 1252 (m), 1215 (w), 1198 (m), 1146 (m), 1079 (w), 1071 (w), 975 (m), 932 (m), 916 (m), 868 (w), 851 (w), 764 (vs), 722 (vs), 713 (vs), 691 (vs), 633 (m), 619 (w), 602 (w), 557 (w), 540 (w), 527 (s) cm^–1^.

Additional information on the chemical synthesis is available via Chemotion repository:

<https://doi.org/10.14272/reaction/SA-FUHFF-UHFFFADPSC-GSUJVBYDVU-UHFFFADPSC-NUHFF-NUHFF-NUHFF-ZZZ>

Additional information on the analysis of the target compound is available via Chemotion repository:

<https://doi.org/10.14272/GSUJVBYDVUYTAD-UHFFFAOYSA-N.1>

**8-Chloro-2-(2-fluorophenyl)imidazo[1,2-a]pyridine** (**X19727**)

Name {X19727}: 8-chloro-2-(2-fluorophenyl)imidazo[1,2-a]pyridine; Formula: C_13_H_8_ClFN_2_; CAS: - ; Molecular Mass: 246.6674; Exact Mass: 246.0360; EA: C, 63.3; Cl, 14.37; F, 7.7; H, 3.27; N, 11.36. Smiles: Fc1ccccc1c1cn2c(n1)c(Cl)ccc2; InChIKey: RITJJKBOMYWBLG-UHFFFAOYSA-N

A mixture of 3-chloropyridin-2-amine (150 mg, 1.17 mmol, 1.00 equiv), 2-bromo-1-(2-fluorophenyl)ethanone (253 mg, 161 μL, 1.17 mmol, 1.00 equiv) and sodium hydrogen carbonate (147 mg, 1.75 mmol, 1.50 equiv) in anhydrous toluene (6.00 mL) was stirred for 72 hours at 115 °C. As preparation for the column chromatography (dryload), Celite was added (0.9 g) and the reaction mixture with Celite were evaporated. The obtained crude product was purified via flash-chromatography (Interchim devices puriFLASH 5.125) on silica gel (PF-15SIHP-F0040) using cyclohexane/ethyl acetate 0% to 25% ethyl acetate in 20 column volumes (1 column volume = 91.9 mL; flow: 26 mL/min). The isolated product 8-chloro-2-(2-fluorophenyl)imidazo[1,2-a]pyridine (284 mg, 1.15 mmol) was obtained as a off-white solid in 99% yield. *R_f_* = 0.57 (cyclohexane/ethyl acetate 2:1).

^1^H NMR (400 MHz, Chloroform-d [7.27 ppm], ppm) δ = 8.46 (td, *J* = 2.1 Hz, *J* = 7.7 Hz, 1H), 8.10 (d, *J* = 3.9 Hz, 1H), 8.07 (dd, *J* = 1.0 Hz, *J* = 6.7 Hz, 1H), 7.34–7.22 (m, 3H), 7.17–7.12 (m, 1H), 6.72 (t, *J* = 7.0 Hz, 1H); ^13^C NMR (100 MHz, Chloroform-d [77.0 ppm], ppm) δ = 160.3 (d, *J* = 249.7 Hz, C_q_), 142.2 (C_q_), 139.8 (d, *J* = 1.5 Hz, C_q_), 129.3 (d, *J* = 4.6 Hz, CH), 129.3 (d, *J* = 16.2 Hz, CH), 124.5 (CH), 124.4 (CH), 123.8 (CH), 123.2 (C_q_), 121.0 (d, *J* = 11.6 Hz, C_q_), 115.5 (d, *J* = 21.6 Hz, CH), 113.5 (d, *J* = 15.4 Hz, CH), 112.0 (CH); ^19^F NMR (376 MHz, ppm) δ = -114.12; MS (EI, 70 eV, 70 °C), m/z (%): 246/248 (100/34) [M]^+^,. HRMS–EI (C_13_H_8_N_2_ClF) *(m/z)*: [M]^+^ Calcd 246.0355; Found 246.0354; IR (ATR, ṽ) = 3174 (w), 3101 (w), 3091 (w), 3029 (w), 2925 (w), 1807 (vw), 1748 (vw), 1628 (w), 1577 (vw), 1550 (w), 1523 (w), 1496 (w), 1480 (s), 1439 (m), 1364 (s), 1339 (w), 1324 (w), 1264 (m), 1249 (w), 1215 (m), 1205 (m), 1197 (s), 1160 (w), 1145 (w), 1111 (w), 1071 (m), 1050 (w), 1028 (w), 976 (m), 946 (w), 932 (m), 875 (m), 813 (w), 764 (vs), 755 (vs), 732 (vs), 720 (vs), 696 (vs), 673 (m), 632 (m), 564 (w), 545 (w), 530 (m), 520 (s) cm^–1^.

Additional information on the chemical synthesis is available via Chemotion repository:

<https://doi.org/10.14272/reaction/SA-FUHFF-UHFFFADPSC-RITJJKBOMY-UHFFFADPSC-NUHFF-NUHFF-NUHFF-ZZZ>

Additional information on the analysis of the target compound is available via Chemotion repository:

<https://doi.org/10.14272/RITJJKBOMYWBLG-UHFFFAOYSA-N.1>

**8-Chloro-2-(4-fluorophenyl)imidazo[1,2-a]pyridine** (**X19725**)

Name {X19725}: 8-chloro-2-(4-fluorophenyl)imidazo[1,2-a]pyridine; Formula: C_13_H_8_ClFN_2_; CAS: - ; Molecular Mass: 246.6674; Exact Mass: 246.0360; EA: C, 63.3; Cl, 14.37; F, 7.7; H, 3.27; N, 11.36. Smiles: Fc1ccc(cc1)c1cn2c(n1)c(Cl)ccc2; InChIKey: PGCCRJVQXFSFNG-UHFFFAOYSA-N

A mixture of 3-chloropyridin-2-amine (150 mg, 1.17 mmol, 1.00 equiv), 2-bromo-1-(4-fluorophenyl)ethanone (253 mg, 1.17 mmol, 1.00 equiv) and sodium hydrogen carbonate (147 mg, 1.75 mmol, 1.50 equiv) in anhydrous toluene (6.00 mL) was stirred for 72 hours at 115 °C. As preparation for the column chromatography (dryload), Celite was added (0.9 g) and the reaction mixture with Celite was evaporated. The obtained crude product was purified via flash-chromatography (Interchim devices puriFLASH 5.125) on silica gel (PF-15SIHP-F0040) using cyclohexane/ethyl acetate 0% to 25% ethyl acetate in 15 column volumes (1 column volume = 91.9 mL; flow: 26 mL/min). The isolated product 8-chloro-2-(4-fluorophenyl)imidazo[1,2-a]pyridine (174 mg, 705 μmol) was obtained as an off-white solid in 60% yield. *R_f_* = 0.42 (cyclohexane/ethyl acetate 2:1).

^1^H NMR (400 MHz, Chloroform-d [7.27 ppm], ppm) δ = 8.05 (dd, *J* = 1.0 Hz, *J* = 6.7 Hz, 1H), 7.98–7.93 (m, 2H), 7.85 (s, 1H), 7.25 (dd, *J* = 1.0 Hz, *J* = 7.3 Hz, 1H), 7.15–7.09 (m, 2H), 6.72 (dd, *J* = 7.4 Hz, 6.7 Hz, 1H); ^13^C NMR (100 MHz, Chloroform-d [77.0 ppm], ppm) δ = 162.9 (d, *J* = 247.4 Hz, C_q_), 145.5 (C_q_), 143.0 (C_q_), 129.4 (d, *J* = 3.1 Hz, C_q_), 128.0 (d, *J* = 8.5 Hz, 2C, CH), 124.3 (CH), 123.7 (CH), 123.2 (C_q_), 115.6 (d, *J* = 22.3 Hz, 2C, CH), 112.1 (CH), 109.3 (CH); ^19^F NMR (376 MHz, ppm) δ = -113.62; MS (EI, 70 eV, 70 °C), m/z (%): 246/248 (100/32) [M]^+^,. HRMS–EI (C_13_H_8_N_2_ClF) *(m/z)*: [M]^+^ Calcd 246.0355; Found 246.0356; IR (ATR, ṽ) = 3135 (m), 3089 (w), 3044 (w), 1924 (w), 1900 (w), 1874 (w), 1816 (w), 1628 (w), 1595 (m), 1520 (m), 1494 (w), 1479 (s), 1439 (m), 1411 (m), 1364 (s), 1337 (m), 1329 (m), 1319 (m), 1292 (m), 1256 (m), 1235 (w), 1210 (vs), 1200 (vs), 1146 (s), 1096 (s), 1077 (s), 1050 (m), 1011 (w), 980 (vs), 962 (m), 932 (vs), 871 (s), 849 (vs), 820 (s), 806 (s), 769 (vs), 739 (vs), 704 (vs), 684 (vs), 635 (m), 613 (m), 564 (s), 557 (m), 540 (s), 530 (vs), 510 (s) cm^–1^.

Additional information on the chemical synthesis is available via Chemotion repository:

<https://doi.org/10.14272/reaction/SA-FUHFF-UHFFFADPSC-PGCCRJVQXF-UHFFFADPSC-NUHFF-NUHFF-NUHFF-ZZZ>

Additional information on the analysis of the target compound is available via Chemotion repository:

<https://doi.org/10.14272/PGCCRJVQXFSFNG-UHFFFAOYSA-N.1>

**6-Bromo-2-phenyl-imidazo[1,2-a]pyridine** (**X19148**)

Name {X19148}: 6-bromo-2-phenyl-imidazo[1,2-a]pyridine; Formula: C_13_H_9_BrN_2_; CAS: - ; Molecular Mass: 273.1280; Exact Mass: 271.9949; EA: Br, 29.26; C, 57.17; H, 3.32; N, 10.26. Smiles: Brc1ccc2n(c1)cc(n2)c1ccccc1; InChIKey: JECUQJPFGTUNCJ-UHFFFAOYSA-N

A mixture of 5-bromopyridin-2-amine (150 mg, 867 μmol, 1.00 equiv), 2-bromo-1-phenylethanone (173 mg, 867 μmol, 1.00 equiv) and sodium hydrogen carbonate (109 mg, 1.30 mmol, 1.50 equiv) in anhydrous toluene (7.00 mL) was stirred for 16 hours at 115°C. As preparation for the column chromatography (dryload), Celite was added (0.9 g) and the reaction mixture with Celite was evaporated. The obtained crude product was purified via flash-chromatography (Interchim devices puriFLASH 5.125) on silica gel (PF-15SIHP-F0040) using cyclohexane/ethyl acetate 0% to 30% ethyl acetate in 20 column volumes (1 column volume = 91.9 mL; flow: 26 mL/min). The isolated product 6-bromo-2-phenyl-imidazo[1,2-a]pyridine (105 mg, 384 μmol) was obtained as a colorless solid in 44% yield. *R_f_* = 0.56 (cyclohexane/ethyl acetate 2:1).

^1^H NMR (400 MHz, Chloroform-d [7.27 ppm], ppm) δ = 8.25 (dd, *J* = 0.7 Hz, *J* = 1.8 Hz, 1H), 7.95–7.92 (m, 2H), 7.81 (s, 1H), 7.54 (d, *J* = 9.5 Hz, 1H), 7.46–7.43 (m, 2H), 7.38–7.33 (m, 1H), 7.24 (dd, *J* = 2.0 Hz, *J* = 9.5 Hz, 1H); ^13^C NMR (100 MHz, Chloroform-d [77.0 ppm], ppm) δ = 146.5 (C_q_), 144.0 (C_q_), 133.0 (C_q_), 128.8 (2C, CH), 128.3 (CH), 128.2 (CH), 126.1 (2C, CH), 125.5 (CH), 118.0 (CH), 108.2 (CH), 107.0 (C_q_); MS (EI, 70 eV, 60 °C), m/z (%): 272/274 (100/96) [M]+, 208 (12), 192 (15), 105 (52), 91 (15), 89 (10), 77 (29), 71 (15), 69 (10), 63 (12), 58 (48), 55 (13). HRMS–EI (C_13_H_9_N_2_Br) *(m/z)*: [M]^+^ Calcd 271.9944; Found 271.9942; IR (ATR, ṽ) = 3129 (w), 3084 (w), 3055 (w), 3026 (w), 2990 (w), 1676 (w), 1509 (w), 1496 (w), 1473 (w), 1446 (w), 1421 (w), 1390 (w), 1366 (w), 1339 (w), 1327 (w), 1268 (w), 1238 (w), 1207 (w), 1177 (w), 1153 (w), 1137 (w), 1082 (w), 1060 (w), 1026 (w), 1000 (w), 980 (w), 965 (w), 939 (w), 926 (w), 911 (w), 854 (w), 841 (w), 806 (vs), 772 (s), 741 (w), 715 (vs), 683 (vs), 619 (w), 596 (w), 567 (m), 547 (w), 530 (w), 504 (s) cm^–1^.

Additional information on the chemical synthesis is available via Chemotion repository:

<https://doi.org/10.14272/reaction/SA-FUHFF-UHFFFADPSC-JECUQJPFGT-UHFFFADPSC-NUHFF-NUHFF-NUHFF-ZZZ>

Additional information on the analysis of the target compound is available via Chemotion repository:

<https://doi.org/10.14272/JECUQJPFGTUNCJ-UHFFFAOYSA-N.1>

**6-Bromo-2-(2-fluorophenyl)imidazo[1,2-a]pyridine** (**X19719**)

Name {X19719}: 6-bromo-2-(2-fluorophenyl)imidazo[1,2-a]pyridine; Formula: C_13_H_8_BrFN_2_; CAS: - ; Molecular Mass: 291.1184; Exact Mass: 289.9855; EA: Br, 27.45; C, 53.63; F, 6.53; H, 2.77; N, 9.62. Smiles: Brc1ccc2n(c1)cc(n2)c1ccccc1F; InChIKey: WIERVJWCSZUKSW-UHFFFAOYSA-N

A mixture of 5-bromopyridin-2-amine (150 mg, 867 μmol, 1.00 equiv), 2-bromo-1-(2-fluorophenyl)ethanone (188 mg, 867 μmol, 1.00 equiv) and sodium hydrogen carbonate (109 mg, 1.30 mmol, 1.50 equiv) in anhydrous toluene (7.00 mL) was stirred for 16 hours at 115 °C. As preparation for the column chromatography (dryload), Celite was added (0.9 g) and the reaction mixture with Celite was evaporated. The obtained crude product was purified via flash-chromatography (Interchim devices puriFLASH 5.125) on silica gel (PF-15SIHP-F0040) using cyclohexane/ethyl acetate 0% to 10% ethyl acetate in 20 column volumes (1 column volume = 91.9 mL; flow: 26 mL/min). The isolated product 6-bromo-2-(2-fluorophenyl)imidazo[1,2-a]pyridine (184 mg, 631 μmol) was obtained as a colorless solid in 73% yield. *R_f_* = 0.55 (cyclohexane/ethyl acetate 2:1).

^1^H NMR (400 MHz, Chloroform-d [7.27 ppm], ppm) δ = 8.33 (td, *J* = 2.1 Hz, *J* = 7.6 Hz, 1H), 8.28 (dd, *J* = 0.7 Hz, *J* = 1.8 Hz, 1H), 8.01 (dd, *J* = 3.9, *J* = 0.6 Hz, 1H), 7.54 (dd, *J* = 9.5, *J* = 0.9 Hz, 1H), 7.33–7.24 (m, 3H), 7.15 (ddd, *J* = 11.5, *J* = 7.9, *J* = 1.5 Hz, 1H); ^13^C NMR (100 MHz, Chloroform-d [77.0 ppm], ppm) δ = 160.3 (d, *J* = 248.9 Hz, C_q_), 143.2 (C_q_), 139.9 (d, *J* = 2.3 Hz, C_q_), 129.3 (d, *J* = 9.2 Hz, CH), 128.8 (d, *J* = 3.9 Hz, CH), 128.5 (CH), 125.7 (CH), 124.5 (d, *J* = 3.9 Hz, CH), 120.9 (d, *J* = 12.3 Hz, C_q_), 118.0 (CH), 115.7 (d, *J* = 21.6 Hz, CH), 112.1 (d, *J* = 15.4 Hz, CH), 107.1 (C_q_); ^19^F NMR (376 MHz, ppm) δ = -113.78–-113.85 (m); MS (EI, 70 eV, 70 °C), m/z (%): 290/292 (100/94) [M]+, 210 (12), 63 (10), 58 (11). HRMS–EI (C_13_H_8_N_2_BrF) *(m/z)*: [M]^+^ Calcd 289.9849; Found 289.9847; IR (ATR, ṽ) = 1575 (w), 1540 (w), 1513 (w), 1476 (vs), 1441 (m), 1417 (s), 1358 (w), 1336 (s), 1261 (m), 1239 (m), 1208 (vs), 1196 (s), 1150 (w), 1105 (w), 1071 (m), 1057 (vs), 1027 (m), 952 (m), 938 (w), 928 (m), 871 (w), 846 (m), 832 (w), 796 (vs), 766 (vs), 745 (vs), 711 (vs), 686 (vs), 670 (vs), 660 (vs), 605 (m), 565 (s), 523 (vs) cm^–1^.

Additional information on the chemical synthesis is available via Chemotion repository:

<https://doi.org/10.14272/reaction/SA-FUHFF-UHFFFADPSC-WIERVJWCSZ-UHFFFADPSC-NUHFF-NUHFF-NUHFF-ZZZ>

Additional information on the analysis of the target compound is available via Chemotion repository:

<https://doi.org/10.14272/WIERVJWCSZUKSW-UHFFFAOYSA-N.1>

**6-Bromo-2-(4-fluorophenyl)imidazo[1,2-a]pyridine** (**X19168**)

Name {X19168}: 6-bromo-2-(4-fluorophenyl)imidazo[1,2-a]pyridine; Formula: C_13_H_8_BrFN_2_; CAS: - ; Molecular Mass: 291.1184; Exact Mass: 289.9855; EA: Br, 27.45; C, 53.63; F, 6.53; H, 2.77; N, 9.62. Smiles: Fc1ccc(cc1)c1cn2c(n1)ccc(c2)Br; InChIKey: NKIQEILZYPFNFA-UHFFFAOYSA-N

A mixture of 5-bromopyridin-2-amine (150 mg, 867 μmol, 1.00 equiv), 2-bromo-1-(4-fluorophenyl)ethanone (188 mg, 867 μmol, 1.00 equiv) and sodium hydrogen carbonate (109 mg, 1.30 mmol, 1.50 equiv) in anhydrous toluene (7.00 mL) was stirred for 16 hours at 115 °C. As preparation for the column chromatography (dryload), Celite was added (0.9 g) and the reaction mixture with Celite was evaporated. The obtained crude product was purified via flash-chromatography (Interchim devices puriFLASH 5.125) on silica gel (PF-15SIHP-F0040) using cyclohexane/ethyl acetate 0% to 30% ethyl acetate in 20 column volumes (1 column volume = 91.9 mL; flow: 26 mL/min). The isolated product 6-bromo-2-(4-fluorophenyl)imidazo[1,2-a]pyridine (147 mg, 505 μmol) was obtained as a colorless solid in 58% yield. *R_f_* = 0.49 (cyclohexane/ethyl acetate 2:1).

^1^H NMR (400 MHz, Chloroform-d [7.27 ppm], ppm) δ = 8.27 (dd, *J* = 0.9 Hz, *J* = 1.8 Hz, 1H), 7.94–7.89 (m, 2H), 7.77 (s, 1H), 7.54 (dd, *J* = 0.9 Hz, *J* = 9.4 Hz, 1H), 7.26 (dd, 1.9 Hz, *J* = 9.5 Hz, 1H), 7.16–7.10 (m, 2H); ^13^C NMR (100 MHz, Chloroform-d [77.0 ppm], ppm) δ = 162.9 (d, *J* = 247.4 Hz, C_q_), 145.5 (C_q_), 143.9 (C_q_), 129.2 (d, *J* = 3.1 Hz, C_q_), 128.4 (CH), 127.8 (d, *J* = 7.7 Hz, 2C, CH), 125.6 (CH), 117.9 (CH), 115.8 (d, *J* = 21.6 Hz, 2C, CH), 107.9 (C_q_), 107.2 (CH); ^19^F NMR (376 MHz, ppm) δ = -113.27; MS (EI, 70 eV, 70 °C), m/z (%): 290/292 (100/99) [M]+, 210 (15), 123 (12), 63 (11), 58 (13). HRMS–EI (C_13_H_8_N_2_BrF) *(m/z)*: [M]^+^ Calcd 289.9849; Found 289.9848; IR (ATR, ṽ) = 3132 (vw), 1596 (w), 1544 (vw), 1519 (w), 1477 (w), 1419 (w), 1407 (w), 1334 (w), 1315 (w), 1293 (w), 1256 (w), 1232 (w), 1214 (w), 1200 (w), 1150 (w), 1137 (w), 1095 (m), 1077 (w), 1052 (m), 1007 (w), 960 (w), 946 (w), 936 (w), 926 (w), 841 (s), 832 (vs), 795 (vs), 737 (vs), 707 (vs), 686 (m), 676 (m), 637 (m), 598 (w), 568 (m), 518 (vs) cm^–1^.

Additional information on the chemical synthesis is available via Chemotion repository:

<https://doi.org/10.14272/reaction/SA-FUHFF-UHFFFADPSC-NKIQEILZYP-UHFFFADPSC-NUHFF-NUHFF-NUHFF-ZZZ>

Additional information on the analysis of the target compound is available via Chemotion repository:

<https://doi.org/10.14272/NKIQEILZYPFNFA-UHFFFAOYSA-N.1>

**6-Methyl-2-phenyl-imidazo[1,2-a]pyridine** (**X20034**)

Name {X20034}: 6-methyl-2-phenyl-imidazo[1,2-a]pyridine; Formula: C_14_H_12_N_2_; CAS: - ; Molecular Mass: 208.2585; Exact Mass: 208.1000; EA: C, 80.74; H, 5.81; N, 13.45. Smiles: Cc1ccc2n(c1)cc(n2)c1ccccc1; InChIKey: GYOZQHDHGJDZIQ-UHFFFAOYSA-N

A mixture of 5-methylpyridin-2-amine (0.150 g, 1.39 mmol, 1.00 equiv), 2-bromo-1-phenylethanone (0.276 g, 1.39 mmol, 1.000 equiv) and sodium hydrogen carbonate (0.175 g, 2.08 mmol, 1.50 equiv) in anhydrous toluene (6.00 mL) was stirred for 16 hours at 115 °C. As preparation for column chromatography (dry load), Celite was added (0.9 g) and the reaction mixture with Celite was evaporated. The obtained crude product was purified via flash-chromatography (Interchim devices puriFLASH 5.125) on silica gel (PF-15SIHP-F0040) using cyclohexane/ethyl acetate with 0% to 20% ethyl acetate in 15 column volumes (1 column volume = 91.9 mL; flow: 26 mL/min). The isolated product 6-methyl-2-phenyl-imidazo[1,2-a]pyridine (247 mg, 1.19 mmol) was obtained as a light orange solid in 86% yield. *R_f_* = 0.25 (cyclohexane/ethyl acetate 2:1).

^1^H NMR (400 MHz, Chloroform-d [7.27 ppm], ppm) δ = 7.95–7.92 (m, 2H), 7.82 (dt, *J* = 1.9, 1.1 Hz, 1H), 7.71 (d, *J* = 0.5 Hz, 1H), 7.52 (d, *J* = 9.3 Hz, 1H), 7.44–7.40 (m, 2H), 7.33–7.29 (m, 1H), 6.99 (dd, *J* = 1.7 Hz, *J* = 9.0 Hz, 1H), 2.27 (d, *J* = 1.2 Hz, 3H); ^13^C NMR (100 MHz, Chloroform-d [77.0 ppm], ppm) δ = 145.4 (C_q_), 144.6 (C_q_), 133.8 (C_q_), 128.6 (CH, 2C), 127.7 (CH), 127.7 (CH), 125.8 (CH, 2C), 123.2 (CH), 121.9 (C_q_), 116.7 (CH), 107.8 (CH), 18.0 (CH_3_); MS (EI, 70 eV, 60 °C), m/z (%): 208 (100) [M]^+^, 77 (11), 69 (12), 58 (39). HRMS–EI (C_14_H_12_N_2_) *(m/z)*: [M]^+^ Calcd 208.0995; Found 208.0997; IR (ATR, ṽ) = 3036 (w), 1602 (vw), 1531 (w), 1507 (w), 1475 (w), 1448 (w), 1419 (w), 1344 (w), 1334 (w), 1286 (w), 1272 (w), 1256 (w), 1208 (w), 1177 (vw), 1162 (w), 1081 (w), 1067 (w), 1041 (w), 1027 (w), 936 (w), 909 (w), 803 (vs), 768 (m), 748 (w), 711 (vs), 687 (vs), 620 (w), 611 (w), 572 (w), 534 (w), 507 (m) cm^–1^.

Additional information on the chemical synthesis is available via Chemotion repository:

<https://doi.org/10.14272/reaction/SA-FUHFF-UHFFFADPSC-GYOZQHDHGJ-UHFFFADPSC-NUHFF-NUHFF-NUHFF-ZZZ>

Additional information on the analysis of the target compound is available via Chemotion repository:

<https://doi.org/10.14272/GYOZQHDHGJDZIQ-UHFFFAOYSA-N.1>

**2-(2-Fluorophenyl)-6-methyl-imidazo[1,2-a]pyridine** (**X20035**)

Name {X20035}: 2-(2-fluorophenyl)-6-methyl-imidazo[1,2-a]pyridine; Formula: C_14_H_11_FN_2_; CAS: - ; Molecular Mass: 226.2489; Exact Mass: 226.0906; EA: C, 74.32; F, 8.4; H, 4.9; N, 12.38. Smiles: Cc1ccc2n(c1)cc(n2)c1ccccc1F; InChIKey: QYNFYZIMAREBRW-UHFFFAOYSA-N

A mixture of 5-methylpyridin-2-amine (150 mg, 1.39 mmol, 1.00 equiv), 2-bromo-1-(2-fluorophenyl)ethanone (301 mg, 191 μL, 1.39 mmol, 1.00 equiv) and sodium hydrogen carbonate (175 mg, 2.08 mmol, 1.50 equiv) in anhydrous toluene (6.00 mL) was stirred for 16 hours at 115 °C. As preparation for column chromatography (dry load), Celite was added (0.9 g) and the reaction mixture with Celite was evaporated. The obtained crude product was purified via flash-chromatography (Interchim devices puriFLASH 5.125) on silica gel (PF-15SIHP-F0040) using cyclohexane/ethyl acetate 0% to 30% ethyl acetate in 15 column volumes (1 column volume = 91.9 mL; flow: 26 mL/min). The isolated product 2-(2-fluorophenyl)-6-methyl-imidazo[1,2-a]pyridine (292 mg, 1.29 mmol) was obtained as a light orange solid in 93% yield. *R_f_* = 0.33 (cyclohexane/ethyl acetate 2:1).

^1^H NMR (400 MHz, Chloroform-d [7.27 ppm], ppm) δ = 8.37–8.32 (m, 1H), 7.95 (d, *J* = 4.0 Hz, 1H), 7.89 (t, *J* = 0.7 Hz, 1H), 7.53 (d, *J* = 9.2 Hz, 1H), 7.31–7.23 (m, 2H), 7.16–7.11 (m, 1H), 7.02 (dd, *J* = 1.7 Hz, *J* = 9.2 Hz, 1H), 2.30 (d, *J* = 1.0 Hz, 3H); ^13^C NMR (100 MHz, Chloroform-d [77.0 ppm], ppm) δ = 160.2 (d, *J* = 248.1 Hz), 143.9, 138.8 (d, *J* = 2.3 Hz), 128.7 (d, *J* = 8.5 Hz), 128.7 (d, *J* = 3.9 Hz), 128.1, 124.4 (d, *J* = 3.9 Hz), 123.3, 122.0, 121.6 (d, *J* = 12.3 Hz), 116.7, 115.6 (d, *J* = 21.6 Hz), 111.8 (d, *J* = 15.4 Hz), 18.0; ^19^F NMR (376 MHz, ppm) δ = -114.07; MS (EI, 70 eV, 40 °C), m/z (%): 260 (13), 226 (100) [M]^+^, 225 (12), 123 (10), 85 (13), 58 (17). HRMS–EI (C_14_H_11_N_2_F) *(m/z)*: [M]^+^ Calcd 226.0901; Found 226.0900; IR (ATR, ṽ) = 3157 (w), 3063 (w), 2944 (w), 2918 (w), 2894 (w), 2854 (w), 1579 (w), 1547 (w), 1534 (w), 1504 (w), 1477 (vs), 1442 (w), 1418 (m), 1366 (w), 1347 (s), 1276 (w), 1264 (w), 1213 (vs), 1198 (m), 1149 (w), 1106 (w), 1071 (m), 1028 (m), 949 (w), 936 (w), 844 (w), 810 (m), 798 (vs), 766 (vs), 744 (vs), 713 (vs), 671 (m), 664 (s), 619 (w), 574 (m), 530 (s), 513 (w) cm^–1^.

Additional information on the chemical synthesis is available via Chemotion repository:

<https://doi.org/10.14272/reaction/SA-FUHFF-UHFFFADPSC-QYNFYZIMAR-UHFFFADPSC-NUHFF-NUHFF-NUHFF-ZZZ>

Additional information on the analysis of the target compound is available via Chemotion repository:

<https://doi.org/10.14272/QYNFYZIMAREBRW-UHFFFAOYSA-N.1>

**2-(4-Fluorophenyl)-6-methyl-imidazo[1,2-a]pyridine** (**X20036**)

Name {X20036}: 2-(4-fluorophenyl)-6-methyl-imidazo[1,2-a]pyridine; Formula: C_14_H_11_FN_2_; CAS: - ; Molecular Mass: 226.2489; Exact Mass: 226.0906; EA: C, 74.32; F, 8.4; H, 4.9; N, 12.38. Smiles: Fc1ccc(cc1)c1cn2c(n1)ccc(c2)C; InChIKey: WLMPCCSMVOBMIN-UHFFFAOYSA-N

A mixture of 5-methylpyridin-2-amine (150 mg, 1.39 mmol, 1.00 equiv), 2-bromo-1-(4-fluorophenyl)ethanone (301 mg, 1.39 mmol, 1.00 equiv) and sodium hydrogen carbonate (175 mg, 2.08 mmol, 1.50 equiv) in anhydrous toluene (6.00 mL) was stirred for 16 hours at 115 °C. As preparation for the column chromatography (dryload), Celite was added (0.9 g) and the reaction mixture with Celite was evaporated. The obtained crude product was purified via flash-chromatography (Interchim devices puriFLASH 5.125) on silica gel (PF-15SIHP-F0040) using cyclohexane/ethyl acetate 0% to 20% ethyl acetate in 15 column volumes (1 column volume = 91.9 mL; flow: 26 mL/min). The isolated product 2-(4-fluorophenyl)-6-methyl-imidazo[1,2-a]pyridine (290 mg, 1.28 mmol) was obtained as a light orange solid in 92% yield. *R_f_* = 0.21 (cyclohexane/ethyl acetate 2:1).

^1^H NMR (400 MHz, Chloroform-d [7.27 ppm], ppm) δ = 7.91–7.85 (m, 2H), 7.85–7.82 (m, 1H), 7.66 (s, 1H), 7.50 (d, *J* = 9.2 Hz, 1H), 7.13–7.07 (m, 2H), 7.00 (dd, *J* = 1.7 Hz, *J* = 9.2 Hz, 1H), 2.28 (d, *J* = 1.2 Hz, 3H); ^13^C NMR (100 MHz, Chloroform-d [77.0 ppm], ppm) δ = 162.5 (d, *J* = 246.6 Hz, C_q_), 144.6 (C_q_), 144.5 (C_q_), 130.1 (d, *J* = 3.1 Hz, C_q_), 127.9 (CH), 127.5 (d, *J* = 8.5 Hz, 2C, CH), 123.2 (CH), 122.0 (C_q_), 116.6 (CH), 115.5 (d, *J* = 21.6 Hz, 2C, CH), 107.5 (CH), 18.0 (CH_3_); ^19^F NMR (376 MHz, ppm) δ = -114.43; MS (EI, 70 eV, 80 °C), m/z (%): 226 (100) [M]^+^, HRMS–EI (C_14_H_11_N_2_F) *(m/z)*: [M]^+^ Calcd 226.0901; Found 226.0902; IR (ATR, ṽ) = 1598 (w), 1551 (w), 1517 (w), 1506 (w), 1480 (m), 1422 (w), 1408 (m), 1381 (w), 1366 (w), 1343 (w), 1292 (w), 1256 (w), 1220 (m), 1208 (m), 1159 (w), 1149 (m), 1091 (m), 1058 (w), 1040 (w), 1011 (w), 969 (w), 950 (w), 936 (w), 840 (vs), 827 (s), 805 (vs), 756 (w), 748 (w), 735 (vs), 704 (vs), 674 (w), 645 (m), 630 (m) cm^–1^.

Additional information on the chemical synthesis is available via Chemotion repository:

<https://doi.org/10.14272/reaction/SA-FUHFF-UHFFFADPSC-WLMPCCSMVO-UHFFFADPSC-NUHFF-NUHFF-NUHFF-ZZZ>

Additional information on the analysis of the target compound is available via Chemotion repository:

<https://doi.org/10.14272/WLMPCCSMVOBMIN-UHFFFAOYSA-N.1>

**8-Chloro-2-(2-fluorophenyl)-6-methylimidazo[1,2-a]pyridine** (**X19729**)

Name {X19729}: 8-chloro-2-(2-fluorophenyl)-6-methylimidazo[1,2-a]pyridine; Formula: C_14_H_10_ClFN_2_; CAS: - ; Molecular Mass: 260.6940; Exact Mass: 260.0517; EA: C, 64.5; Cl, 13.6; F, 7.29; H, 3.87; N, 10.75. Smiles: Cc1cn2cc(nc2c(c1)Cl)c1ccccc1F; InChIKey: MNWHHCRXBACDMO-UHFFFAOYSA-N

A mixture of 3-chloro-5-methyl-pyridin-2-amine (150 mg, 1.05 mmol, 1.00 equiv), 2-bromo-1-(2-fluorophenyl)ethanone (228 mg, 145 μL, 1.05 mmol, 1.00 equiv) and sodium hydrogen carbonate (133 mg, 1.58 mmol, 1.50 equiv) in anhydrous toluene (6.00 mL) was stirred for 72 hours at 115 °C. As preparation for the column chromatography (dry load), Celite was added (0.9 g) and the reaction mixture with Celite was evaporated. The obtained crude product was purified via flash-chromatography (Interchim devices puriFLASH 5.125) on silica gel (PF-15SIHP-F0040) using cyclohexane/ethyl acetate with 0% to 25% ethyl acetate in 20 column volumes (1 column volume = 91.9 mL; flow: 26 mL/min). The isolated product 8-chloro-2-(2-fluorophenyl)-6-methylimidazo[1,2-a]pyridine (270 mg, 1.04 mmol) was obtained as an off-white solid in 99% yield. *R_f_* = 0.63 (cyclohexane/ethyl acetate 2:1).

^1^H NMR (400 MHz, Chloroform-d [7.27 ppm], ppm) δ = 8.58–8.54 (m, 1H), 8.11 (d, *J* = 3.9 Hz, 1H), 7.94 (t, *J* = 1.2 Hz, 1H), 7.44–7.35 (m, 2H), 7.27–7.22 (m, 2H), 2.41 (d, *J* = 1.0 Hz, 3H); ^13^C NMR (100 MHz, Chloroform-d [77.0 ppm], ppm) δ = 160.3 (d, *J* = 248.9 Hz, C_q_), 141.3 (C_q_), 139.5 (d, *J* = 2.3 Hz, C_q_), 129.3 (d, *J* = 3.9 Hz, CH), 129.1 (d, *J* = 8.5 Hz, CH), 126.9 (CH), 124.5 (d, *J* = 3.1 Hz, CH), 122.5 (C_q_), 122.3 (CH), 122.0 (C_q_), 121.2 (d, *J* = 12.3 Hz, C_q_), 115.5 (d, *J* = 22.3 Hz, CH), 113.3 (d, *J* = 15.4 Hz, CH), 18.0 (CH_3_); ^19^F NMR (376 MHz, ppm) δ = -114.18; MS (EI, 70 eV, 70 °C), m/z (%): 260/262 (100/31) [M]^+^, 259 (7), 228 (7), 225 (7), 130 (5), 120 (5). HRMS–EI (C_14_H_10_N_2_ClF) *(m/z)*: [M]^+^ Calcd 260.0511; Found 260.0510; IR (ATR, ṽ) = 3153 (vw), 2918 (w), 1735 (vw), 1693 (vw), 1636 (vw), 1581 (w), 1545 (w), 1528 (w), 1479 (s), 1441 (m), 1411 (m), 1349 (m), 1327 (w), 1272 (m), 1214 (s), 1196 (m), 1159 (w), 1150 (w), 1109 (w), 1088 (w), 1072 (s), 1040 (w), 1021 (m), 986 (w), 948 (w), 902 (s), 875 (w), 867 (w), 851 (w), 823 (vs), 809 (m), 768 (vs), 749 (vs), 742 (vs), 715 (vs), 674 (s), 636 (w) cm^–1^.

Additional information on the chemical synthesis is available via Chemotion repository:

<https://doi.org/10.14272/reaction/SA-FUHFF-UHFFFADPSC-MNWHHCRXBA-UHFFFADPSC-NUHFF-NUHFF-NUHFF-ZZZ>

Additional information on the analysis of the target compound is available via Chemotion repository:

<https://doi.org/10.14272/MNWHHCRXBACDMO-UHFFFAOYSA-N.1>

**2-Phenyl-6-(trifluoromethyl)imidazo[1,2-a]pyridine** (**X20048**)

Name {X20048}: 2-phenyl-6-(trifluoromethyl)imidazo[1,2-a]pyridine; Formula: C_14_H_9_F_3_N_2_; CAS: - ; Molecular Mass: 262.2299; Exact Mass: 262.0718; EA: C, 64.12; F, 21.73; H, 3.46; N, 10.68. Smiles: FC(c1ccc2n(c1)cc(n2)c1ccccc1)(F)F; InChIKey: JRNFAEHFFKHQIZ-UHFFFAOYSA-N

A mixture of 5-(trifluoromethyl)pyridin-2-amine (200 mg, 1.23 mmol, 1.00 equiv), 2-bromo-1-phenylethanone (246 mg, 1.23 mmol, 1.00 equiv) and sodium hydrogen carbonate (155 mg, 1.85 mmol, 1.50 equiv) in anhydrous toluene (6.00 mL) was stirred for 16 hours at 115 °C. As preparation for the column chromatography (dryload), Celite was added (0.9 g) and the reaction mixture with Celite were evaporated. The obtained crude product was purified via flash-chromatography (Interchim devices puriFLASH 5.125) on silica gel (PF-15SIHP-F0040) using cyclohexane/ethyl acetate 0% to 20% ethyl acetate in 20 column volumes (1 column volume = 91.9 mL; flow: 26 mL/min). The isolated product 2-phenyl-6-(trifluoromethyl)imidazo[1,2-a]pyridine (273 mg, 1.04 mmol) was obtained as a light yellow solid in 84% yield. *R_f_* = 0.52 (cyclohexane/ethyl acetate 2:1).

^1^H NMR (400 MHz, Chloroform-d [7.27 ppm], ppm) δ = 8.49–8.48 (m, 1H), 7.97–7.94 (m, 3H), 7.74 (dd, *J* = 0.6 Hz, *J* = 9.4 Hz, 1H), 7.52–7.42 (m, 2H), 7.40–7.35 (m, 1H), 7.33 (dd, *J* = 1.8 Hz, *J* = 9.5 Hz, 1H); ^13^C NMR (100 MHz, Chloroform-d [77.0 ppm], ppm) δ = 147.6 (C_q_), 145.2 (C_q_), 132.8 (C_q_), 128.8 (2C, CH), 128.6 (CH), 126.2 (2C, CH), 124.5 (q, *J* = 5.4 Hz, CH), 123.5 (q, *J* = 271.3 Hz, C_q_, *C*F_3_), 120.6 (q, *J* = 2.3 Hz, CH), 118.1 (CH), 116.9 (q, *J* = 33.9 Hz, C_q_), 109.2 (CH); ^19^F NMR (376 MHz, ppm) δ = -62.13; ^13^C NMR (100 MHz, Chloroform-d [77.0 ppm], ppm) δ = 128.9, 128.7, 126.3, 124.6, 120.7, 118.1, 109.2; ^13^C NMR (100 MHz, Chloroform-d [77.0 ppm], ppm) δ = 128.9, 128.7, 126.3, 124.6, 120.7, 118.1, 109.2; MS (EI, 70 eV, 60 °C), m/z (%): 262 (100) [M]^+,^ HRMS–EI (C_14_H_9_N_2_F_3_) *(m/z)*: [M]^+^ Calcd 262.0712; Found 262.0714; IR (ATR, ṽ) = 3132 (w), 3044 (w), 3023 (w), 1640 (w), 1581 (w), 1548 (w), 1506 (w), 1477 (w), 1449 (w), 1439 (w), 1385 (w), 1340 (m), 1330 (m), 1317 (m), 1269 (m), 1234 (m), 1197 (m), 1171 (s), 1140 (s), 1106 (vs), 1069 (m), 1052 (vs), 1031 (s), 939 (m), 914 (m), 871 (vs), 823 (vs), 776 (vs), 759 (m), 751 (w), 718 (vs), 690 (vs), 684 (vs), 670 (vs), 639 (s) cm^–1^.

Additional information on the chemical synthesis is available via Chemotion repository:

<https://doi.org/10.14272/reaction/SA-FUHFF-UHFFFADPSC-JRNFAEHFFK-UHFFFADPSC-NUHFF-NUHFF-NUHFF-ZZZ>

Additional information on the analysis of the target compound is available via Chemotion repository:

<https://doi.org/10.14272/JRNFAEHFFKHQIZ-UHFFFAOYSA-N.1>

**2-(2-Fluorophenyl)-6-(trifluoromethyl)imidazo[1,2-a]pyridine** (**X20047**)

Name {X20047}: 2-(2-fluorophenyl)-6-(trifluoromethyl)imidazo[1,2-a]pyridine; Formula: C_14_H_8_F_4_N_2_; CAS: - ; Molecular Mass: 280.2203; Exact Mass: 280.0624; EA: C, 60.01; F, 27.12; H, 2.88; N, 10.0. Smiles: Fc1ccccc1c1cn2c(n1)ccc(c2)C(F)(F)F; InChIKey: SEADNLGNBQIQGS-UHFFFAOYSA-N

A mixture of 5-(trifluoromethyl)pyridin-2-amine (200 mg, 1.23 mmol, 1.00 equiv), 2-bromo-1-(2-fluorophenyl)ethanone (268 mg, 170 μL, 1.23 mmol, 1.00 equiv) and sodium hydrogen carbonate (155 mg, 1.85 mmol, 1.50 equiv) in anhydrous toluene (6.00 mL) was stirred for 16 hours at 115 °C. As preparation for the column chromatography (dryload), Celite was added (0.9 g) and the reaction mixture with Celite were evaporated. The obtained crude product was purified via flash-chromatography (Interchim devices puriFLASH 5.125) on silica gel (PF-15SIHP-F0040) using cyclohexane/ethyl acetate 0% to 20% ethyl acetate in 20 column volumes (1 column volume = 91.9 mL; flow: 26 mL/min). The isolated product 2-(2-fluorophenyl)-6-(trifluoromethyl)imidazo[1,2-a]pyridine (303 mg, 1.08 mmol) was obtained as a light yellow solid in 88% yield. *R_f_* = 0.55 (cyclohexane/ethyl acetate 2:1).

^1^H NMR (400 MHz, Chloroform-d [7.27 ppm], ppm) δ = 8.52–8.51 (m, 1H), 8.36 (pseudo-dt, *J* = 2.0 Hz, *J* = 7.6 Hz, 1H), 8.15–8.14 (m, 1H), 7.73 (dd, *J* = 0.6 Hz, *J* = 9.4 Hz, 1H), 7.37–7.27 (m, 3H), 7.17 (ddd, *J* = 1.3 Hz, *J* = 11.5 Hz, *J* = 8.1 Hz, 1H); ^13^C NMR (100 MHz, Chloroform-d [77.0 ppm], ppm) δ = 160.4 (d, *J* = 249.7 Hz, C_q_), 144.5 (C_q_), 141.2 (d, *J* = 2.3 Hz, C_q_), 129.6 (d, *J* = 8.5 Hz, CH), 128.9 (d, *J* = 3.9 Hz, CH), 124.7 (q, *J* = 6.6 Hz, CH), 124.6 (d, *J* = 3.1 Hz, C_q_), 123.5 (q, *J* = 271.3 Hz, C_q_, *C*F_3_), 120.8 (q, *J* = 2.8 Hz, CH), 120.7 (CH), 118.1 (CH), 116.9 (q, *J* = 33.9 Hz, C_q_), 115.7 (d, *J* = 22.3 Hz, CH), 113.1 (d, *J* = 15.4 Hz, CH); ^19^F NMR (376 MHz, ppm) δ = -62.20 (s), -113.63–-113.71 (m); MS (EI, 70 eV, 40 °C), m/z (%): 280 (100) [M]^+^, HRMS–EI (C_14_H_8_N_2_F_4_) *(m/z)*: [M]^+^ Calcd 280.0618; Found 280.0618; IR (ATR, ṽ) = 3170 (w), 3048 (w), 3026 (w), 1650 (m), 1619 (w), 1581 (w), 1550 (w), 1483 (m), 1449 (w), 1438 (w), 1388 (w), 1351 (w), 1332 (m), 1310 (m), 1295 (m), 1251 (m), 1235 (m), 1204 (m), 1196 (s), 1157 (vs), 1142 (vs), 1116 (vs), 1108 (vs), 1071 (vs), 1054 (vs), 1033 (s), 941 (s), 870 (vs), 839 (s), 819 (vs), 805 (vs), 762 (vs), 741 (vs), 714 (vs), 670 (vs), 639 (s), 606 (s) cm^–1^.

Additional information on the chemical synthesis is available via Chemotion repository:

<https://doi.org/10.14272/reaction/SA-FUHFF-UHFFFADPSC-SEADNLGNBQ-UHFFFADPSC-NUHFF-NUHFF-NUHFF-ZZZ>

Additional information on the analysis of the target compound is available via Chemotion repository:

<https://doi.org/10.14272/SEADNLGNBQIQGS-UHFFFAOYSA-N.1>

**8-Methyl-2-phenyl-imidazo[1,2-a]pyridine** (**X19151**)

Name {X19151}: 8-methyl-2-phenyl-imidazo[1,2-a]pyridine; Formula: C_14_H_12_N_2_; CAS: - ; Molecular Mass: 208.2585; Exact Mass: 208.1000; EA: C, 80.74; H, 5.81; N, 13.45.

Smiles: Cc1cccn2c1nc(c2)c1ccccc1; InChIKey: CZYPXIUACURBEQ-UHFFFAOYSA-N

A mixture of 3-methylpyridin-2-amine (150 mg, 1.39 mmol, 1.00 equiv), 2-bromo-1-phenylethanone (276 mg, 1.39 mmol, 1.00 equiv) and sodium hydrogen carbonate (175 mg, 2.08 mmol, 1.50 equiv) in anhydrous toluene (4.00 mL) was stirred for 16 hours at 115 °C. As preparation for the column chromatography (dryload), Celite was added (0.9 g) and the reaction mixture with Celite were evaporated. The obtained crude product was purified via flash-chromatography (Interchim devices puriFLASH 5.125) on silica gel (PF-15SIHP-F0040) using cyclohexane/ethyl acetate 0% to 30% ethyl acetate in 20 column volumes (1 column volume = 91.9 mL; flow: 26 mL/min). The isolated product 8-methyl-2-phenyl-imidazo[1,2-a]pyridine (246 mg, 1.18 mmol) was obtained as a brown solid in 85% yield. *R_f_* = 0.55 (cyclohexane/ethyl acetate 2:1).

^1^H NMR (400 MHz, Chloroform-d [7.27 ppm], ppm) δ = 8.00–7.97 (m, 3H), 7.83 (s, 1H), 7.46–7.42 (m, 2H), 7.35–7.31 (m, 1H), 6.95 (td, *J* = 1.2 Hz, *J* = 6.8 Hz, 1H), 6.68 (t, *J* = 6.8 Hz, 1H), 2.68 (s, 3H); ^13^C NMR (100 MHz, Chloroform-d [77.0 ppm], ppm) δ = 146.1, 145.2, 134.0, 128.6 (2C), 127.7, 127.5, 126.1 (2C), 123.4, 123.3, 112.3, 108.5, 17.1; MS (EI, 70 eV, 30 °C), m/z (%): 208 (100) [M]^+^,. HRMS–EI (C_14_H_12_N_2_) *(m/z)*: [M]^+^ Calcd 208.0995; Found 208.0994; IR (ATR, ṽ) = 3128 (w), 3053 (w), 3034 (w), 2983 (w), 2953 (w), 2924 (w), 1764 (vw), 1628 (w), 1604 (w), 1497 (w), 1477 (w), 1445 (w), 1432 (w), 1381 (w), 1368 (m), 1350 (w), 1332 (w), 1302 (w), 1258 (m), 1207 (w), 1170 (w), 1081 (w), 1068 (w), 939 (w), 914 (w), 878 (vw), 782 (s), 773 (s), 755 (m), 720 (vs), 691 (vs), 677 (w), 649 (w), 619 (w), 603 (w), 547 (w), 524 (w), 510 (w) cm^–1^.

Additional information on the chemical synthesis is available via Chemotion repository:

<https://doi.org/10.14272/reaction/SA-FUHFF-UHFFFADPSC-CZYPXIUACU-UHFFFADPSC-NUHFF-NUHFF-NUHFF-ZZZ>

Additional information on the analysis of the target compound is available via Chemotion repository:

<https://doi.org/10.14272/CZYPXIUACURBEQ-UHFFFAOYSA-N.1>

References

1. Dolomanov O V, Bourhis LJ, Gildea RJ, Howard JAK, Puschmann H. OLEX2: a complete structure solution, refinement and analysis program. J Appl Cryst. 2009;42:339–41.

2. Sheldrick GM. SHELXT - Integrated space-group and crystal-structure determination. Acta Cryst. 2015;A71:3–8.

3. Sheldrick GM. Crystal structure refinement with SHELXL. Acta Cryst. 2015;C71:3–8.

**Supplementary Figure legends**

**Supplementary Fig. S1.** Volcano plots and Hallmark gene set analyses showing pathways targeted by X15695. **A-D,** Vertical left lanes are the RNA-seq analyses presented as Volcano plots of DEGs following treatment with X15695 vs vehicle and E_2_ + X15695 vs E_2_ and in MCF-7 cells **(A, B)** and in T47D **(C, D)**. Threshold of |LogFC| ≥ 1.0 and adj. p value ≤ 0.05 were used. **A-D,** Vertical right lanes are the Hallmark pathway analysis of DEGs comparing treatment with E_2_ + X15695 vs E_2_ and X15695 vs vehicle in MCF-7 cells **(A, B)** and in T47D cells (**C and D)**.

**Supplementary Figure S2.** X15695-mediated attenuation of ERα target gene expression and reactivation of p53. **A,** Heatmaps comparing the DEGs in the estrogen signaling pathway after treating MCF-7 and T47D cells with E_2_ and X15695. **B and C,** Quantitative RT-PCR showing the action of X15695 on a select number of E_2_ regulated genes in MCF-7 and T47D cells. Cells were treated with 1 µM X15695 in the presence and absence of 10 nM E_2_ for 16 h. The data represents the mean ± SEM (n =4; * p ≤ 0.05; ** p ≤ 0.01; *** p ≤ 0.001; **** p ≤ 0.0001; ns is not significant). **D,** Heatmaps comparing the DEGs in the p53 pathway after treating MCF-7 and T47D cells with E_2_ and X15695. **E and F,** Quantitative RT-PCR showing the action of X15695 on a select number of p53 target genes in MCF-7 and T47D cells. Cells were 1 µM X15695 in the presence and absence of 10 nM E_2_ for 16 h. The data represents the mean ± SEM (n =4; * p ≤ 0.05; ** p ≤ 0.01; *** p ≤ 0.001; **** p ≤ 0.0001; ns is not significant).

**Supplementary Figure S3.** X15695 alters the cellular localization of wild-type p53 but not mutant p53. **A and B,** Representative immunofluorescence microscopy images showing cellular localization of p53 and mortalin in MCF-7 **(A)** and T47D **(B)** cells treated with 1 µM X15695 or vehicle (DMSO) for 16 h. Staining was performed using anti-p53 (followed by incubation with goat anti-rabbit IgG, Alexa Fluor^TM^ 546 antibody - *red*), anti-mortalin (followed by goat anti-mouse IgG, Alexa Fluor^TM^ 488 antibody incubation - *green*) and DAPI (*light blue*).

**Supplementary Figure S4.** X15695 derivatives attenuate AR target gene expression. Quantitative RT-PCR was carried out on a select number of AR target genes in LNCaP cells. Cells were treated with 10 nM DHT and 5 µM X15695 and the indicated imidazopyridine compounds for 16 h. The results are the means ± SEM (n > 4; * p ≤ 0.05; ** p ≤ 0.01; **** p ≤ 0.0001; ns is not significant).

**Supplementary Figure S5**. X15695-mediated regulation of expression and cellular localization of p53 in prostate cancer cells. **A,** Western blot analyses of p53 levels in extracts of the prostate cancer cells 22Rv.1, LNCaP and LAPC-4 treated with the indicated imidazopyridines (1 µM each) for 48 h. The blots were carried out with cell lysates using anti-p53 and anti-β-actin antibodies. **B,** Immunofluorescence images showing the cellular localization of p53 after treating 22Rv.1, LAPC-4 and LNCaP cells with 1 µM X15695 or vehicle (DMSO) for the indicated time period. Staining was performed with anti-p53 antibody followed by incubation with goat anti-mouse IgG, Alexa Fluor^TM^ 488 antibody (*red*) and DAPI (*blue*).

**Supplementary Figure S6**. X15695 does not induce apoptosis in LNCaP and LAPC-4 cells. **A and B,** LNCaP and **C and D,** LAPC-4 cells were incubated in medium alone (-) or with the indicated concentrations of X15695, DMSO or Etoposide (ETP; 250 µM) for 48 h. Subsequently, cells were stained with Hoechst 33342 and propidium iodide (PI). Images were acquired using an automated microscopy and analyzed by the scanˆR software. **A and C,** Total cell number of LNCaP and LAPC-4 cells divided into living and dead cells after treatment with indicated concentrations. **B and D,** Different types of dead cells presented as percentage of the total cell number. The error bars are SEM values related to the total cell number (**A, C)** or the percentage of dead cells (**B, D)**.
