# Supplementary material for: Identification of an Imidazopyridine-based Compound as an Oral Selective Estrogen Receptor Degrader for Breast Cancer Therapy
Source: Cancer Res Commun. 2023 Jul 27;3(7):1378–96. doi: 10.1158/2767-9764.CRC-23-0111 (PMC10373600; doi:10.1158/2767-9764.CRC-23-0111)
Supplement: Supplementary Table S1 — Potency of inhibition of colony formation by different imidazopyridines in breast and prostate cancer cells. [file crc-23-0111-s02.docx]

**Supplementary Table S1.** Potency of inhibition of colony formation by different imidazopyridines in breast and prostate cancer cells.

| **Compounds** | **Breast Cancer (MCF-7)**  **ER^+^** | **Breast Cancer**  **(ZR75-1)**  **ER^+^** | **Breast Cancer**  **(T47D)**  **ER^+^** | **Breast Cancer**  **(MDA-**  **MB231) ER^-^** | **Prostate Cancer (22Rv.1)**  **AR^+^** | **Prostate Cancer (LNCaP)**  **AR^+^** | **Prostate Cancer (PC3)**  **AR^-^** | **Prostate Cancer (DU145)**  **AR^-^** |
| --- | --- | --- | --- | --- | --- | --- | --- | --- |
|  | **IC_50 (nM)_** | **IC_50 (nM)_** | **IC_50 (nM)_** | **IC_50 (nM)_** | **IC_50 (nM)_** | **IC_50 (nM)_** | **IC_50 (nM)_** | **IC_50 (nM)_** |
| **A4B17** | **1280** | **110** |  | **n.d** | **3400** | **1600** | **n.d** | **n.d** |
| X19729 | 20.8 |  |  |  | 140 |  |  |  |
| **X19728** | **6.0±2.8** | **39.2±24.3** | **153.3±67.9** | **n.d** | **48.5±13.3** | **n.d** |  | **n.d.** |
| **X19724** | **3.3±0.9** | **16.2±4.7** | **86.1±49.1** | **n.d** | **4.4** | **0.2** |  | **n.d** |
| X19727 | 19.2 |  |  |  | 280 |  |  |  |
| X19726 | 84.6 |  |  |  | n.d |  |  |  |
| X19725 | 66.9 |  |  |  | 651±168 | $<0.1$ |  |  |
| X19712 | 2846 |  |  |  | 2790 |  |  |  |
| X19718 | 720.2 |  |  |  | 3480 |  |  |  |
| X19719 | 12.9 | 19.3 | 100 |  | 530 |  |  |  |
| **X19720** | **14.5** |  |  |  | **4.4±1.6** | $\boldsymbol{<0.1}$ |  | **n.d.** |
| X19168 | 12.0 |  |  |  | 641.0±246.3 |  |  |  |
| X19167 | 486.2 |  |  |  | 1880 |  |  |  |
| **X19166/**  **X15696** | **7.9±1.9** | **10.2±2.7** | **44.7±11.4** | **n.d** | **22.5±6.6** | $\boldsymbol{<0.1}$ | **11930** | **3581** |
| X19148 | 228.3 |  |  |  | 2410 |  |  |  |
| X19151 | 135.6 |  |  |  | n.d. |  |  |  |
| X19147 | 18.3 |  |  |  | n.d. |  |  |  |
| **X15695** | 3.5±1.7 | 7.3±1.6 | 10.4±2.8 | 5540 | 40.2±16.7 | $14.1$ | 2040 | 1340 |
| X15689 | 24.1 |  |  |  | 1150 | 560 | 2370 | 3170 |
| X15688 | 213.3 |  |  |  | 4760 | 3200 |  |  |
| X15694 | 182.7 |  |  |  | 10130 | 1100 |  |  |
| X15692 | 54.4 |  |  |  | 520 | 1170 | n.d. | 15090 |
| X20034 | n.d |  |  |  | ≥ 9500 |  |  |  |
| X20035 | 1570 |  |  |  | n.d |  |  |  |
| X20036 | n.d |  |  |  | n.d |  |  |  |
| **X20046** | **10.0±4.3** | **31.8** | **57.1** | **n.d** | **7.5±3.2** | **0.1** | **n.d** | **n.d** |
| X20047 | 57.8 |  |  |  | 135.5 |  |  |  |
| X20048 | 589.4 |  |  |  | 561.8 |  |  |  |
