# Supplementary material for: Identification of an Imidazopyridine-based Compound as an Oral Selective Estrogen Receptor Degrader for Breast Cancer Therapy
Source: Cancer Res Commun. 2023 Jul 27;3(7):1378–96. doi: 10.1158/2767-9764.CRC-23-0111 (PMC10373600; doi:10.1158/2767-9764.CRC-23-0111)
Supplement: Supplementary Table S2 — Crystallographic data and structure refinement information for X15695, X15696 and X19168. [file crc-23-0111-s03.docx]

**Supplementary Table S2.** Crystallographic data and structure refinement information for X15695, X15696 and X19168.

| Compound | **X15695** | **X15696** | **X19168** |
| --- | --- | --- | --- |
| Empirical formula | C_14_H_7_ClF_4_N_2_ | C_14_H_8_ClF_3_N_2_ | C_13_H_8_N_2_FBr |
| Formula weight | 314.67 | 296.67 | 291.12 |
| Temperature/K | 180 | 150 | 150 |
| Crystal system | orthorhombic | monoclinic | monoclinic |
| Space group | *Pbca* | *P*2_1_/*c* | *P*2_1_/*c* |
| a/Å | 8.2266(2) | 10.6224(12) | 12.8788(6) |
| b/Å | 20.8314(5) | 15.1386(19) | 13.6172(9) |
| c/Å | 14.6999(4) | 8.1382(9) | 6.1409(3) |
| α/° | 90 | 90 | 90 |
| β/° | 90 | 108.692(8) | 94.721(4) |
| γ/° | 90 | 90 | 90 |
| Volume/Å^3^ | 2519.15(11) | 1239.7(3) | 1073.30(10) |
| Z | 8 | 4 | 4 |
| ρ_calc_g/cm^3^ | 1.659 | 1.590 | 1.802 |
| μ/mm^-1^ | 2.029 | 1.960 | 3.535 |
| F(000) | 1264.0 | 600.0 | 576.0 |
| Crystal size/mm^3^ | 0.24 × 0.22 × 0.20 | 0.15 × 0.13 × 0.03 | 0.14 × 0.03 × 0.02 |
| Radiation | GaKα (λ = 1.34143) | GaKα (λ = 1.34143) | GaKα (λ = 1.34143) |
| 2Θ range for data collection/° | 7.384–127.848 | 7.644–124.906 | 5.99–125.04 |
| Reflections collected | 13691 | 6889 | 6923 |
| Independent reflections | 3094 [R_int_ = 0.0173] | 2902 [R_int_ = 0.0536] | 2528 [R_int_ = 0.0128] |
| Ind. refl. with I ≥ 2σ(I) | 2744 | 2314 | 2360 |
| Data/restraints/parameters | 3094/0/218 | 2902/0/181 | 2528/0/154 |
| Goodness-of-fit on F^2^ | 1.059 | 1.159 | 1.073 |
| Final R indexes [I ≥ 2σ(I)] | R_1_ = 0.0407,  wR_2_ = 0.1158 | R_1_ = 0.0960,  wR_2_ = 0.2621 | R_1_ = 0.0223,  wR_2_ = 0.0630 |
| Final R indexes [all data] | R_1_ = 0.0443,  wR_2_ = 0.1183 | R_1_ = 0.1056,  wR_2_ = 0.2756 | R_1_ = 0.0237,  wR_2_ = 0.0636 |
| Largest diff. peak/hole / e Å^–3^ | 0.37/–0.41 | 1.44/–0.81 | 0.28/–0.42 |
| CCDC number | 2226035 | 2218538 | 2218539 |
