# Supplementary figures and images for: Identification of an Imidazopyridine-based Compound as an Oral Selective Estrogen Receptor Degrader for Breast Cancer Therapy
Source: Cancer Res Commun. 2023 Jul 27;3(7):1378–96. doi: 10.1158/2767-9764.CRC-23-0111 (PMC10373600; doi:10.1158/2767-9764.CRC-23-0111)

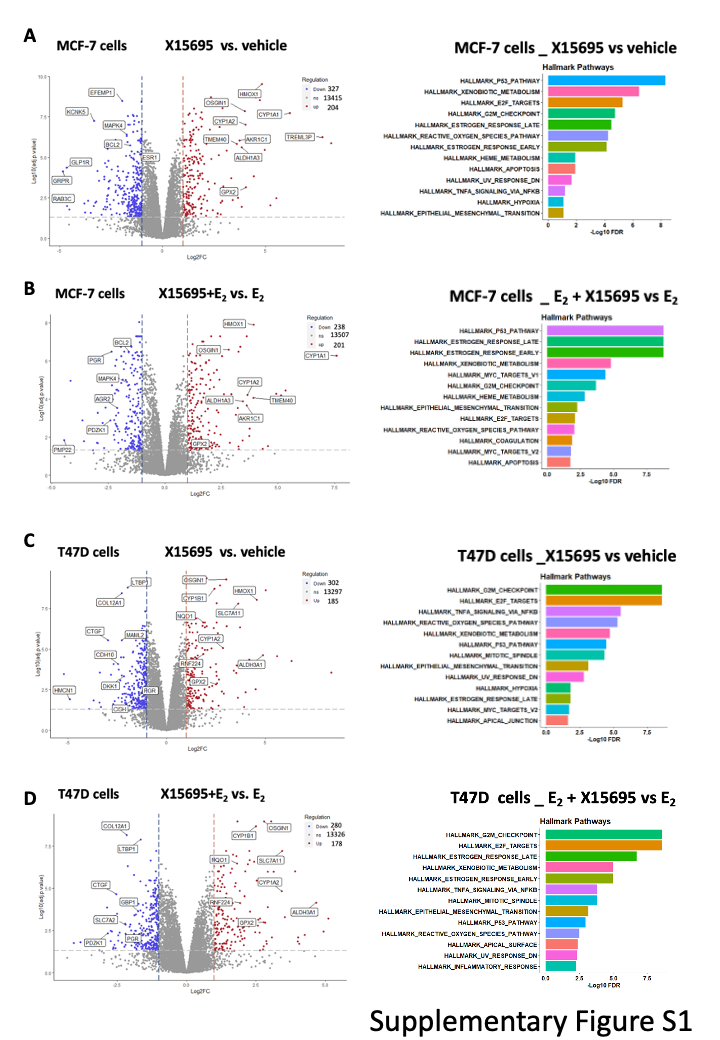

Supplement: Figure S1 — Volcano plots and Hallmark gene set analyses showing pathways targeted by X15695. [file crc-23-0111-s04.png]

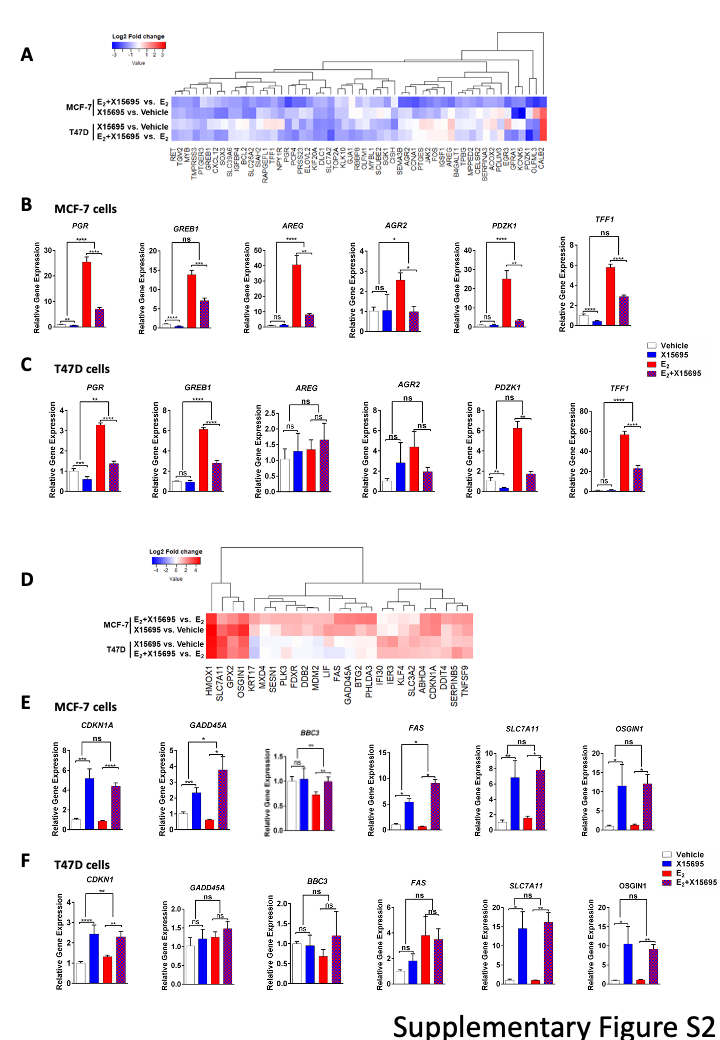

Supplement: Figure S2 — X15695-mediated attenuation of ERalpha target gene expression and reactivation of p53. [file crc-23-0111-s05.png]

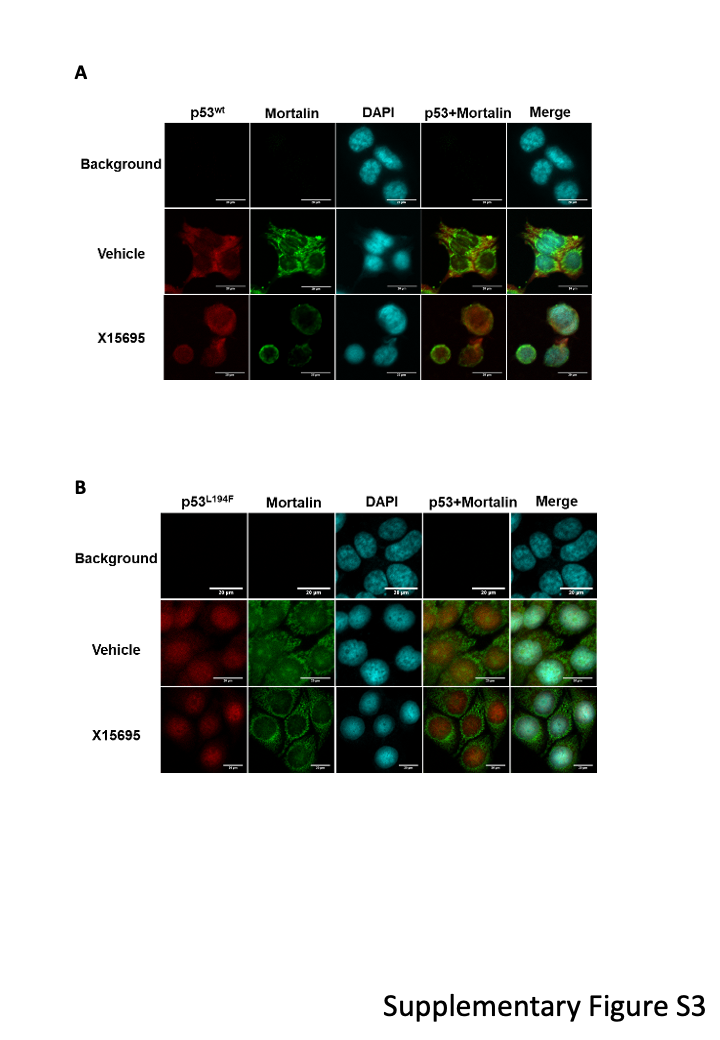

Supplement: Figure S3 — X15695 alters the cellular localization of wild-type p53 but not mutant p53. [file crc-23-0111-s06.png]

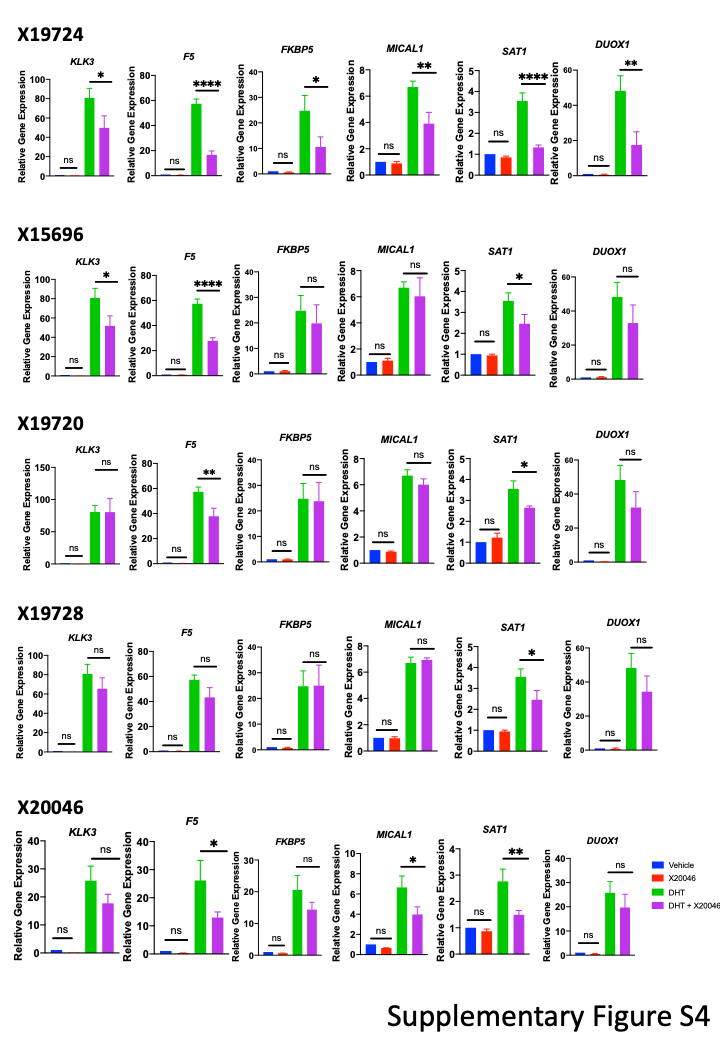

Supplement: Figure S4 — X15695 derivatives attenuate AR target gene expression. [file crc-23-0111-s07.png]

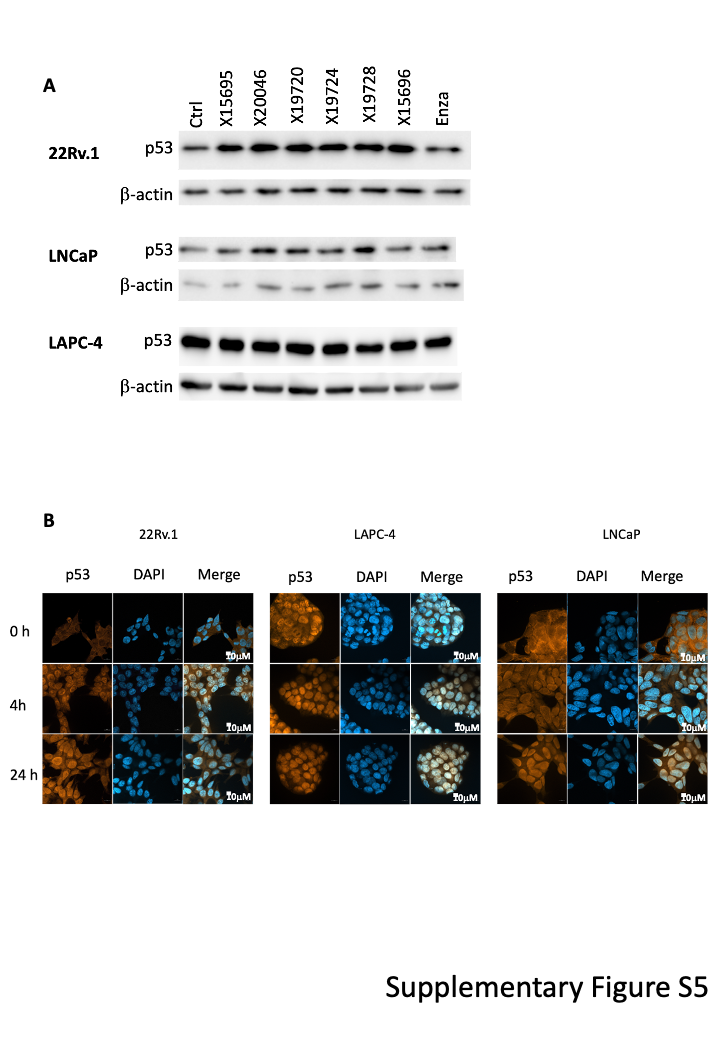

Supplement: Figure S5 — X15695-mediated regulation of expression and cellular localization of p53 in prostate cancer cells. [file crc-23-0111-s08.png]

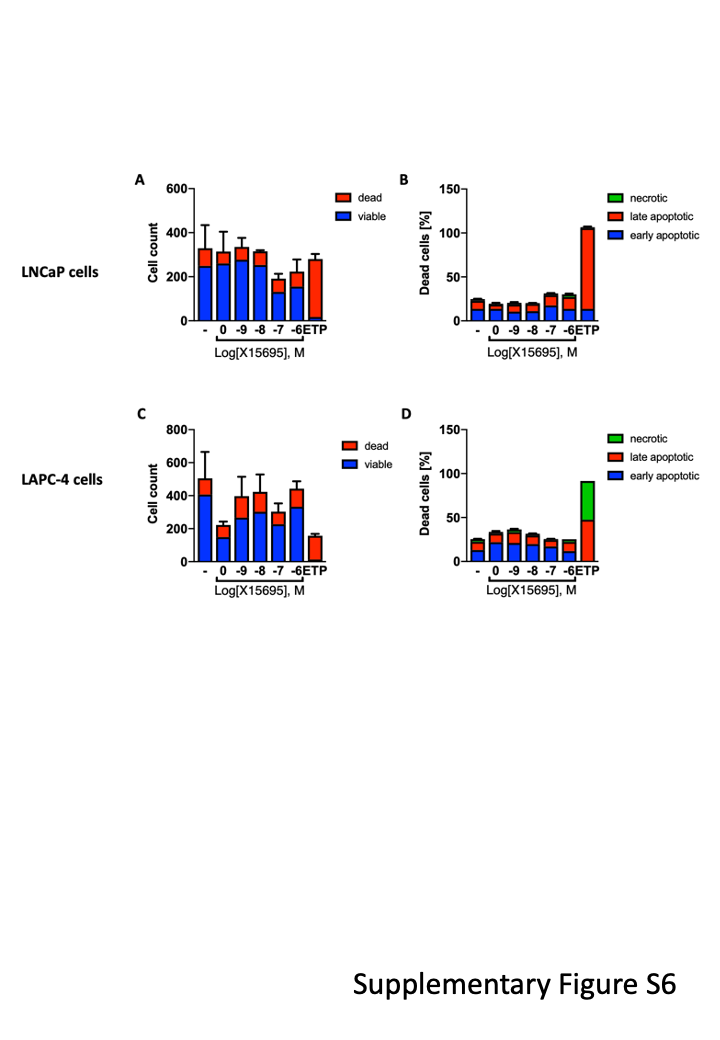

Supplement: Figure S6 — X15695 does not induce apoptosis in LNCaP and LAPC-4 cells. [file crc-23-0111-s09.png]
